# Synthesis of isoxazoles and their hydrazinolysis to 5-aminopyrazoles: an approach to fluorescent derivatives

**DOI:** 10.1039/d5ra09395c

**Published:** 2026-02-11

**Authors:** María-Camila Ríos, Alexander Ladino-Bejarano, Gian Pietro Miscione, Jaime Portilla

**Affiliations:** a Department of Chemistry, Universidad de Los Andes Carrera 1 No. 18A-10 Bogotá 111711 Colombia jportill@uniandes.edu.co

## Abstract

A protocol for the synthesis of 5-arylisoxazoles and their TFA-mediated hydrazinolysis to 5-amino-3-aryl-1*H*-pyrazoles was developed using readily available ketones as starting materials and eco-friendly microwave-assisted conditions. All reactions proceeded in high yields, and although some of the obtained heteroamines are commercially available, they are challenging to obtain because they are expensive or require access to equally expensive substrates. A computational study using TD-DFT calculations was conducted to better understand the reaction mechanism of the isoxazole ring-opening reaction with hydrazine in water. Ultimately, the photophysical properties of some fluorescent products were conveniently explored.

## Abstract 2

A TFA-mediated MW-assisted synthesis of 5-amino-3-(hetero)arylpyrazoles *via* 5-(hetero)aryl-isoxazoles as 1,3-bis-electrophylic substrates under eco-friendly conditions was developed, and the photophysics of some fluorescent derivatives were studied.

## Graphical abstract

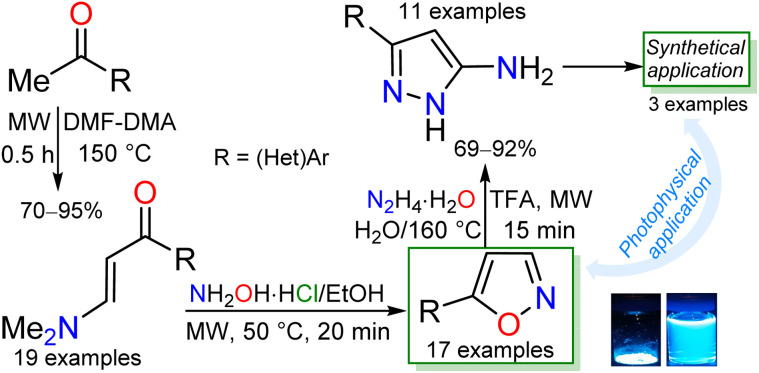

## Introduction

**Fig. 1:**
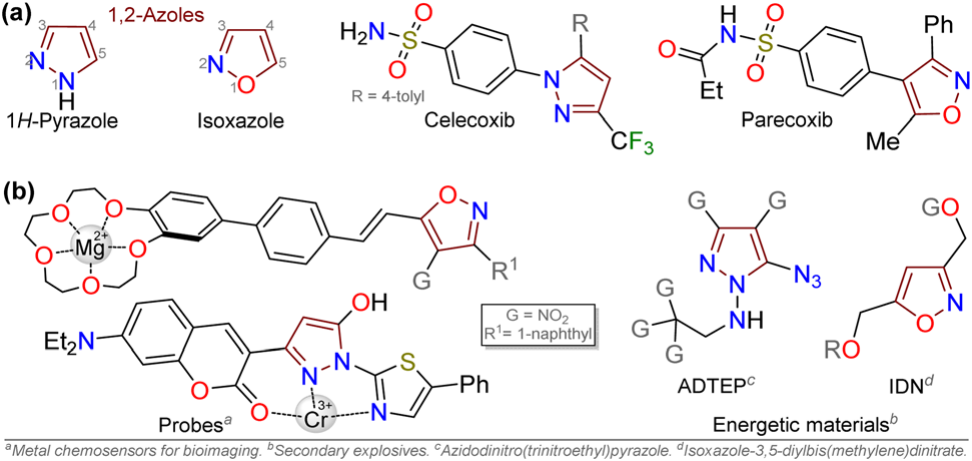
Pyrazoles and isoxazoles of (a) biological and (b) physical interest.

Pyrazole and isoxazole derivatives are an essential family of five-membered aza-heterocyclic compounds (AHCs) with pertinent applications in biological (medicinal, agrochemical, *etc.*), physical (materials science, optoelectronics, *etc.*), industrial (drugs, dyes, explosives, *etc.*), and synthetic fields.^1–6^ These 1,2-azoles are frequently obtained by cyclocondensation reactions of 1,3-bis-electrophiles with hydrazines or hydroxylamine, respectively, although at times, [3 + 2] cycloadditions have been used to access them.^3,7,8^ They are key intermediates to access more complex structures with better properties,^5,9,10^ and numerous derivatives have been used as anti-inflammatory, anti-cancer, antibiotic, and antifungal drugs, among others.^11–18^ They have also been found in fluorescent molecules^19–21^ and energetic materials^7,22,23^ (Fig. 1).

**Scheme 1:**
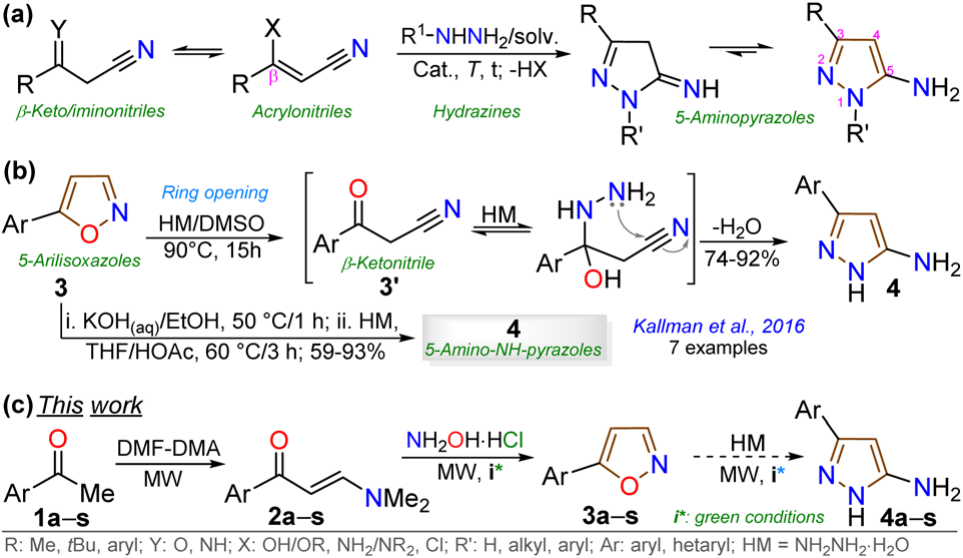
Synthesis of 3-substituted 5-aminopyrazoles using (a) acrylonitriles (*via* classical cyclocondensation) or (b and c) 5-arylisoxazoles (*via* opening ring).

Pyrazoles have shown greater synthetic weight than isoxazoles because of the higher reactivity of the π-excessive pyrazole ring compared to the one with the oxygen atom.^1–5,7^ In this respect, 5-aminopyrazoles are important as 1,3-bis-nucleophilic reagents in obtaining 5:6 aza-fused motifs (*e.g.*, pyrazolo[3,4-*b*]pyridines^24^ and pyrazolo[1,5-*a*]pyrimidines^6^) by their reaction with 1,3-bis-electrophiles, which are biologically and photophysically relevant AHCs.^6,24,25^ These amines are obtained by reacting hydrazines with acrylonitriles bearing an easily movable group at the Cβ (*e.g.*, β-enaminonitriles or enolizable β-ketonitriles) to yield the aromatic ring substituted with an amino group.^7^ For example, 5-amino-NH-pyrazoles are obtained from hydrazine monohydrate (HM). The acrylonitrile derivatives usually used (*i.e.*, X = NH_2_, OH, or Cl) are reactive substrates that exhibit synthetic (several-stage reactions), storage, and handling difficulties^7^ (Scheme 1a and 1b).

Although most reported syntheses of 5-aminopyrazoles use toxic, hazardous, or flammable reagents (*e.g.*, POCl_3_ in the synthesis of β-chloroacrylonitriles),^7,26^ their access *via* acrylonitriles remains the usual method.^7^ Thus, there is a need for efficient protocols that use easy-to-handle substrates. In 1941, Bell^27^ reported the hydrazinolysis of 5-methylisoxazole to form 5-amino-3-methyl-*N*-arylpyrazoles, *in situ* generating acetylacetonitrile; however, this method has been poorly studied due to limited access to diverse isoxazoles.^28,29^ In 2016, Kallman *et al.*^30^ reported a similar route to yield 5-amino-3-aryl-1*H*-pyrazoles 4 from 5-arylisoxazoles 3 and HM under basic conditions in one or two reaction stages (Scheme 1b); they followed up the reaction by NMR to establish the optimal conditions and the mechanism. Although this report is from almost a decade ago, this pyrazole type is still obtained from acrylonitriles,^1,2,7^ and protocols based on isoxazoles are rare,^31–34^ or are found in patents of diverse complex products.^35,36^ These results match the reduced substrates disposal or the reaction's scope, thereby limiting later transformations.^30–36^

**Scheme 2:**
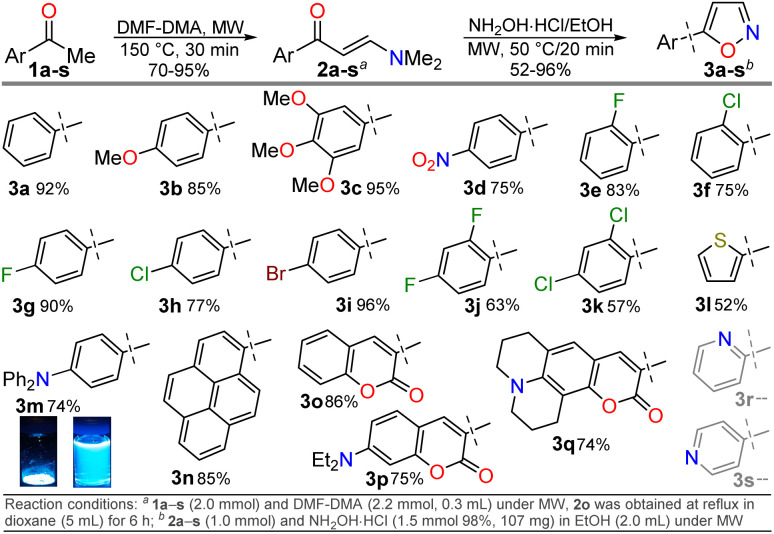
Synthesis of β-enaminones 2a–s and isoxazoles 3a–q.

The cited protocols have some operational shortcomings, such as poor to moderate yields and long reaction times (up to 2 days), requiring several reaction steps with solvents that can be difficult to remove and could originate contamination. Likewise, most of these methods require an excess of acid (HCl, HOAc, *etc.*) or base (KOH, MeONa, *etc.*), or even protective groups.^30–36^ Recently, our group synthesized and photophysically studied four fluorescent isoxazoles substituted with 1-pyrenyl (3n) and 3-coumarinyl (3o–q) rings (Scheme 2), which exhibited notable acidochromism in trifluoroacetic acid (TFA) *via* an intramolecular charge-transfer (ICT) process.^20,37^ This process is maybe the most relevant among molecular optical mechanisms, with several examples involving AHCs with diverse donor–acceptor molecular architecture.^20,38–41^ Dyes 3n–q were obtained by the microwave-assisted reaction of hydroxylamine hydrochloride (HH) with β-enaminones 2n–q; this substrate type has proved to be convenient to access isoxazoles 3,^20,37,42^ and other AHCs, and our group has developed numerous syntheses with them.^20,37,43,44^ Therefore, we aim to optimize the hydrazinolysis of a 5-arylisoxazole derivative to the respective 5-amino-NH-pyrazole under microwave (MW) conditions; then to obtain a broad family of isoxazoles 3a–s and evaluate their hydrazinolysis reaction to 5-aminopyrazoles 4a–s (Scheme 1c).

## Results and discussion

### Synthesis

We initiated the study by exploring a standard synthetic route to a family of 5-(hetero)arylisoxazoles under mild and eco-friendly conditions. First, β-enaminones 2a–s were obtained by a MW-assisted method, which is widely used in our laboratory^20,37,43,44^ or adapting other reported methodologies,^45,46^ see SI for details; β-enaminones 2a–s contain diverse (hetero)aryl groups and were obtained in high yields (70–95%). Then, the synthesis of isoxazoles 3a–s was explored by MW-assisted reactions of 2a–s with HH.^30,47^ This reaction was conducted in the absence of base to favor the 5-aryl regioisomer over the 3-aryl regioisomer obtained in basic media; this result is due to the nitrogen atom's lower nucleophilicity in acidic media, which favors initial attack by the oxygen atom on the carbonyl group.^30,47^ Such as 2a–s or the reported isoxazoles 3n–q,^20,37^ the electronic nature of substituents does not govern the reaction yields of 3a–q (52–96%). However, reactions do not occur with pyridyl-substituted reagents 2r and 2s, even under variable conditions (temperature, time, solvent, *etc.*), although 3r and 3s were previously reported.^48^ We assume the reaction does not proceed due to the poor solubility of precursors in the presence of HCl (from HH), as minimal solvent is used in the MW tubes and salts are formed; however, 3r and 3s could not be obtained under reflux^48^ so this report should be revised. Notably, the triphenylamine derivative 3m exhibited a higher fluorescence in both solution and the solid state than isoxazole 3n–q; thus, its photophysics are described in the last section (Scheme 2).

Subsequently, we envisaged that the MW-assisted hydrazinolysis reaction of 3a–q with HM could yield 5-amino-1*H*-pyrazoles 4a–q, given the proven advantages of the MW synthesis^20,37,43,44^ and difficulties shown by the cited methods.^30–36^ Thus, we aimed to optimize a protocol using the model substrate 3h and varying solvent, time, temperature, and the presence of an additive (Table 1). The first experiments were performed under conditions described by Kallman *et al.*,^30^ but under microwaves for 1 hour (Entries 1–3), offering very low reaction yields without significant consumption of the substrate. We then use other polar solvents, including EtOH, MeOH, and H_2_O, and even in the absence of any solvent at high temperatures (Entries 4 to 11), finding an increase in reaction yield, but without exceeding 50%. We observed higher yields when the reaction was carried out at 150 °C for 15 minutes; thus, the reaction was continued under these conditions.

**Table 1:** Optimization for the synthesis of the 5-amino-NH-pyrazole 6a^a^

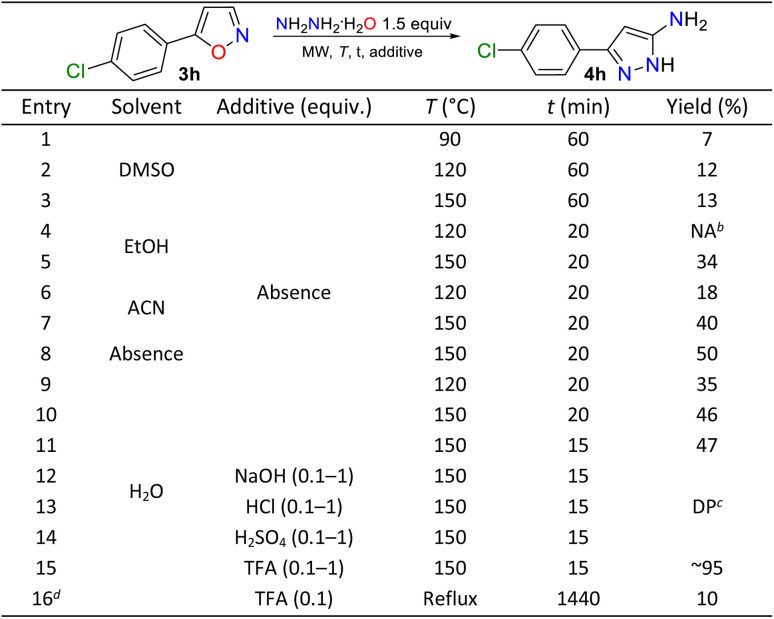

a Reaction conditions: 3 h (0.25 mmol, 44.9 mg) and HM (1.5 equiv, 19 µL) under MW.

b NA: Reaction does not advance.

c DP: Decomposition product.

d Under conventional heating.

In the study of improving yields in eco-friendly conditions, it was decided to carry out the reaction in water and in the presence of an additive, finding that decomposition products with 4h were generated in the presence of strong acids/bases, which did not allow proper isolation of the product (Entries 12 to 14). Finally, the reaction markedly improved when TFA was used (Entry 15), possibly due to its proven excellent interaction with the isoxazole rings.^20,37^ Thus, the optimal conditions for yielding 4h are those of entry 15: microwave irradiation in water with TFA as a catalyst at 150 °C for 15 minutes. Notably, the former product precipitates under these reaction conditions and can be recovered with good purity by simple filtration and washing (see SI for details).

**Scheme 3:**
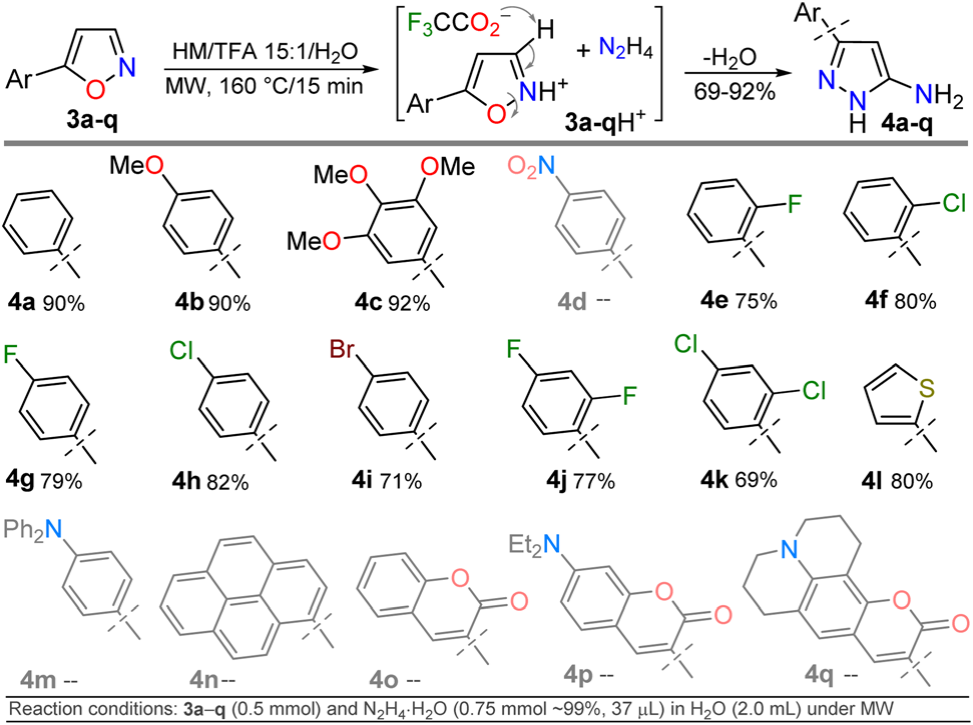
Synthesis of 5-amino-3-aryl-NH-pyrazoles 4a–o.

Once the optimal conditions for obtaining 4h were established, the scope of this synthesis was studied (Scheme 3). Initially, the introduction of diverse aryl groups and a π-excessive 2-thienyl ring was evaluated using substrates 3a–l. Unfortunately, it was not possible to obtain the nitro-derivative 4d due to the poor solubility in water of the substrate; possibly, the strong extractive nature of the nitro group into 3d disfavors the initial protonation of the azole nitrogen^20,37^ to solubilize it and start the reaction. By introducing fluorophoric groups in substrates (*i.e.*, TPA in 3m, 1-pyrenyl in 3n, or 3-coumarinyl in 3o–q) to obtain new pyrazoles useful in fluorophore discovery, none of the expected products were obtained; these results are possibly due to stereoelectronic factors, solubility in water,^20,37^ or decomposition (*e.g.*, hydrolysis) of the coumarin ring under the optimized reaction conditions. Thus, this reaction does not work with isoxazoles bearing strongly electron-donor or electron-acceptor groups, as they reduce the substrate's electrophilicity or basicity, respectively. For example, the nucleophilic attack of hydrazine on 4m does not occur, nor does the protonation of 4d to favor its solubility in water.

The obtained compounds (substituted β-enaminones 2a–s, 5-(hetero)arylisoxazoles 3a–q, and 5-aminopyrazoles 4a–c and 4e–l) were characterized by NMR spectroscopy and high-resolution mass spectrometry (HRMS). Notably, not all quaternary carbon atoms for the 5-(2-haloaryl)isoxazoles 4e,f and 4j,k could be observed *via*^13^C NMR spectra due to their multiple conformers and tautomers in equilibrium (see SI for details, Fig. S3–S41).

### Computational DFT study

To gain deeper insight into the reaction mechanism of the TFA-catalyzed synthesis of 5-aminopyrazoles 4a–l, we investigated the pathway leading to 4h using density functional theory (DFT) simulations. These ground-state calculations were performed with the Gaussian 16,^49^ software package at the M08-HX/6-311G* level of theory. The M08-HX functional^50^ was chosen for its high accuracy in describing reaction mechanisms, barrier heights, proton-transfer processes, and non-bonding interactions, which characterize this process.^51–53^ The 6-311G* basis set provides a balanced and well-established compromise between accuracy and computational cost for geometry optimizations and barrier heights. To represent the experimental environment, an implicit solvation model based on density (SMD) was used in water. The theory set used has proven to offer exceptional results in other mechanisms studied by us^44,54^ (Fig. S49; see SI for more details).

**Scheme 4:**
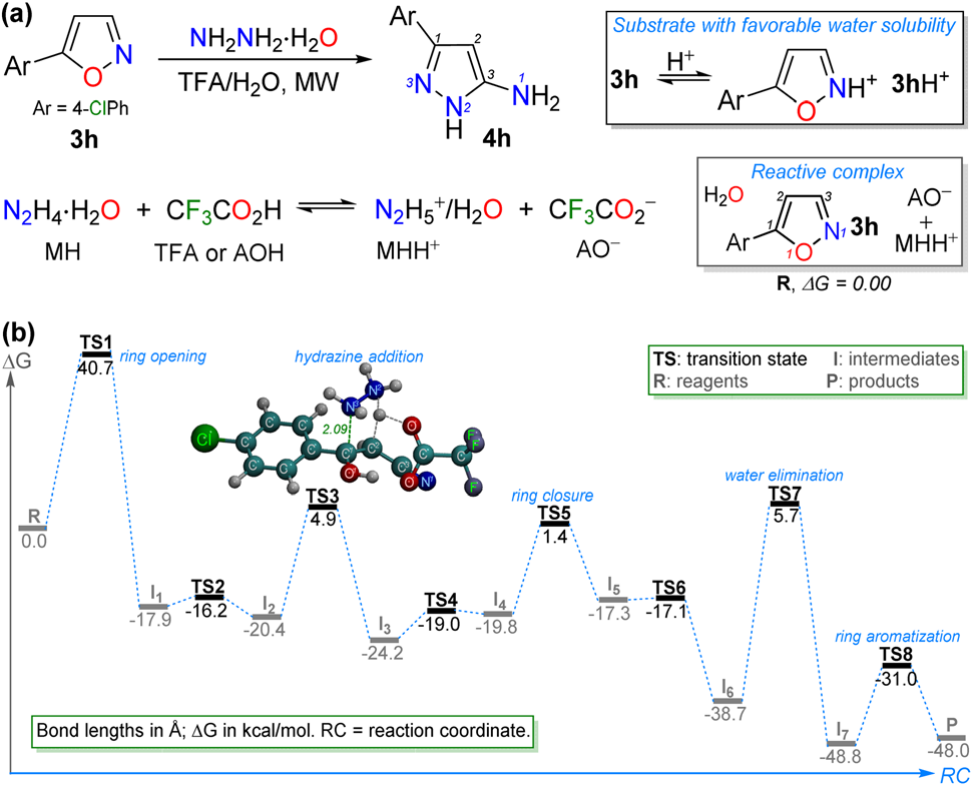
(a) Mechanistic considerations and (b) energetic profile for the TFA-mediated formation of the 5-aminopyrazole 4h in water.

**Scheme 5:**
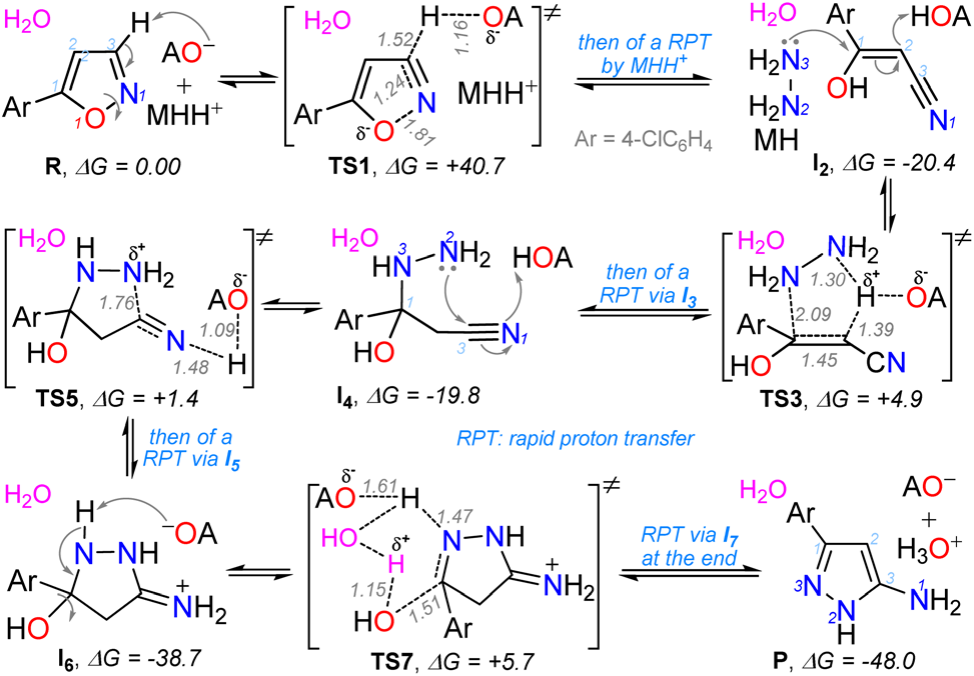
Essential steps of the proposal mechanism for the formation of 4h.

Considering the p*K*_a_ values of reagents, an aqueous acid–base equilibrium with the respective conjugated species (*i.e.*, CF_3_COO^−^ and N_2_H_5_^+^) can be given (see SI for details). In addition, the special interaction of the isoxazole ring with an acid medium (*i.e.*, TFA),^20,37^ could favor the substrate solubility. Thus, as the asymptotic limit (reactant energy) of the reaction profile, the reactive complex R was studied; this complex has a molecule of 3h, a trifluoroacetate anion (AO^−^), and a hydrazinium cation (MHH^+^); a water molecule can be included in the entire mechanism to explain the process (Schemes 4, 5 and S1).

The complete mechanism for the formation of 4h is described in the SI, and the essential steps are detailed below (Scheme 5). The first stage involves isoxazole ring opening at the transition state TS1 (40.7 kcal mol^−1^), which is the reaction-determining step, followed *via* a rapid proton transfer to yield the acrylonitrile intermediate I_2_. The high energy of TS1 is consistent with the experimental results, as an elevated temperature (160 °C) was required for the reaction; however, it should be noted that the short reaction time observed (15 min) is characteristic of MW-assisted reactions.^20,37,43,44^ The latter relevant step produces the cyclization intermediate I_4_ upon conjugated hydrazine addition to I_2_*via*TS3 (4.9 kcal mol^−1^), followed by a second rapid proton transfer (RPT). In the third key step, the cyclization of I_4_ forms the new ring in I_6_ (highly stable, −38.7 kcal mol^−1^) *via* the transition state TS5 (1.4 kcal mol^−1^) and a third RPT (Schemes 4b and 5). Finally, the removal of a water molecule in I_6_ through TS7 (5.7 kcal mol^−1^) and a fourth rapid proton transfer allows the system to evolve into the final pyrazole structure in P with aromaticity restored, now 48.0 kcal mol^−1^ more stable than the reactive complex (Schemes 4b, 5 and S1; see SI for more detail).

### Photophysical results

Recently, we have established that the four isoxazoles 3n–q (1-pyrenyl and 3-coumarinyl derivatives) are fluorescent molecules with noticeable photophysical properties in both solution and the solid state.^20,37^ Here, we obtained the TPA derivative 3m, studied its photophysics, and compared the results with those of 3n–q and the standard Prodan, discovering that 3m exhibits the best properties. The absorption and emission spectra of the six dyes were measured in 12 solvents of varying polarity at ∼20 °C and 27 µM (Table 2). No significant changes in the absorption maxima were observed as solvent polarity increased for all dyes (Table S22 and Fig. S42a); however, by averaging the values obtained for the absorptivity coefficient (*ε*) of isoxazoles 3m–q, we found that they showed higher *ε* values than Prodan. Notably, the *ε* value increases with the electron-donor nature of the fluorophoric ring in the dye due to improved intramolecular charge transfer (ICT) phenomena from such donor groups towards the nitrogen atom of the isoxazole ring, π-deficient in character. In addition, this ICT process favors the π-extended system, which offers improved *ε* values^20,38,55,56^ (Table 2, see resonant structures of 3m).

**Table 2:** Summary of photophysical properties of 3m–q and Prodan^a^

Fluorophore	*λ*_abs_, *λ*_em_ (nm); range	*ε*, *ϕ*_F_ (%)	SS, Δ*µ* (D)	LOD_F_ (µM), *ϕ*_F_^b^ (%)	Ref.
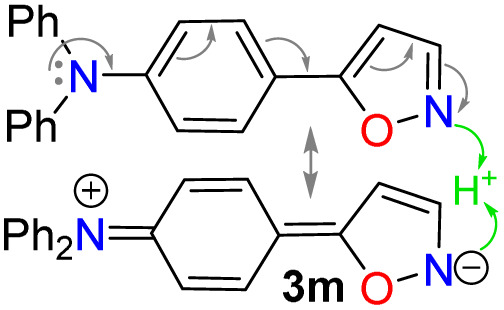	343–353, 406–458	44 780, 70 in DCM	115 in ACN, 14.80	0.67^turn-off^ (456 nm), 90^b^	This work
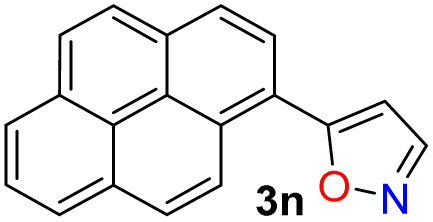	348–369, 394–404	18 820, 95 in acetone	60 in DMF, 4.67	2.41^turn-on^ (350 nm), 23^b^	2025 (ref. 37)
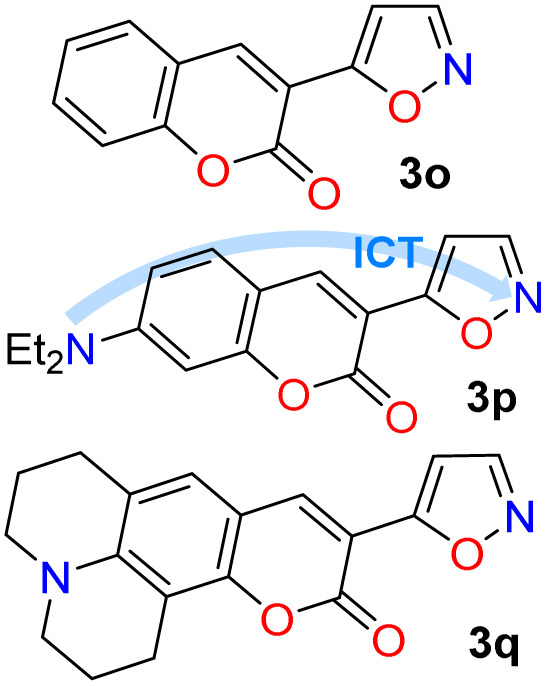	329–358, 402–416	16 220, 8 in toluene	79 in CHCl_3_, 3.51	—, 64^b^	2025 (ref. 20)
	343–353, 406–458	36 080, 36 in DMF	63 in DMSO, 8.37	3.38^turn off^ (482 nm), 49^b^	
	343–353, 406–458	53 600, 25 in ACN	49 in acetone, 5.75	2.04^turn-off^ (498 nm), —	
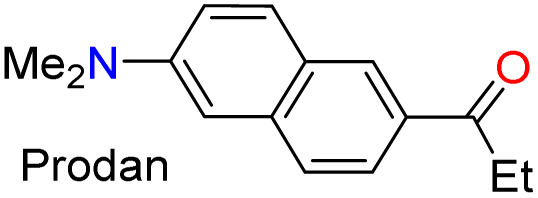	346–367, 418–502	14 080, 94 in ACN	135 in MeOH, 4.3 (ref. 59)	—, 35^b^	

a Experiments at 20 °C, a concentration of 27 µM, and using Prodan as a using Prodan as a standard. The average value of *ε* (in M^−1^ cm^−1^), the highest value of *ϕ*_F_, the highest value of Stokes Shifts (SS, *λ*_em_ – *λ*_abs_ in nm), the change of dipolar moment (Δ*µ*) value by Lippert–Mataga analysis, and limits of detection (LODs) for acidochromism (by TFA in ACN) are shown.

b Data *ϕ*_F_ in the solid state are shown.

**Fig. 2:**
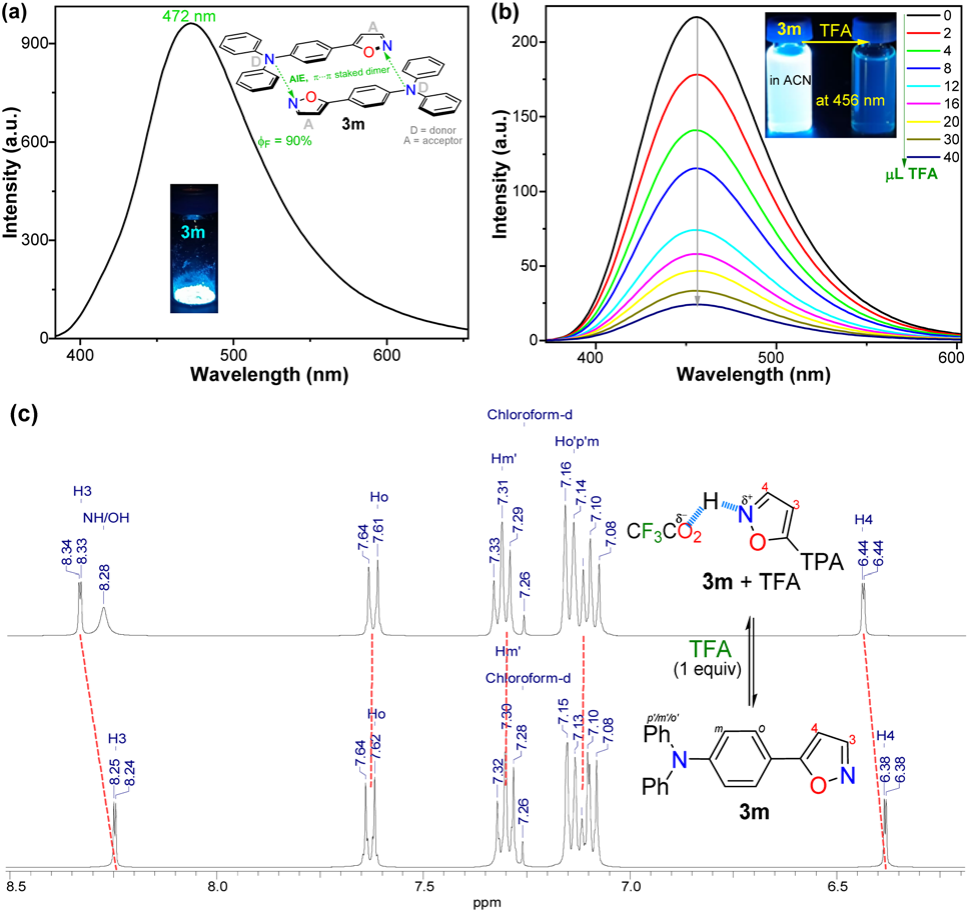
Emission spectra of the fluorophore 3m (a) in the solid state and (b) in MeCN with TFA (0–40 µL) at ∼20 °C. Photographs taken under a 395 nm UV lamp are shown. (c) ^1^H NMR spectra of 3m upon the addition of TFA.

The emission properties of 3m–q and Prodan evidenced that 3m exhibits the maximum emission brightness (*ε* × *ϕ*_F_) values with the following cases: 3m 24 310 in dichloromethane (DCM) > 3q 10 930 in acetonitrile (ACN) > 3p 9200 in *N*,*N*-dimethylformamide (DMF) > Prodan 6560 in ACN > 3n 3900 in acetone > 3o 1320 in toluene (Tables 2, S22 and Fig. S42b). These results are consistent with the notable ICT process in 3m, and the greater change of dipolar moment (Δ*µ*) value in its excited state with respect to the ground state (Δ*µ* = 14.8 D), which was determined from Lippert–Mataga analysis^57^ (Table 2 and Fig. S43, see SI for details). It is essential to note that a positive solvatofluorochromism behavior was observed in the emission data for the most polar dyes (*i.e.*, 3m, 3p, 3q, and Prodan), in which the redshift is evident as the solvent polarity increases; however, 3n and 3o showed no trend in this property due to their lower polarity.^20,37^ In addition, 3m exhibited a higher fluorescence quantum yield (*ϕ*_F_) in the solid state (*ϕ*_F_ = 90%) than 3m–q and Prodan (*ϕ*_F_ of 23–64%); perhaps the superior intermolecular interaction of 3m in the solid state, due to its high polarity, favors an increased aggregation-induced emission (AIE) process (Table 2, Fig. 2a and S44a).

The aggregation properties of 3m in solution were evaluated by absorption and emission spectroscopy (see SI for details), and the results were consistent with those in the solid state. For example, upon additing water (up to 50% v/v) to 3m in ACN, the absorption band at 344 nm decreased by up to 33% due to poor dye solubility in water (Fig. S44b). However, the emission band was redshifted (from 456 nm to 473 nm) as the water fraction increased (Fig. S44c), suggesting that water promotes the excimer formation^37,58^ of 3m*via* supramolecular interactions that induce aggregation in solution. Notably, emission bands in the aggregate state coincide in both the solution (*λ*_em_ = 472 nm, Fig. S44c) and the solid state (*λ*_em_ = 472 nm, Fig. 2a). Thus, the results for the aggregation of 3m in solution validate our assumptions regarding its supramolecular interactions in the solid state.

To complete the photophysical analysis of 3m, its acidochromism was studied by monitoring its absorption and emission spectra (at 27 µM in ACN) upon the addition of different amounts of TFA (0.1 M in ACN) to the probe (Table 2). Such as for 3n–q,^20,37^ it can be observed that changes in the absorption spectra are negligible and difficult to detect as the amount of TFA increases (Fig. S45a). Likewise, a noticeable change is observed in the emission spectra, with fluorescence turning off, exhibiting a limit of detection (LOD) of 0.67 µM (Fig. 2b and S45b). Although the binding mechanism of 3m–q to TFA is similar and involves the azolic nitrogen atom (=N…H^+^, established by ^1^H NMR titration of 3n–q in CDCl_3_),^20,37^ the new dye 3m is the most sensitive probe (Table 2). The binding mechanism for 3m was verified by ^1^H NMR spectra in CDCl_3_ with 1 equiv. of TFA, as this medium shifted the signals of the isoxazole ring (H3/H4, Fig. 2c and S46) to downfield, and the TPA moiety signals remained unchanged. Thus, the notable photophysics of 3m in solution and in the solid state would favor its performance in chemosensors and materials science applications.

**Scheme 6:**
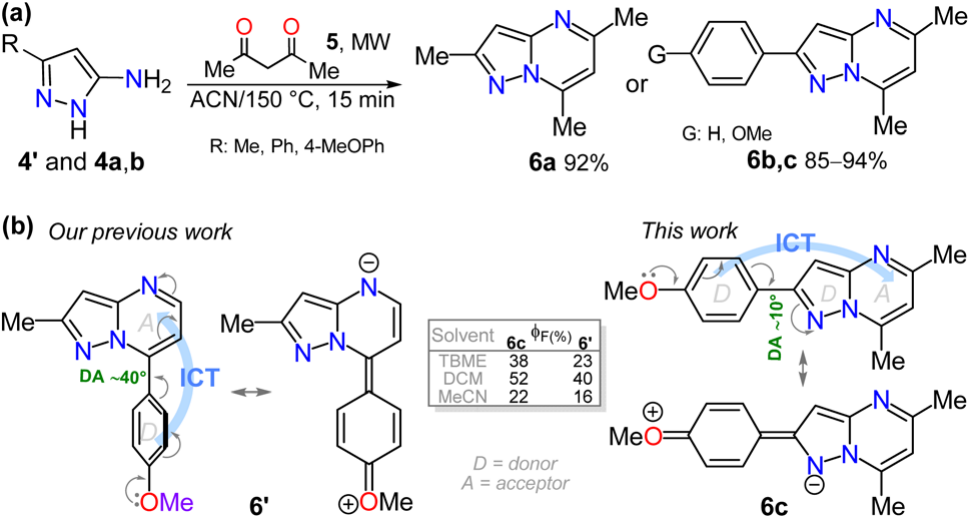
(a) Synthesis of 6a–c and (b) 4-anysyl substituted dyes 6′ and 6.

Finally, as none of the aminopyrazoles 4a–l are fluorescent dyes, pyrazolopyrimidines 6a–c were obtained using the MW-assisted reaction of 4′ or 4a,b with 2,4-pentanedione (5),^43^ to examine a synthetic application of 4a–l (Scheme 6a) and the photophysics of 6a–c. We have found that the 4-methoxyphenyl (4-anysyl) group favors the 7-(4-anysyl)-2-methylpyrazolo[1,5-*a*]pyrimidine (6′) fluorescence *via* ICT phenomena.^6,55,56^ Thus, the photophysics of the 2-(4-anisyl) derivative 6c was examined and compared with those of 6′,^56^ and the reference dyes 6a,b, seeing that the variance in dihedral angles (DA) of 6c and 6′ around their 4-anysyl group affects this property (Table S24 and Scheme 2b, see SI for more detail). The absorption and emission (Fig. S47) spectra of 6a–c were measured in different-polarity solvents, showing 6c a more intense ICT absorption band due to its 4-anysyl group. These dyes emit at ∼440 nm without any solvatofluorochromic behavior, being 6b,c more fluorescent (*ϕ*_F_ of 17–52%) than 6a (*ϕ*_F_ = 4–17%).

The photophysical data of 6c were also compared with those of 6′ under similar conditions^56^ (Table S24 and Fig. S48), finding that most of the data were in the same range; however, 6c displayed quantum yield values slightly higher (22–52%) than 6′ (16–40%),^56^ perhaps due to the greater directionality in the ICT process of 6c, slightly reduced by the electron-donor pyrazole moiety (Scheme 6b). Finally, TD-DFT calculations of electronic structure for 6a–c and 6′ were conducted to further understand their photophysical properties. Studies were executed using ORCA 5.0.3,^60,61^ with the B3LYP hybrid functional and the def2-TZVP/C basis set^62,63^ due to the notable performance of this software on similar structures studied by us in this context^6,38,43,56^ (Fig. S49; see SI for more details). This study validates that the dihedral angles in probes affect their electronic properties. Likewise, the energy gap values of these probes revealed that the presence of the 4-anisyl group in 6c and 6′ reduces this energy by facilitating the ICT process; however, the presence of the 4-anisyl group at position 7 favors a better π-extended conjugation. Thus, the final studies revealed that 6b,c and other analog compounds synthesized from 5-aminopyrazoles 4a–l exhibit high photophysical potential.

## Conclusions

In summary, a family of 5-(hetero)arylisoxazoles and of 5-amino-3-(hetero)aryl-NH-pyrazoles were synthesized under microwave conditions. Heteroamines were obtained using a TFA-catalyzed hydrazinolysis reaction, and both 1,2-azoles syntheses evidenced advantages against the other reported methods; *e.g.*, reduced reaction times, simple operation, good yields (52–95%), eco-compatibility (reactions in water), and using simpler and cheaper reagents – *e.g.*, β-anaminones used as starting materials were easily accessible from (hetero)aryl methyl ketones. However, the hydrazinolysis reaction does not work with isoxazoles bearing strongly electron-donating or electron-accepting groups, as these groups reduce the electrophilicity or basicity of the substrate, respectively. Moreover, the synthetic application of the obtained amines was developed to access pyrazolo[1,5-*a*]pyrimidine-based dyes; these dyes and the TPA-substituted isoxazole were photophysically studied, and results were compared with those of other reported analog dyes. The compounds described herein exhibited better photophysical properties than those of other reported fluorophores by us. Notably, mechanisms for accessing 5-aminopyrazoles *via* isoxazole ring-opening with hydrazine, and the photophysics of the pyrazolo[1,5-*a*]pyrimidine derivatives studied herein, were investigated using DFT calculations.

## Experimental section

*Comment*. Structures of some relevant or novel intermediates and products were determined by NMR and HRMS (Fig. S3–S41). See SI for experimental procedures and characterization data for all compounds, including the general information. In addition, the SI contains information for the photophysics experiments.

## Conflicts of interest

The authors declare no competing financial interest.

## Author contributions

The individuals listed as authors have contributed to the development of this manuscript, and no other person was involved. The authors' contributions included: M.-C. R. and A. L.-B. developed experiments (synthesis and photophysical studies) and literature review, M.-C. R. and G. P. M. carried out the computational research (calculations, analysis, and writing), while J. P. developed and composed the original draft, supervised it, and provided sources. All authors have read and agreed to the published version of this manuscript.

## Note added after first publication

This article replaces the version published on 11 February 2026 which contained errors in Table 2.

## Supplementary Material

RA-016-D5RA09395C-s001

## Data Availability

The data supporting the findings of this search are available within the article and its supplementary information (SI). Supplementary information: supporting data for this article are provided in the SI, which includes experimental procedures and characterization data, HRMS analysis with spectra, NMR spectra, and computational and photophysical details. See DOI: https://doi.org/10.1039/d5ra09395c.
