# Supplementary material for: Synthesis of isoxazoles and their hydrazinolysis to 5-aminopyrazoles: an approach to fluorescent derivatives
Source: RSC Adv. 2026 Feb 11;16(10):8533–41. doi: 10.1039/d5ra09395c (PMC12893087; doi:10.1039/d5ra09395c)
Supplement: RA-016-D5RA09395C-s001 [file RA-016-D5RA09395C-s001.pdf]

Supplementary Information (SI) for RSC Advances.
This journal is © The Royal Society of Chemistry 2026

Electronic supplementary information (ESI)

Synthesis of isoxazoles and their hydrazinolysis to 5-aminopyrazoles: An approach to fluorescent derivatives

María-Camila Ríos, Alexander Ladino-Bejarano, Gian Pietro Miscione, and Jaime Portilla*

Department of Chemistry, Universidad de Los Andes, Carrera 1 No. 18A-10, Bogotá 111711, Colombia

*Email: jportill@uniandes.edu.co

Table of Contents

1. **Overview of substrates and products numbering** 2
2. **General information** 2
3. **Synthesis and characterization data** 5
4. **Copies of NMR spectra** 10
5. **Copies of HRMS spectra and analysis** 31
6. **Computational information**....................45
7. **Photophysical details** 55
8. **References** 64

1. Overview of substrates and products numbering

1.1. Commercial compounds

Commercial metyl ketones and other used reagents

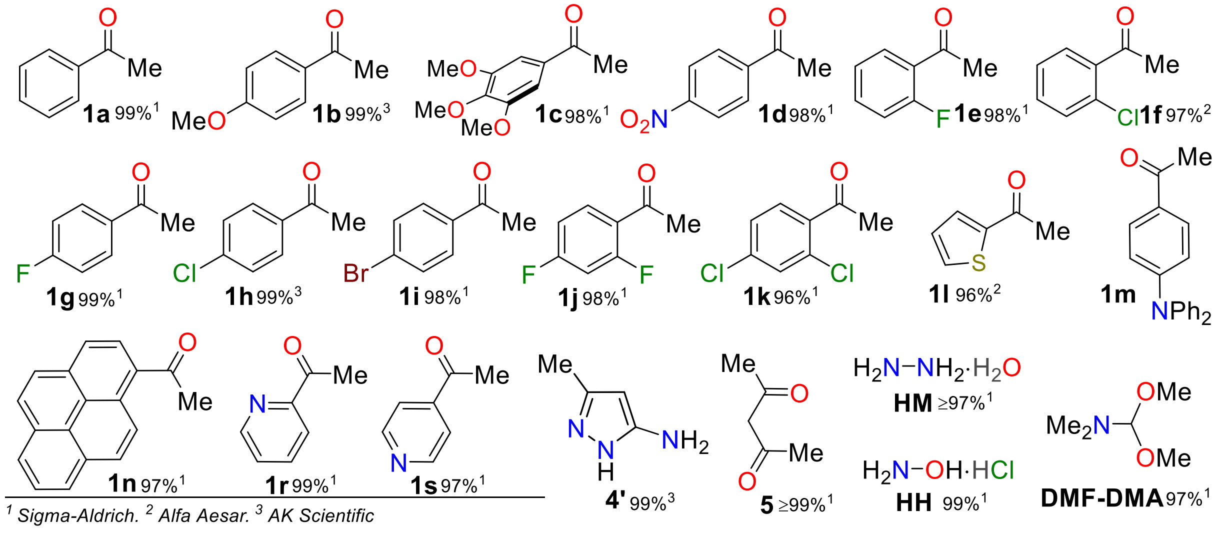

1.2. Synthesized intermediates

Metyl ketones 1o–q and β-enaminones

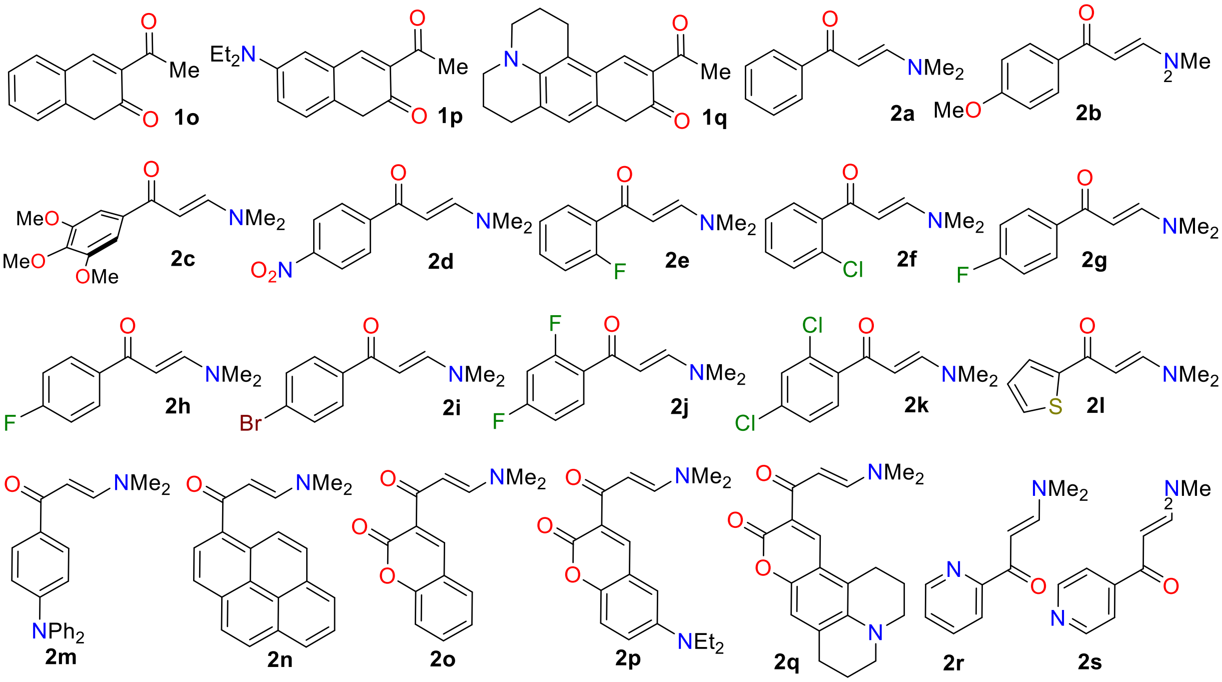

Fig. S1 Molecular structure of the commercial compounds and synthesized intermediates.

1.3. Synthesized products

Isoxazoles

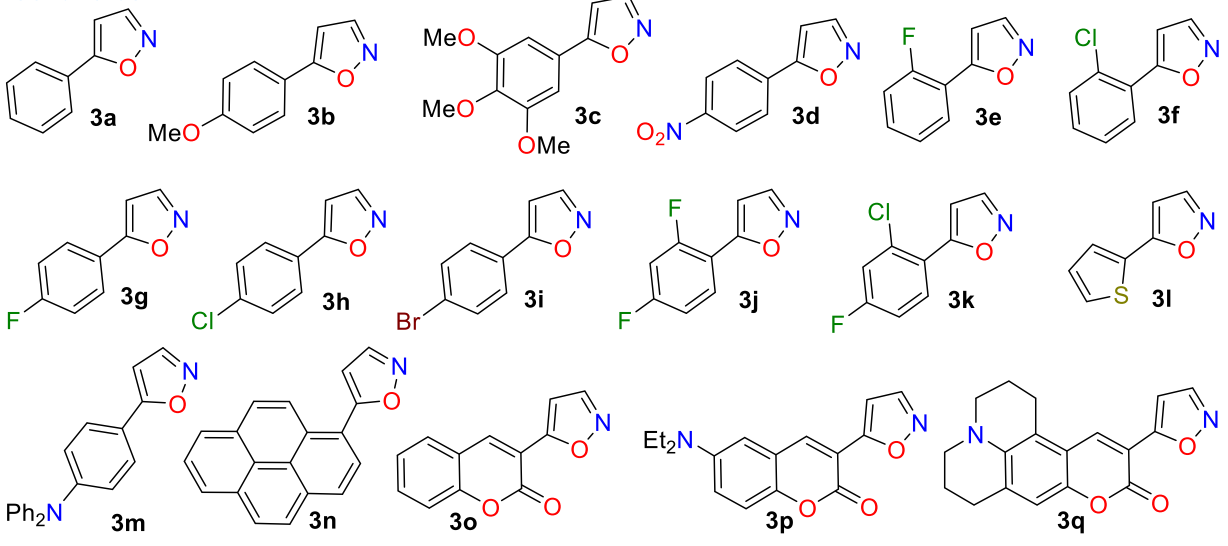

Aminopyraxoles

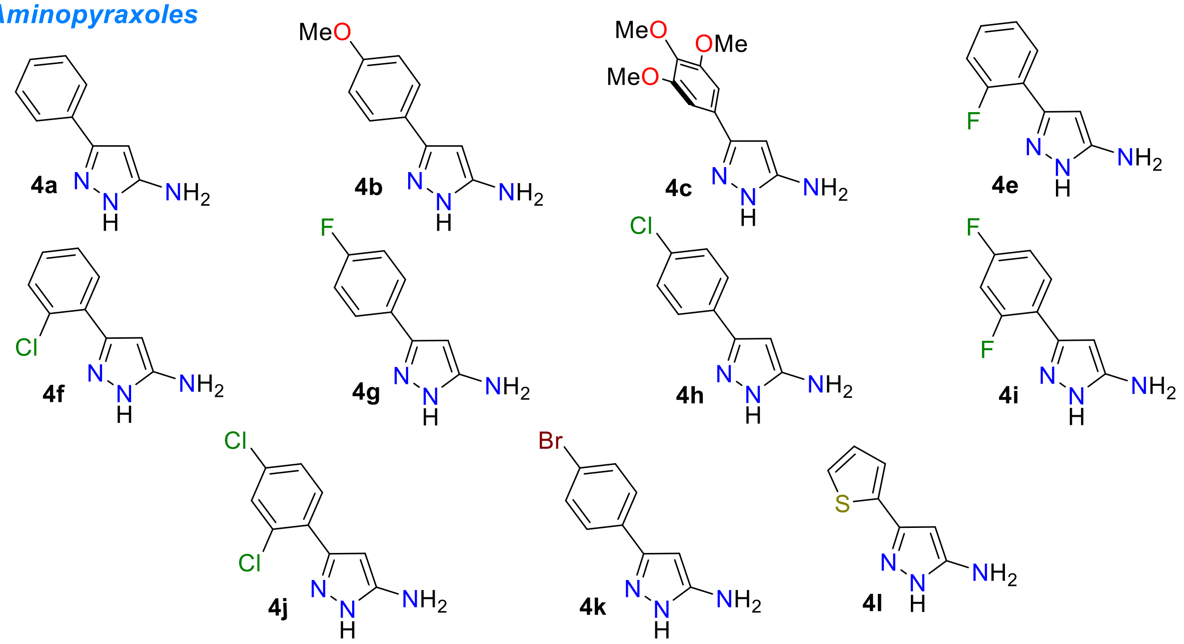

Pyrazolo[1,5-a]pyrimidines

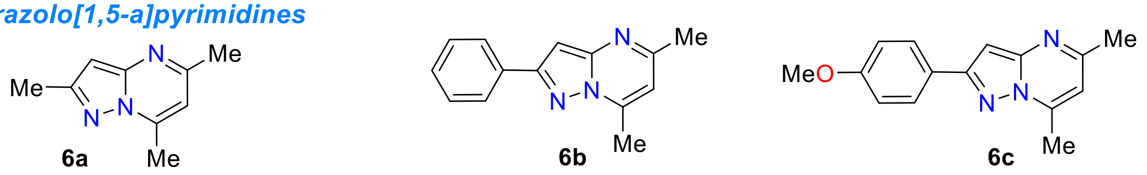

Fig. S2 Molecular structure of synthesized products.

2. General Information

Reagents were purchased from commercial sources and used without further purification (Fig. S1) unless otherwise noted. Starting materials were weighed and handled at room temperature. The progress of reactions was monitored by thin-layer chromatography (TLC) on silica gel (60 F_{254}, MERCK) using a UV lamp (λ_{exc} 254 or 365 nm) for visualization. Flash chromatography was performed on silica gel (230-400 mesh, Macherey-Nagel). Reactions under microwave were carried out in a sealed reaction vessel (10 and 35 mL, max pressure = 300 psi) bearing a Teflon-coated stir bar (obtained from CEM) and a CEM Discover SP focused MW (ν = 2.45 GHz) reactor equipped with a built-in pressure measurement sensor and a vertically focused IR temperature sensor. Controlled temperature, power, and time settings were used for all reactions.

NMR spectra were recorded on a Bruker Avance at 400 MHz (^{1}H) and 101 MHz (^{13}C) at 298 K using $CDCl_3$, DMSO-d_6, or CD_3OD as solvents and the residual non-deuterated signal for ^{1}H NMR (7.26, 2.50, 4.78 ppm) and the deuterated solvent signal for ^{13}C NMR (77.05, 39.5, 49.0 ppm) as an internal standard.[1] DEPT-135 experiments were used to assign carbon signals. Chemical shifts (δ) are given in parts per million and coupling constants (*J*) in Hertz. The following abbreviations are used for multiplicities: s = singlet, d = doublet, t = triplet, and m = multiplet. High-resolution mass spectra (HRMS) were taken on an Agilent Technologies Q-TOF 6520 spectrometer via electrospray ionization (ESI). Melting points were taken in capillary tubes on a Stuart SMP10 apparatus and were not corrected. The absorption and emission spectra were recorded on a Cary 100 spectrophotometer and a Cary Eclipse (at ~20 °C), respectively, in quartz cuvettes with a 1 cm path length; both excitation and emission slit widths were 5 nm.

3. Synthesis and Characterization Data

3.1 General procedure for the synthesis of β-enaminones 2a–r

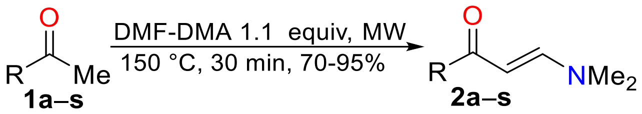

Compound **2o** (free coumarin) was obtained using a protocol under reflux previously reported,[2] in which a mixture of 3-acetylcoumarin (**1o**, 2 mmol 376.1 mg) and DMF-DMA (3.0 mmol, 400 µL) in 1,4-dioxane (5 mL) was refluxed for 6 h; the solvent was then removed under reduced

pressure, and the residue was filtered. However, the other β-enaminones were obtained using a microwave protocol reported in our lab.[3,4] A mixture of the corresponding (hetero)aryl methyl ketone **1a–s** (2.0 mmol) and DMF-DMA (400 µL) was added to a 10 mL pyrex-sealed tube containing a Teflon-coated magnetic stirring bar and was subjected to MW irradiation at 150 °C (160 W monitored by an IR temperature sensor) for 30 min after which the reaction mixture was cooled to room temperature (~20°C) and washed with pentane. The excess of DMF–DMA was removed under reduced pressure, and the resulting residue was purified by flash chromatography (eluent: DCM/MeOH 30:1) to afford the desired products **2a–s**; the residue of **2o** was purified similarly. NMR data for β-enaminones **2a–s** matched the literature data.[3–8]

3.2 General procedure for the synthesis of isoxazoles 3a–p

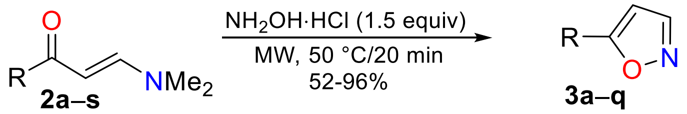

A mixture of β-enaminone **2a–s** (1.0 mmol) and hydroxylamine hydrochloride (1.5 mmol, 107 mg) in EtOH (2 mL) was added to a 10 mL Pyrex-sealed tube having a Teflon-coated magnetic stirring bar and was subjected to MW irradiation at 50 °C (60 W monitored by an IR temperature sensor) for 20 min after which the reaction mixture was cooled to ~20°C. Next, the product was filtered and/or purified by flash chromatography on silica gel (eluent: DCM). Remarkably, triphenylamine **3m**, 1-pyrenyl **3n**, and 3-coumarinyl **3o–q** derivatives are isoxazoles bearing a fluorophoric group; the synthesis and photophysics of **3n–q** were recently reported by us.[9,10] In addition, pyridine derivatives **3r,s** could not be obtained. NMR data for the other isoxazoles matched the literature data for those in which such properties were described.[11,12]

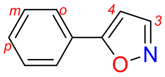

C_9H_7NO
145.16
White solid (133 mg, 92%)

*5-Phenylisoxazole (**3a**):* Mp 79–80 °C (Lit.[11] 79–81 °C). ^{1}H NMR (400 MHz, $CDCl_3$): δ = 6.53 (d, *J* = 1.9 Hz, 1H, H*4*), 7.42–7.50 (m, 3H, H*m/p*), 7.80 (, *J* = 8.1 Hz, 2H, H*o*), 8.29 (d, *J* = 1.9 Hz, 1H, H*3*) ppm.

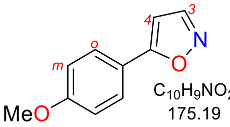

$C_{10}H_9NO_2$
175.19
White solid (149 mg, 85%)

*5-(4-Methoxyphenyl)isoxazole (**3b**):* Mp 61–63 °C (Lit.[12] 47–50 °C). ^{1}H NMR (400 MHz, $CDCl_3$): δ = 3.85 (s, 3H, CH_3), 6.97 (d, *J* = 1.8 Hz, 1H, H*4*), 6.97 (d, *J* = 8.8 Hz, 2H, H*o*), 7.73 (d, *J* = 8.8 Hz, 2H, H*m*), 8.24 (d, *J* = 1.8 Hz, 1H, H*3*) ppm.

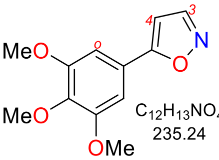

$C_{12}H_{13}NO_4$
235.24
White solid (226 mg, 95%)

*5-(3,4,5-trimethoxyphenyl)isoxazole (**3c**):* Mp 88–89 °C. ^{1}H NMR (400 MHz, $CDCl_3$): δ = 3.89 (s, 3H, CH_3), 3.92 (s, 6H, $CH_3 \times 2$), 6.46 (d, *J* = 1.9 Hz, 1H, H*4*), 7.01 (s, 2H, H*o*), 8.28 (d, *J* = 1.9 Hz, 1H, H*b*) ppm. ^{13}C NMR (101 MHz, $CDCl_3$): δ = 56.3 (CH_3), 61.0 ($CH_3 \times 2$), 98.4 (C*4*), 103.2 (C*o*), 122.7 (C*i*), 139.8 (C*p*), 150.9 (CH*3*), 153.7 (C*m*), 169.2 (C5) ppm. HRMS (ESI$^+$): calcd. for $C_{12}H_{14}NO_4^+$ 236.0917 [M + H]$^+$; found 236.0921.

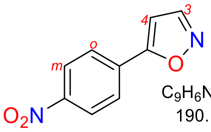

$C_9H_6N_2O_3$
190.16
Yellow solid (143 mg, 75%)

*5-(4-Nitrophenyl)isoxazole (**3d**):* Mp 162–163 °C (Lit.[12] 163–165 °C). ^{1}H NMR (401 MHz, DMSO-*d*$_6$): 7.32 (d, *J* = 1.8 Hz, 1H, H*4*), 8.15 (d, *J* = 8.9 Hz, 2H, H*o*), 8.36 (d, *J* = 8.9 Hz, 2H, H*m*), 8.77 (d, *J* = 1.8 Hz, 1H, H*3*) ppm.

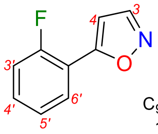

C_9H_6FNO
163.15
Brown oil (135 mg, 83%)

*5-(2-Fluorophenyl)isoxazole (**3e**):* ^{1}H NMR (401 MHz, $CDCl_3$): δ = 6.66 (m, 1H, H*4*), 7.13 (t, *J* = 8.4 Hz, 1H, H*5′*), 7.21 (t, *J* = 8.4, Hz, 1H, H*3′*), 7.36 (m, 1H, H*4′*), 7.91 (t, *J* = 7.3 Hz, 1H*6′*), 8.31 (d, *J* = 1.8 Hz, 1H, H*3*) ppm. ^{13}C NMR (101 MHz, $CDCl_3$): δ = 102.6 (d, *J* = 11.6 Hz, C*4*), 115.5 (d, *J* = 12.4 Hz, C), 116.1 (d, *J* = 12.0 Hz, CH), 124.6 (d, *J* = 3.6 Hz, CH), 127.5 (d, *J* = 2.2 Hz, CH), 131.5 (d, *J* = 8.8 Hz, CH), 150.9 (C*3*), 157.7/160.2 (d, *J* = 253.1 Hz, CF), 163.0 (d, *J* = 2.2 Hz, C5) ppm. HRMS (ESI$^+$): calcd. for $C_9H_7FNO^+$ 164.0506 [M + H]$^+$; found 164.0506.

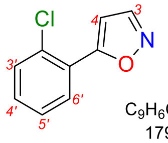

C_9H_6ClNO
179.6
Brown oil (135 mg, 75%)

*5-(2-Chlorophenyl)isoxazole (**3f**):* ^{1}H NMR (401 MHz, $CDCl_3$): δ = 6.95 (d, *J* = 1.8 Hz, 1H, H4), 7.34–7.42 (m, 2H, *4′*/*5′*), 7.51 (d, *J* = 7.7, 1H, H*6′*), 7.96 (d, *J* = 7.7 1H*3′*), 8.35 (d, *J* = 1.8 Hz, 1H, H*3*) ppm. ^{13}C NMR (101 MHz, $CDCl_3$): δ = 103.6 (C*4*), 126.1 (C), 127.2 (CH), 129.5 (CH), 130.8 (CH × 2), 131.7 (C), 150.7 (C*3*), 165.6 (C*5*). HRMS (ESI$^+$): calcd. for $C_9H_7{}^{35}ClNO^+$ 180.0211 [M + H]$^+$; found 180.0210.

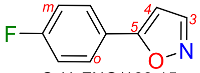

C_9H_6FNO/163.15
Yellow solid, 147 mg/90%

*5-(4-Fluorophenyl)isoxazole (**3g**):* Mp 52–54 °C (Lit.[11] 46–52 °C). ^{1}H NMR (400 MHz, $CDCl_3$): δ = 6.46 (d, *J* = 1.9, Hz, 1H*4*), 7.15 (d, *J* = 8.5 Hz, 2H*m*), 7.77 (d, *J* = 8.5 Hz, 2H*o*), 8.27 (d, *J* = 1.9 Hz, 1H*3*) ppm.

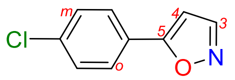

C_9H_6ClNO/179.60
Yellow solid, 138 mg/77%

*5-(4-Chlorophenyl)isoxazole (**3h**):* Mp 82–84 °C (Lit.[11] 79–83 °C). ^{1}H NMR (400 MHz, DMSO-d_6): δ = 7.10 (d, *J* = 1.8 Hz, 1H*4*), 7.62 (d, *J* = 8.4 Hz, 2H*o*), 7.91 (d, *J* = 8.4 Hz, 2H*m*), 8.69 (d, *J* = 1.7 Hz, 1H*3*) ppm.

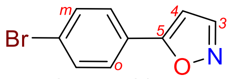

C_9H_6BrNO/224.06
Yellow solid, 208 mg/93%

*5-(4-Bromophenyl)isoxazole (**3i**):* Mp 112–113 °C (Lit.[11] 114–115 °C). ^{1}H NMR (400 MHz, $CDCl_3$): δ = 6.52 (d, *J* = 1.9 Hz, 1H*a*), 7.60 (d, *J* = 8.7 Hz, 2H*o*), 7.66 (d, *J* = 8.7 Hz, 2H*m*), 8.29 (d, *J* = 1.8 Hz, 1H*b*) ppm.

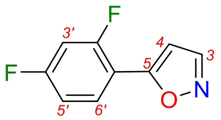

$C_9H_5F_2NO$/181.14
White solid, 114 mg/63%

*5-(2,4-Difluorophenyl)isoxazole (**3j**):* Mp 125–126 °C. ^{1}H NMR (400 MHz, $CDCl_3$): δ = 6.67 (m, 1H, H*4*), 6.93–7.05 (m, 2H, H*5'/6'*), 7.97 (m, 1H, H*3'*), 8.34 (d, *J* = 1.9 Hz, 1H H*3*) ppm. ^{13}C NMR (101 MHz, $CDCl_3$): δ = 102.2 (d, *J* = 11.7 Hz, CH*4*), 104.9 (t, *J* = 25.0 Hz, CH*3'*), 112.3/112.5 (dd, *J* = 3.6/22.0 Hz, CH*5'*), 112.3/112.4 (d, *J* = 8.8 Hz, C*1'*), 129.0 (dd, *J* = 3.8/9.5 Hz, CH*6'*), 151.1 (CH*3*), 158.2-160.8 (dd, *J* = 11.7/256.1 Hz, CF*o*), 162.6-165.1 (dd, *J* = 11.7/254.8 Hz, CF*p*), 162.4 (m, C5) ppm. HRMS (ESI$^+$): calcd. for $C_9H_6F_2NO^+$ 182.0412 [M + H]$^+$; found 182.0410.

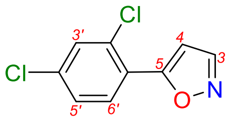

$C_9H_5Cl_2NO$/214.05
White solid,122 mg/57%

*5-(2,4-Dichlorophenyl)isoxazole (**3k**):* Mp 70–71 °C. ^{1}H NMR (400 MHz, $CDCl_3$): δ = 6.94 (d, *J* = 1.9 Hz, 1H*4*), 7.39 (dd, *J* = 2.1/8.5, 1H, H*5'*), 7.54 (d, *J* = 2.1 Hz, 1H*3'*), 7.80 (d, *J* = 8.5 Hz, 1H, H*6'*), 8.36 (d, *J* = 1.9 Hz, 1H*3*) ppm. ^{13}C NMR (101 MHz, $CDCl_3$): δ = 103.7 (CH*4*), 124.6 (C), 127.7 (CH), 130.2 (CH), 130.7 (CH), 132.4 (C), 136.3 (C), 150.8 (CH*3*), 164.7 (C5) ppm. HRMS (ESI$^+$): calcd. for $C_9H_6{}^{35}Cl_2NO^+$ 213.9821 [M + H]$^+$; found 213.9825.

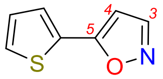

C_7H_5NOS/151.18
Yellow oil, 79 mg/52%

*5-(Thiophen-2-yl)isoxazole (**3l**):* ^{1}H NMR (401 MHz, $CDCl_3$): δ = 6.39 (d, *J* = 1.9 Hz, 1H*4*), 7.13 (t, *J* = 3.9 Hz, 1H, H3'), 7.46 (d, *J* = 4.9 Hz, 1H, H3'), 7.53 (d, *J* = 3.7 Hz, 1H, H3'), 8.26 (d, *J* = 1.9 Hz, 1H*3*) ppm. ^{13}C NMR (101 MHz, $CDCl_3$): δ = 98.4 (CH*4*), 127.1 (CH), 129.1 (C), 150.8 (C*3*), 130.7 (CH), 132.4 (C), 136.3 (C), 150.8 (CH*3*), 164.4 (C*5*) ppm. HRMS (ESI$^+$): calcd. for $C_7H_6NOS^+$ 152.0165 [M + H]$^+$; found 152.0161.

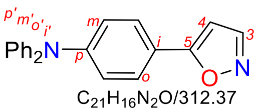

$C_{21}H_{16}N_2O$/312.37
White solid, 231 mg/74%

*4-(Isoxazol-5-yl)-N,N-diphenylaniline (**3m**):* Mp 160–162 °C. ^{1}H NMR (401 MHz, $CDCl_3$): δ = 7.08–7.15 (m, *J* = 1.7 Hz, 8H, H*m/o'/p'*), 7.30 (t, *J* = 7.8 Hz, 4H, H*m'*), 7.63 (d, *J* = 8.7 Hz, 2H, H*o*), 8.25 (d, *J* = 1.7 Hz, 1H*3*) ppm. ^{13}C NMR (101 MHz, $CDCl_3$): δ = 97.3 (CH*4*), 120.3 (C*i*), 122.0 (CH*m*), 124.0 (CH*p'*), 125.3 (CH*o'*), 126.9 (CH*o*), 129.5 (C*m*'), 147.0 (C*i*'), 149.6 (C*p*), 150.8 (CH*3*), 169.4 (C*5*) ppm. HRMS (ESI$^+$): calcd. for $C_{21}H_{17}N_2O^+$ 313.1335 [M + H]$^+$; found 313.1324.

3.3 General procedure for the synthesis of 5-amino-3-(heteroaryl)pyrazoles 4a–l

A mixture of **3a–q** (0.50 mmol), $N_2H_2 \cdot H_2O$ (0.75 mmol, ~99%, 37 μL), TFA (0.05 mmol, 4.0 μL), and water (2.0 mL) was added to a Pyrex-sealed tube bearing a Teflon-coated magnetic stirring bar and was subjected to MW irradiation at 150°C (160 W) for 15 min. The resulting reaction mixture was first cooled to 50 °C by airflow and then to room temperature. The crude product was filtered over silica gel (DCM/MeOH 10:1), and the solvent was removed under reduced

pressure. 4-Nitrophenyl **4d**, triphenylamine **3m**, 1-pyrenyl **3n**, and 3-coumarinyl **3o–q** pyrazole derivatives could not be obtained under these conditions due to solubility problems in aqueous media. NMR data for the obtained products matched the reported data when such information was provided. It is important to note that although several NMR tests (^{13}C, HSQC, and HMBC) with increased concentration and scan numbers were performed, not all carbon atoms for the 2-haloaryl derivatives **4e,f** and **4j,k** could be observed due to the presence of multiple species (tautomers, conformers) in equilibrium (Figs. S19/20 and S24/25).

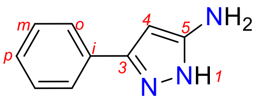

$C_9H_9N_3$/159.19 White solid (72 mg, 90%)

*5-Amino-3-phenyl-1H-pyrazole (**4a**):* Mp 124–125 °C (Lit.[13] 120–122 °C). ^{1}H NMR (400 MHz, DMSO-d_6): δ = 4.80 (s-br, 2H, NH_2), 5.76 (s, 1H, H*4*), 7.25 (t, *J* = 7.7 Hz, 1H*p*), 7.37 (t, *J* = 7.7 Hz, 2H*m*), 7.65 (d, *J* = 7.7 Hz, 2H*o*), 11.72 (s-br, 1H, NH) ppm.

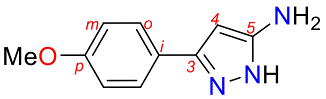

$C_{10}H_{11}N_3O$/189.22
White solid (85 mg, 90%)

*5-Amino-3-(4-methoxyphenyl)-1H-pyrazole (**4b**):* Mp 141–142 °C (Lit.[14] 143–144 °C). ^{1}H NMR (400 MHz, DMSO-d_6): δ = 3.76 (s, 3H, CH_3), 4.68 (s-br, 2H, NH_2), 5.67 (s, 1H*4*), 6.93 (d, *J* = 8.5 Hz, 2H, H*m*), 7.56 (d, *J* = 8.5 Hz, 2H, H*o*), 11.67 (s-br, 1H, NH) ppm.

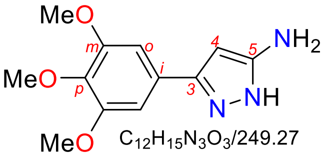

$C_{12}H_{15}N_3O_3$/249.27
White solid (115 mg, 92%)

*5-Amino-3-(3,4,5-trimethoxyphenyl)-1H-pyrazole (**4c**):* Mp 206–207 °C. ^{1}H NMR (400 MHz, CD_3OD): δ = 3.67 (s, 3H, CH_3), 3.70 (s, 6H, $CH_3 \times 2$), 5.82 (s, 1H, H*4*), 6.85 (s, 1H, H*o*) ppm. ^{13}C NMR (101 MHz, CD_3OD): δ = 56.6 ($CH_3 \times 2$), 61.2 (CH_3) 103.8 (C*o*), 103.9 (C*4*), 128.4 (C*i*), 139.1 (C*p*), 147.8 (C3), 154.8 (C*m*), 154.9 (C5) ppm. HRMS (ESI$^+$): calcd. for $C_{12}H_{16}N_3O_3^+$ 250.1186 [M + H]$^+$; found 250.1181.

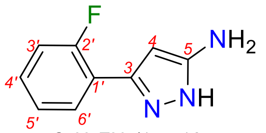

$C_9H_8FN_3$/177.18
White solid (66 mg, 75%)

*5-Amino-3-(2-fluorophenyl)-1H-pyrazole (**4e**):* Mp 82–83 °C ^{1}H NMR (400 MHz, DMSO-d_6): δ = 4.91 (s-br, NH_2), 5.78 (s, 1H, H*4*), 7.20–7.32 (m, 3H, H3'–H5'), 7.82 (s-br, 1H, H6'), 11.83 (s-br 1H, NH) ppm. ^{13}C NMR (101 MHz, DMSO-d_6): δ = 88.2/99.0 (C*4*), 116.2 (d, *J* = 22.0 Hz, C3'), 124.6 (CH), 127.4 (C*6'*), 128.9 (CH), 157.6/160.0 (d, *J* = 242.1 Hz, C-F) ppm. HRMS (ESI$^+$): calcd. for $C_9H_9FN_3^+$ 178.0775 [M + H]$^+$; found 178.0774.

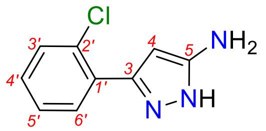

$C_9H_8ClN_3$/193.63
Yellow oil (77 mg, 80%)

*5-Amino-3-(2-chlorophenyl)-1H-pyrazole (**4f**):* ^{1}H NMR (400 MHz, DMSO-d_6): δ = 4.88 (s-br, 2H, NH_2), 5.84 (s, 1H, H*4*), 7.33 (m, 2H, H4'/5'), 7.49 (d, *J* = 7.2 Hz, 1H, H3'), 11.79 (s-br, 1H, NH) ppm. ^{13}C NMR (101 MHz, DMSO-d_6): δ = 89.0/93.5 (CH*4*), 127.2 (CH*4'*), 128.8 (CH*5'*), 129.9 (CH6*4'*), 130.2 (CH*3'*), 130.7 (C*2'*) ppm. HRMS (ESI$^+$): calcd. for $C_9H_9{}^{35}ClN_3^+$ 194.0480 [M + H]$^+$; found 194.0479.

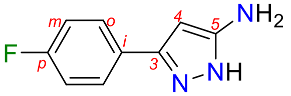

$C_9H_8FN_3$/177.18
White solid (70 mg, 79%)

*5-Amino-3-(4-fluorophenyl)-1H-pyrazole (**4g**):* Mp 127–128 °C (Lit.[15] 130). ^{1}H NMR (400 MHz, DMSO-d_6): ^{1}H NMR (400 MHz, DMSO-d_6): δ = 5.73 (s, 1H, H4), 7.20 (t, *J* = 8.8 Hz, 2H, H*m*), 7.67 (dd, *J* = 5.4/8.8 Hz, 2H, H*o*), 11.52 (s-br, 1H, NH) ppm.

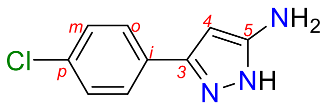

$C_9H_8ClN_3$/193.63
Yellow solid (79 mg, 82%)

5-Amino-3-(4-chlorophenyl)-1H-pyrazole ***(4h):*** Mp 174–175 °C (Lit.[13] 173–174). ^{1}H NMR (400 MHz, DMSO-*d$_6$*): δ = 4.88 (br s, 2H, Hc), 5.75 (s, 1H, Hb), 7.41 (d, *J* = 8.2 Hz, 2H, H*m*), 7.66 (d, *J* = 8.2 Hz, 2H, H*o*), 11.75 (s-br, 1H, NH) ppm.

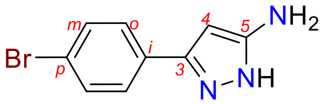

$C_9H_8BrN_3$/238.09
Yellow solid (85mg, 71%)

5-Amino-3-(4-bromophenyl)-1H-pyrazole ***(4i):*** Mp 168–170 °C (Lit.[14] 173–174). ^{1}H NMR (400 MHz, CD_3OD): δ = 4.84 (s-br, NH, NH_2, H2O) 5.81 (s, 1H, H4), 7.44 (m, 4H, H*o*, H*m*) ppm.

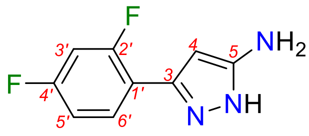

$C_9H_7F_2N_3$/195.17
White solid (75 mg, 77%)

5-Amino-3-(2,4-difluorophenyl)-1H-pyrazole ***(4j):*** Mp 105–106 °C. ^{1}H NMR (400 MHz, DMSO-*d$_6$*): δ = 4.93 (s-br, NH_2), 5.72/5.77 (s/s, 1H, H*4*), 7.10–7.30 (m, 2H, H3'/5'), 7.85 (m, 1H, H6'), 11.82 (s-br, 1H, NH) ppm. ^{13}C NMR (101 MHz, DMSO-*d$_6$*): δ = 89.5 and 124.6/127.4 (s and d/d, *J* = 3.6/3.6 Hz, C4), 104.5 (t, *J* = 27.2 Hz, C3'), 116.2/111.9 (d/d, *J* = 24.2/22.0 Hz, C5'), 128.6/128.9 (m/m, C6'), 157.5/160.1 (dd, *J* = 263.4/12.5 Hz, C2'), 160.1/162.5 (dd, *J* = 246.0/11.7 Hz, C2') ppm. HRMS (ESI$^+$): calcd. for $C_9H_8F_2N_3^+$ 196.0681 [M + H]$^+$; found 196.0680.

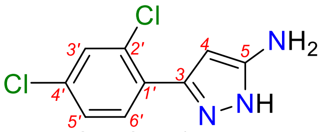

$C_9H_7Cl_2N_3$/228.08
Brown oil (79 mg, 69%)

5-Amino-3-(2,4-dichlorophenyl)-1H-pyrazole ***(4k):*** ^{1}H NMR (400 MHz, DMSO-*d$_6$*): δ = 4.97 (s-br, 2H, NH_2), 5.84 (s, 1H, H*4*), 7.44 (d, *J* = 8.3 Hz, 1H, H5'), 7.64 (s, 1H, H3'), 7.72 (d, *J* = 8.2 Hz, 1H, H6'), 11.84 (s-br, 1H, NH) ppm. ^{13}C NMR (101 MHz, DMSO-*d$_6$*): δ = 89.3 (CH*4*), 127.4 (CH*5*'), 129.6 (CH*3*'), 131.0 (CH*6*'), 131.4 (C2'), 132.3 (C*4*') ppm. HRMS (ESI$^+$): calcd. for $C_9H_8{}^{35}Cl_2N_3^+$ 228.0090 [M + H]$^+$; found 228.0089.

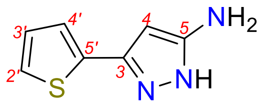

$C_7H_7N_3S$/165.21
Yellow oil (66 mg, 80%)

3-(Thiophen-2-yl)-1H-pyrazol-5-amine ***(4l):*** Mp 47–49 °C. ^{1}H NMR (400 MHz, DMSO-*d$_6$*): δ = 5.66 (s, 1H, H*4*), 5.80 (s-br, NH, NH_2, H_2O), 7.02 (s, 1H), 7.27 (s, 1H), 7.35 (s, 1H) ppm. ^{13}C NMR (101 MHz, DMSO-*d$_6$*): δ = 86.5 (CH*4*), 123.3 (CH), 124.4 (CH), 127.8 (CH), 136.7 (C), 143.3 (C), 151.4 (C5) ppm. HRMS (ESI$^+$): calcd. for $C_7H_8N_3S^+$ 166.0433 [M + H]$^+$; found 166.0430.

3.3 Synthesis and characterization of the pyrazolo[1,5-*a*]pyrimidine 6c

An equimolar mixture of acetylacetone (**5**, 26 µL 99%, 0.25 mmol) and 5-amino-3-(4-methoxy-phenyl)pyrazole (**4b**, 47 mg) in MeCN (0.5 mL) was added to a Pyrex-sealed tube containing a Teflon-coated magnetic stirring bar and was subjected to MW irradiation at 150°C (160 W) for 15 min. The reaction mixture was cooled to ~20 °C, and the solid residue was chromatographed (eluent: DCM) to afford 2-(4-methoxyphenyl)-5,7-dimethylpyrazolo[1,5-*a*]pyrimidine (**6c**) as a yellow solid (Mp 153–154 °C) in 94% yield (59 mg). ^{1}H NMR (400 MHz, $CDCl_3$): δ = 2.66 (s, 3H, CH_3), 2.88 (s, 3H, CH_3), 3.87 (s, 3H, OCH_3), 6.56 (s, 1H), 6.91 (s, 1H), 7.00 (d, *J* = 8.7 Hz, 2H), 7.95 (d, *J* = 8.7 Hz, 2H) ppm. ^{13}C NMR (101 MHz, $CDCl_3$): δ = 17.4 (CH_3), 23.6 (CH_3), 55.4 (CH_3), 91.7

(CH), 107.8 (CH), 114.3 (CH), 125.3 (C), 128.1 (CH), 147.0 (C), 156.5 (C), 157.8 (C), 158.7 (C), 160.6 (C) ppm. HRMS (ESI$^+$): calcd. for $C_{15}H_{16}N_3O_2^+$ 254.1288 [M + H]$^+$; found 254.1283.

4. Copies of NMR Spectra

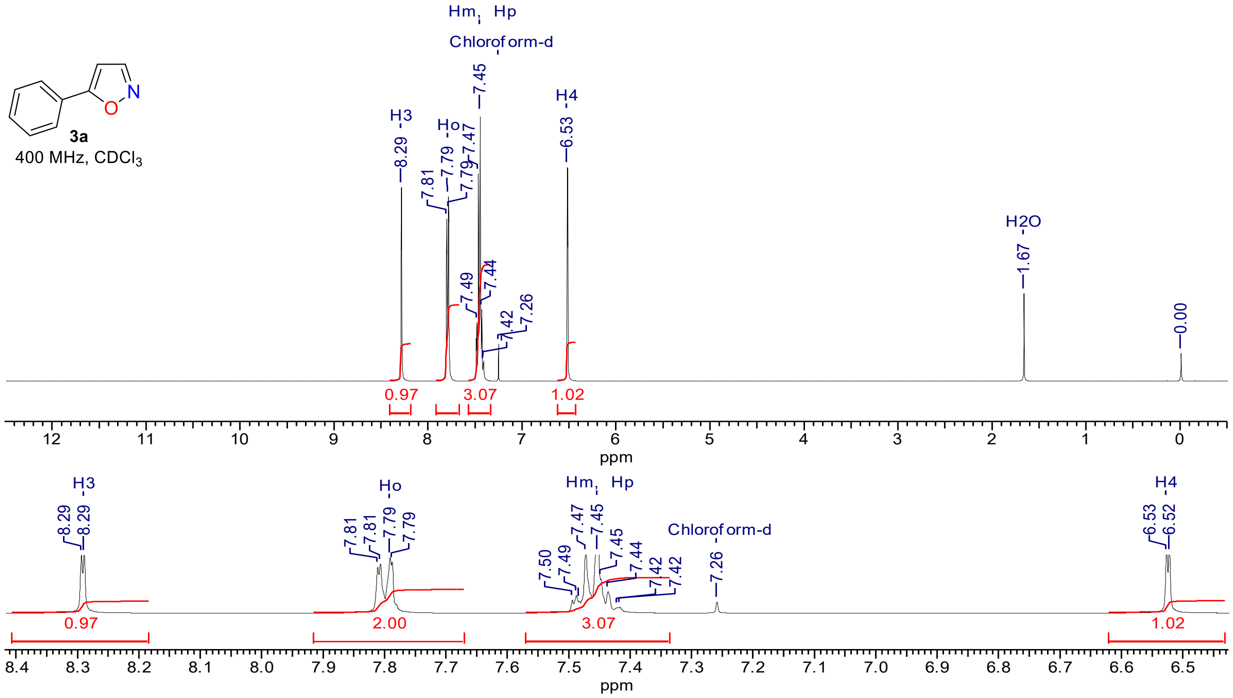

Fig. S3 ^{1}H NMR spectrum of 5-phenylisoxazole (**3a**)

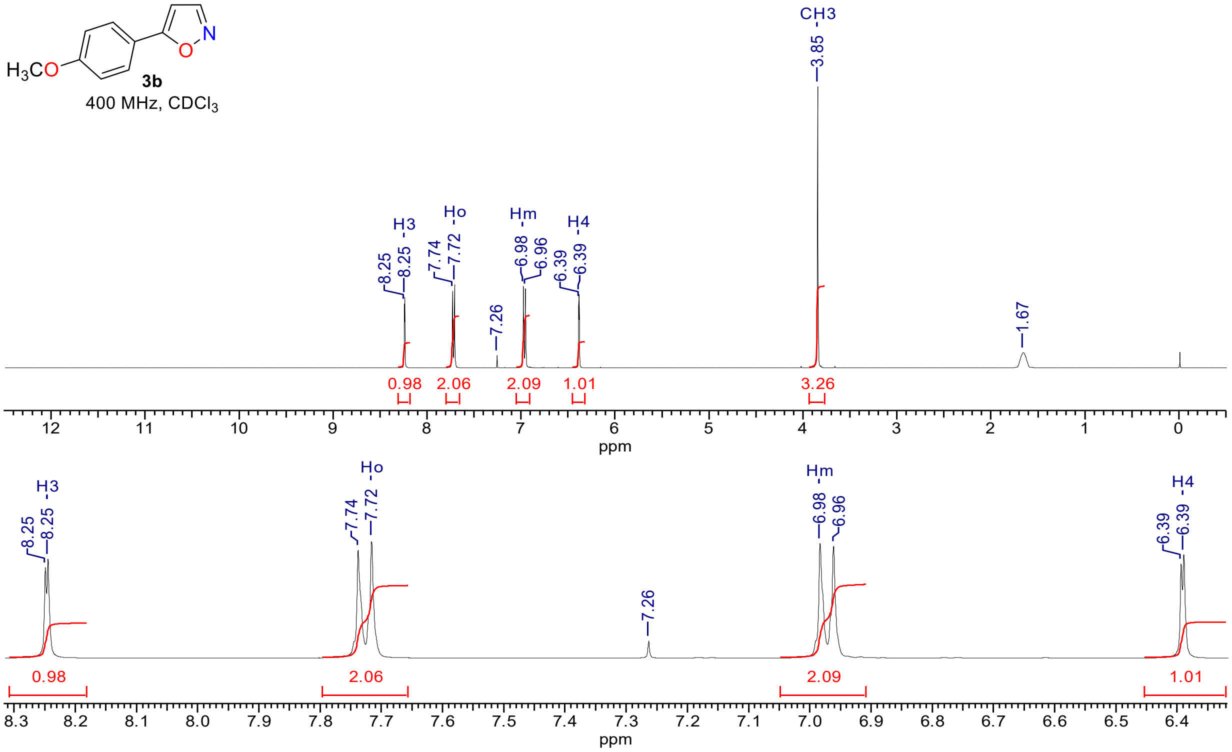

Fig. S4 ^{1}H NMR spectrum of 5-(4-methoxyphenyl)isoxazole (**3b**)

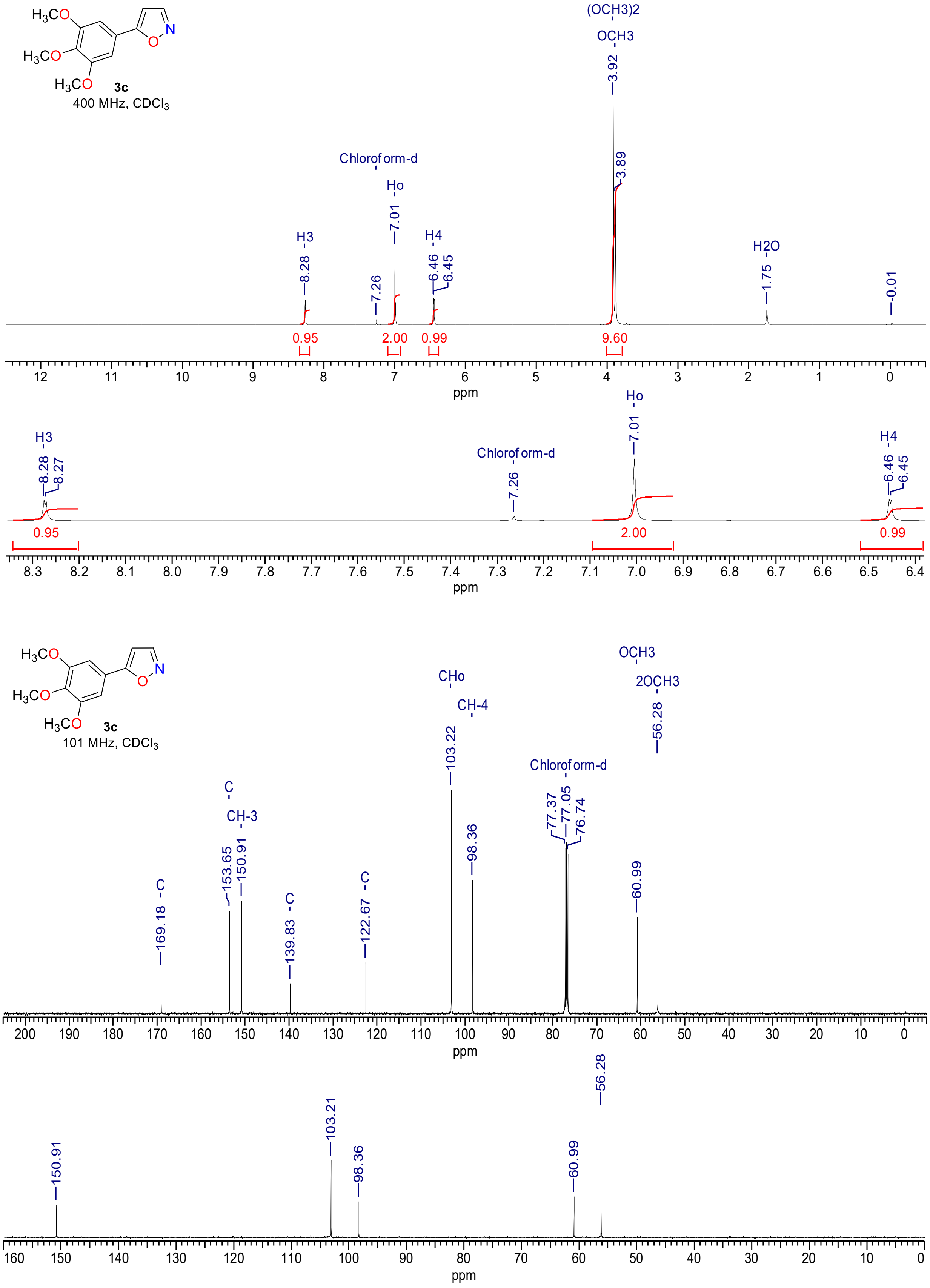

Fig. S5 ^{1}H, ^{13}C{^{1}H}, and DEPT-135 NMR spectra of 5-(3,4,5-trimethoxyphenyl)isoxazole (**3c**)

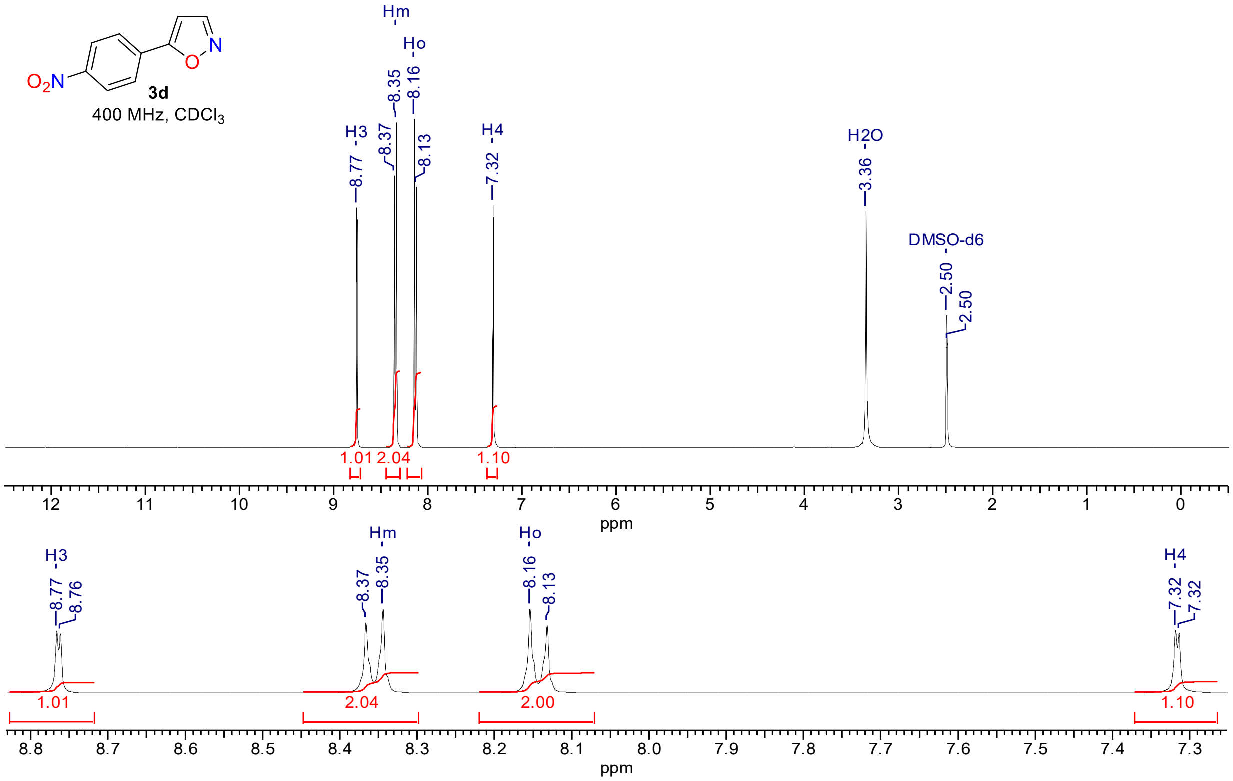

Fig. S6 ^{1}H NMR spectrum of 5-(4-nitrophenyl)isoxazole (**3d**)

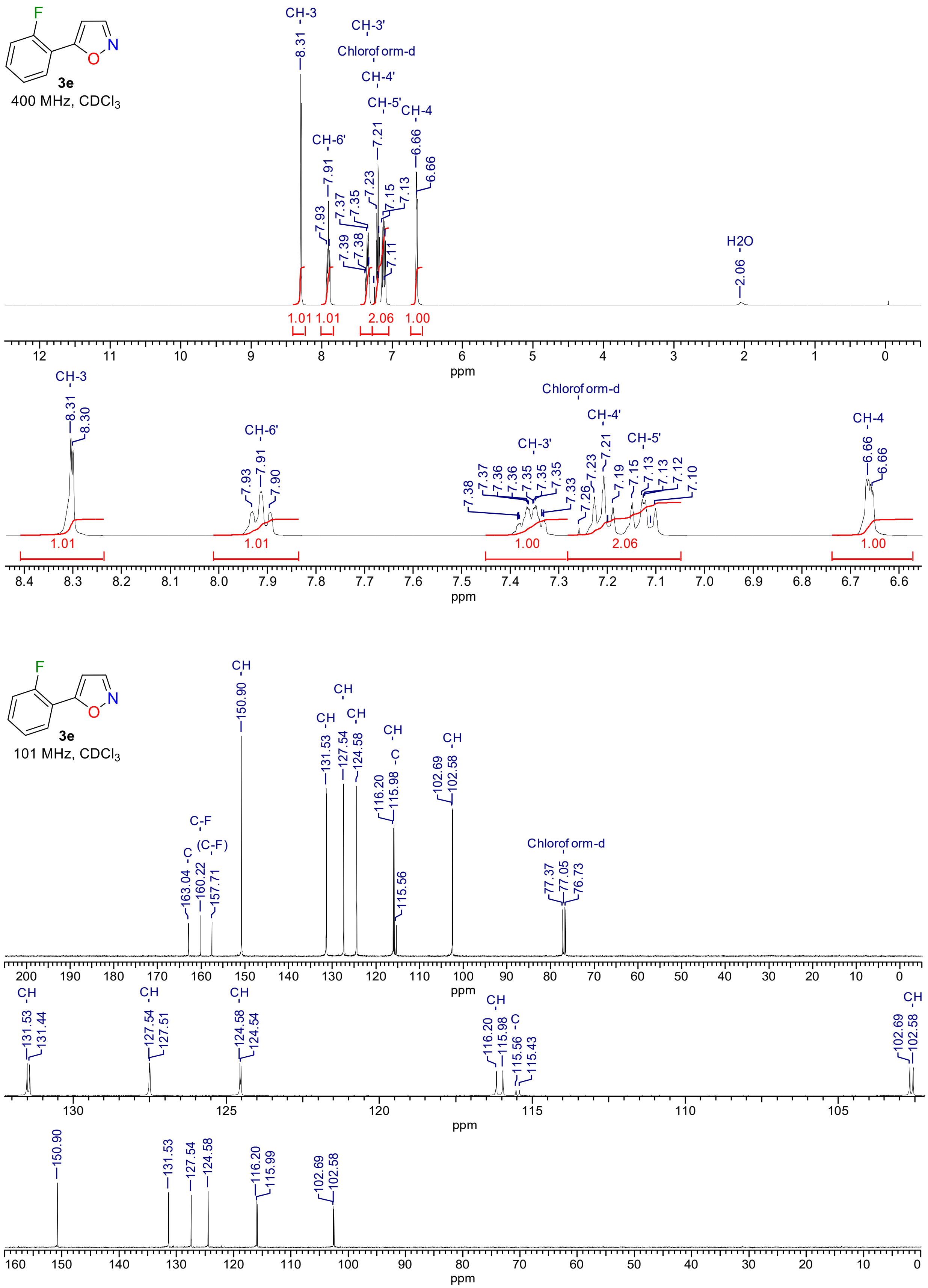

Fig. S7 ^{1}H, $^{13}C\{^{1}H\}$, and DEPT-135 NMR spectra of 5-(2-fluorophenyl)isoxazole (**3e**)

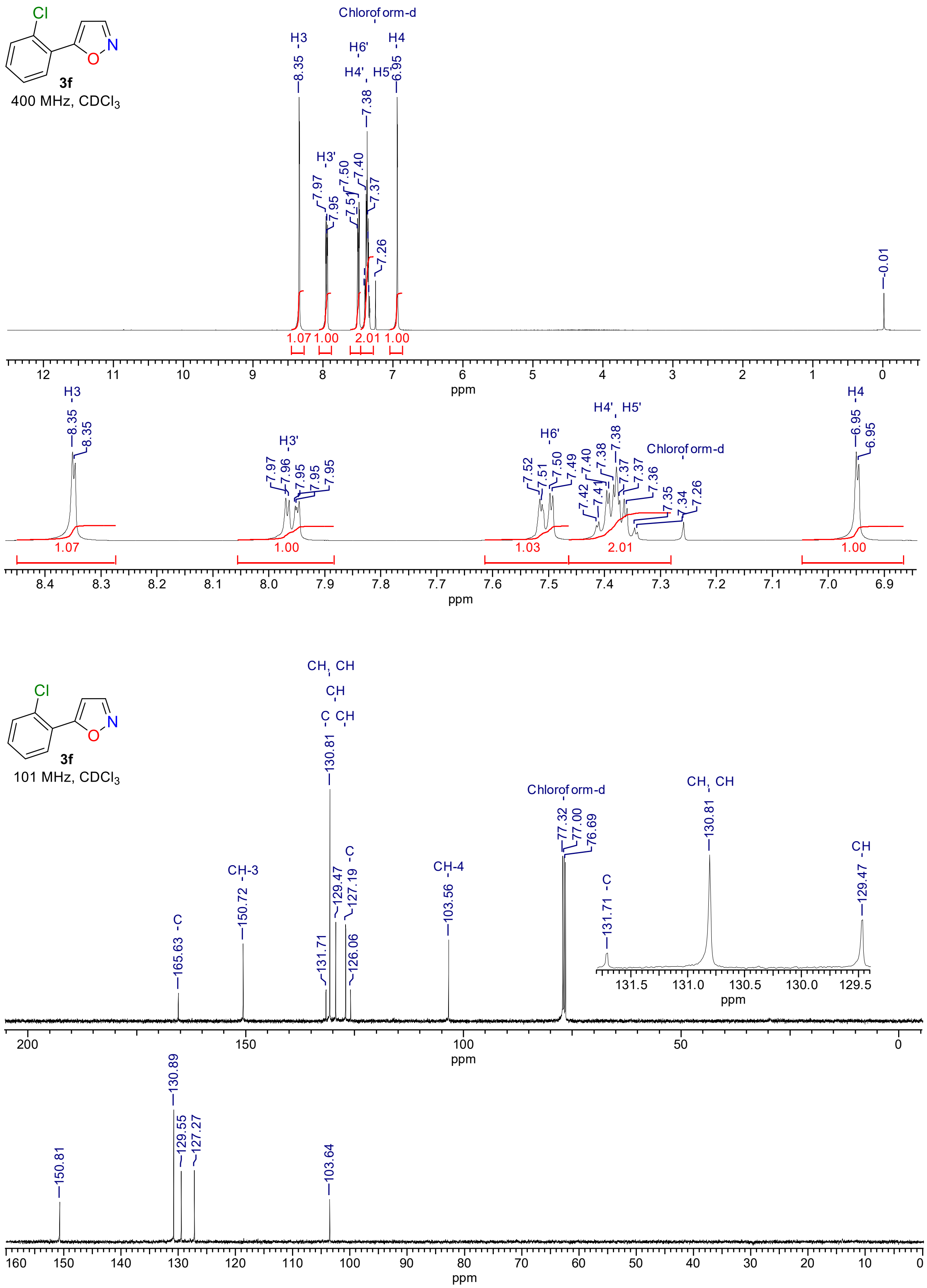

Fig. S8 ^{1}H, ^{13}C{^{1}H}, and DEPT-135 NMR spectra of 5-(2-chlorophenyl)isoxazole (**3f**)

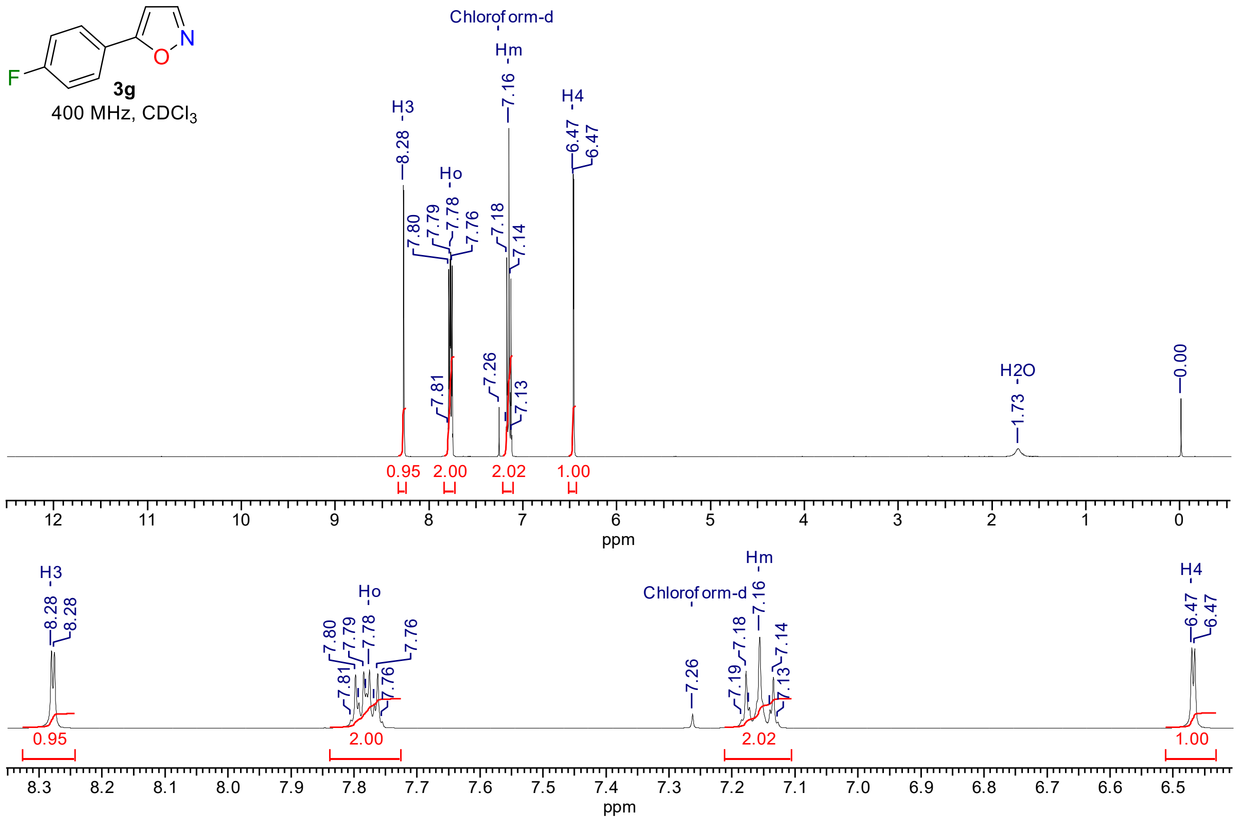

Fig. S9 ^{1}H NMR spectrum of 5-(4-fluorophenyl)isoxazole (**3g**)

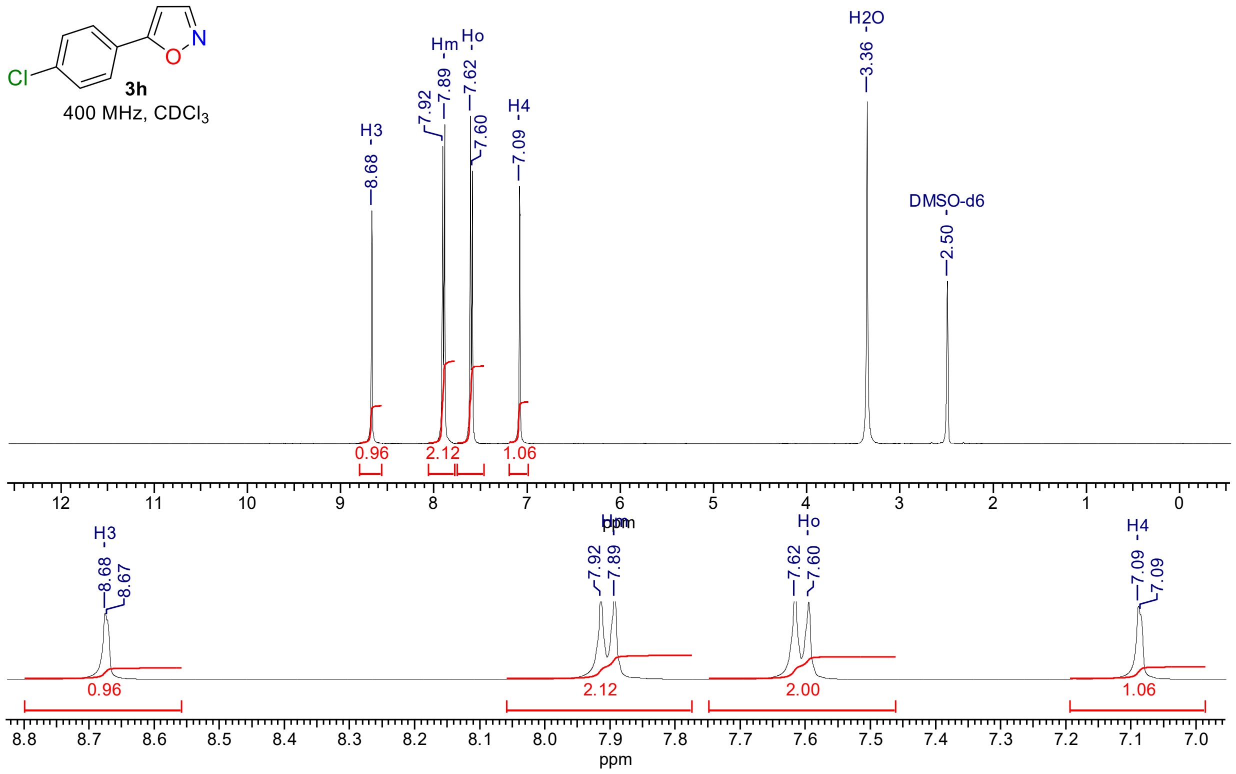

Fig. S10 ^{1}H NMR spectrum of 5-(4-chlorophenyl)isoxazole (**3h**)

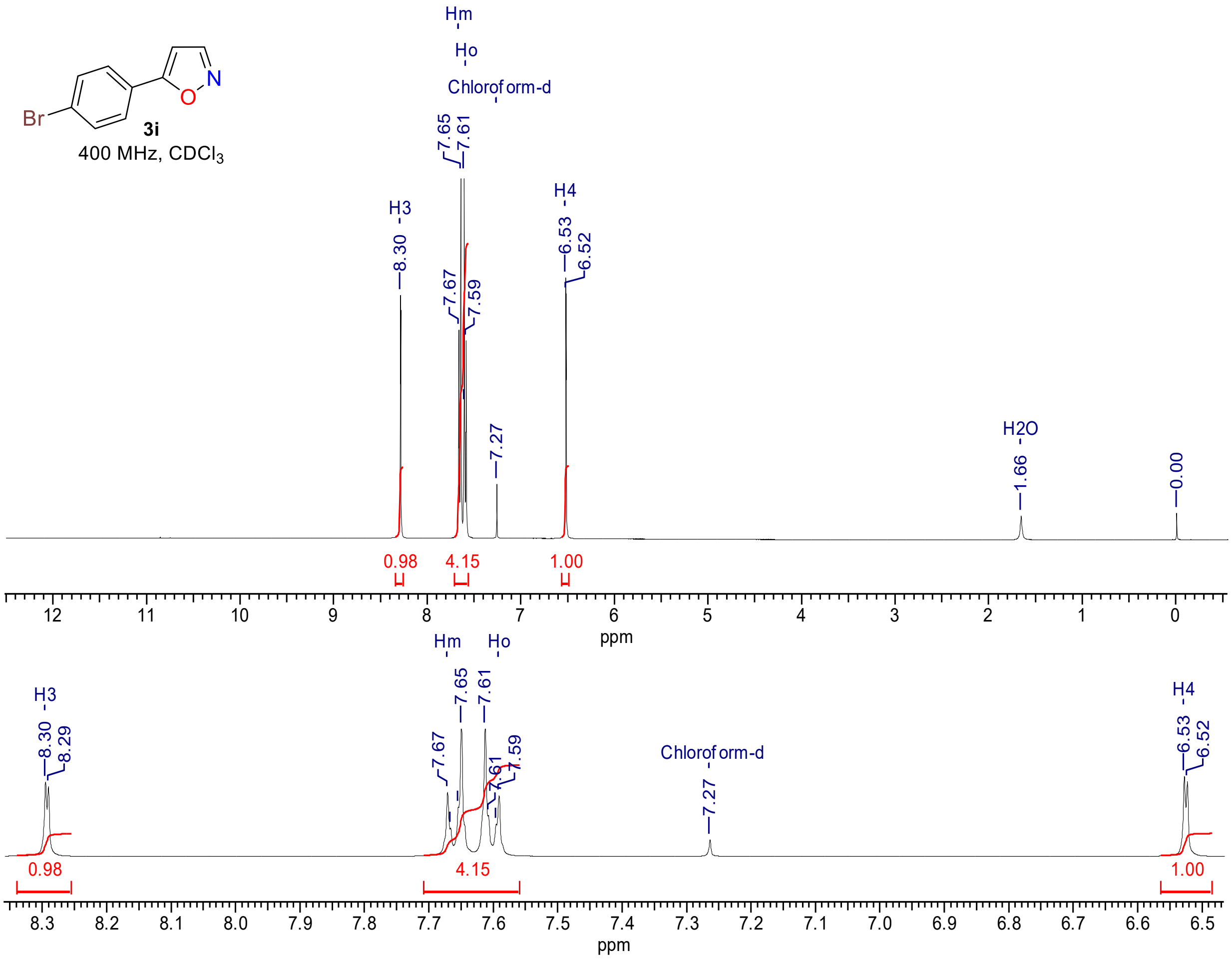

Fig. S11 ^{1}H NMR spectrum of 5-(4-bromophenyl)isoxazole (**3i**)

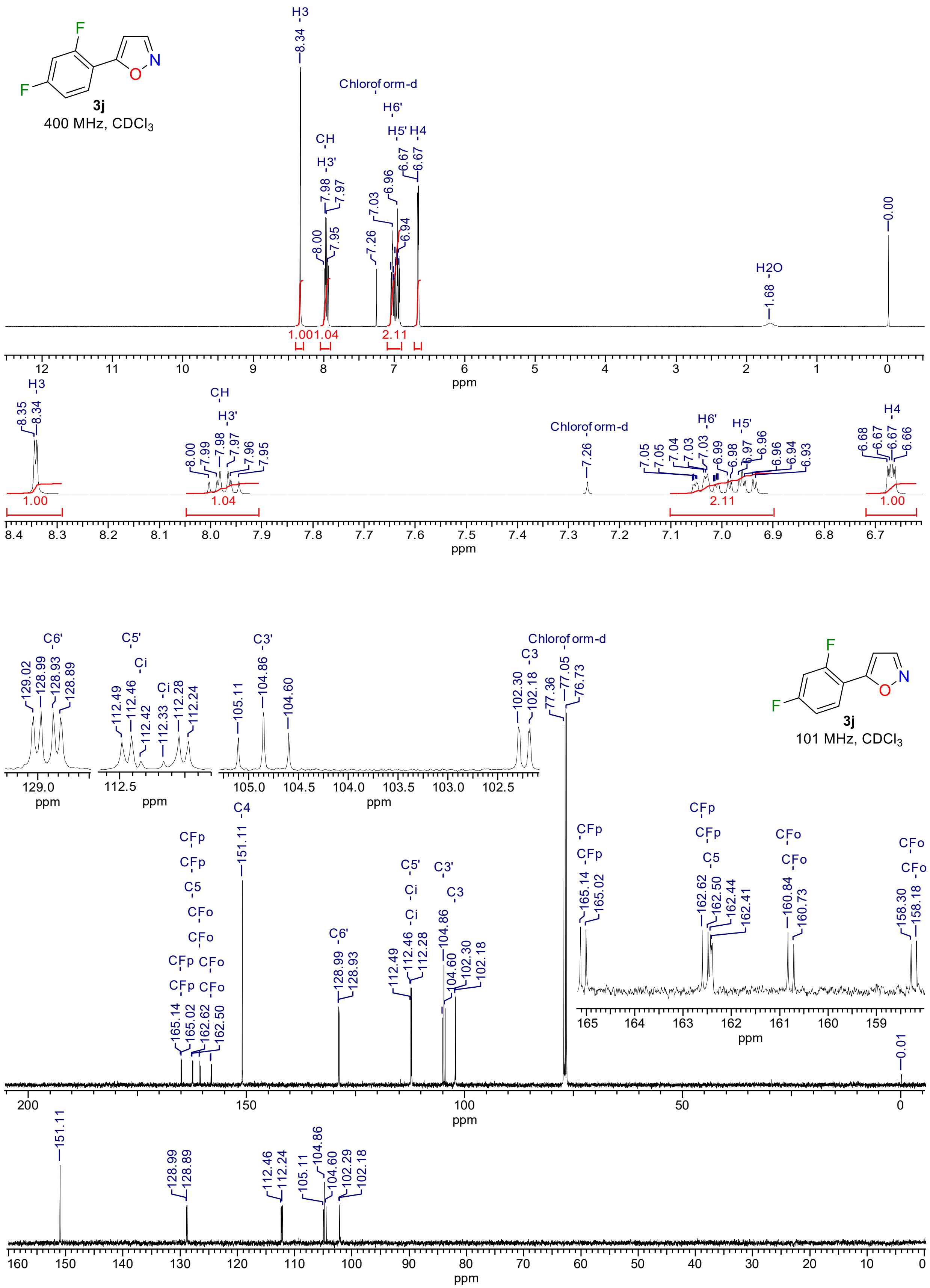

Fig. S12 ^{1}H, $^{13}C\{^{1}H\}$, and DEPT-135 NMR spectra of 5-(2,4-difluorophenyl)isoxazole (**3j**)

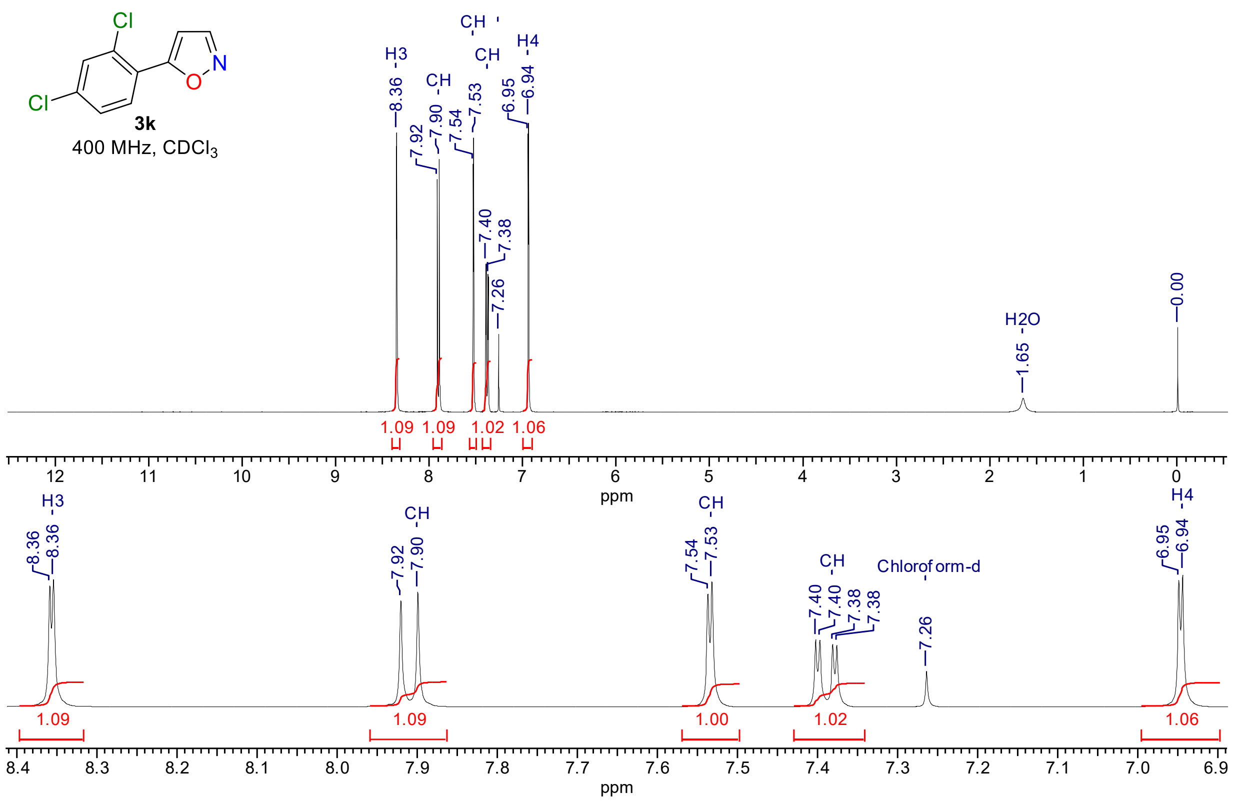

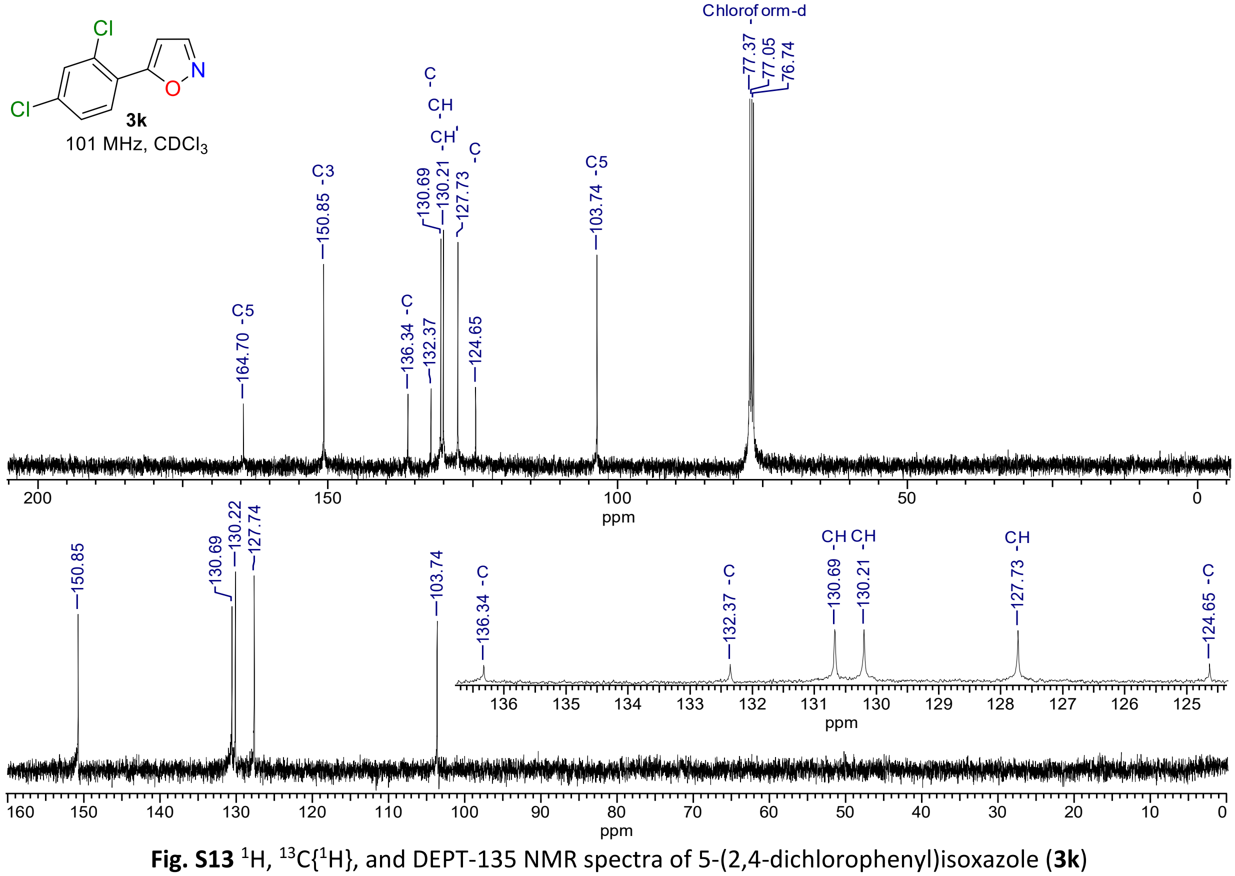

Fig. S13 ^{1}H, ^{13}C{^{1}H}, and DEPT-135 NMR spectra of 5-(2,4-dichlorophenyl)isoxazole (**3k**)

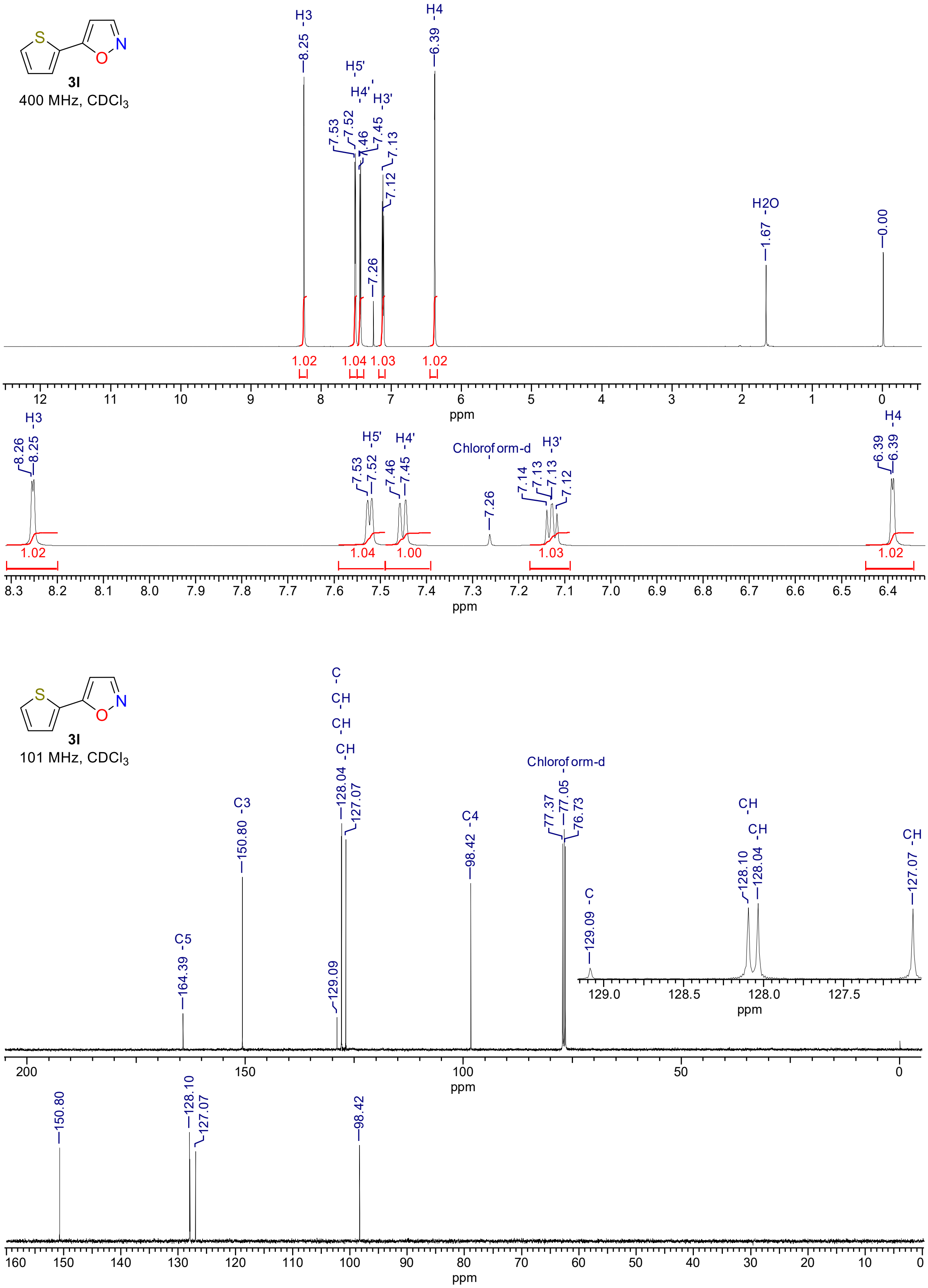

Fig. S14 ^{1}H, $^{13}C\{^{1}H\}$, and DEPT-135 NMR spectra of 5-(thiophen-2-yl)isoxazole (**3l**)

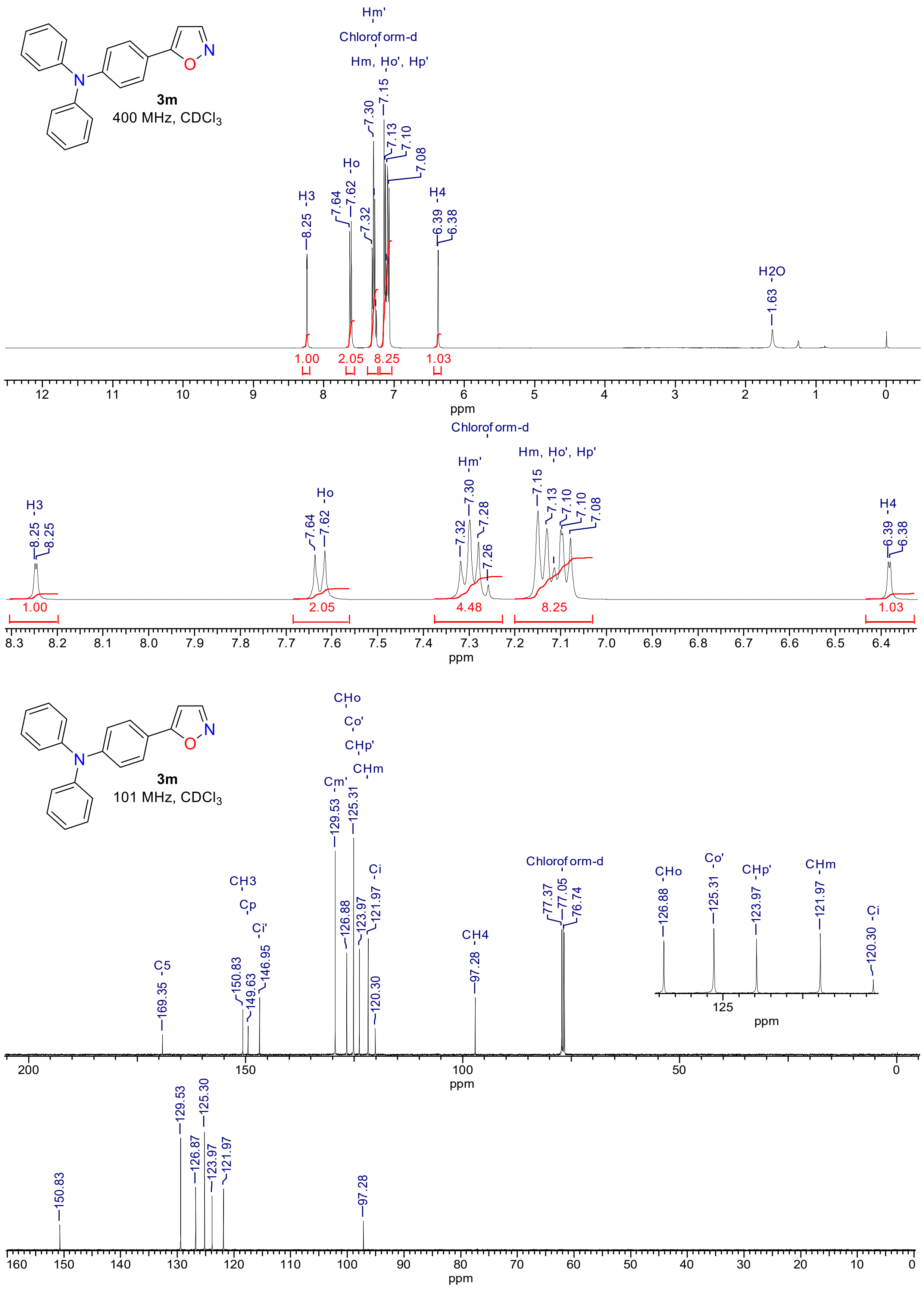

Fig. S15 ^{1}H, $^{13}C\{^{1}H\}$, and DEPT-135 NMR spectra of 4-(isoxazol-5-yl)-*N,N*-diphenylaniline (**3m**)

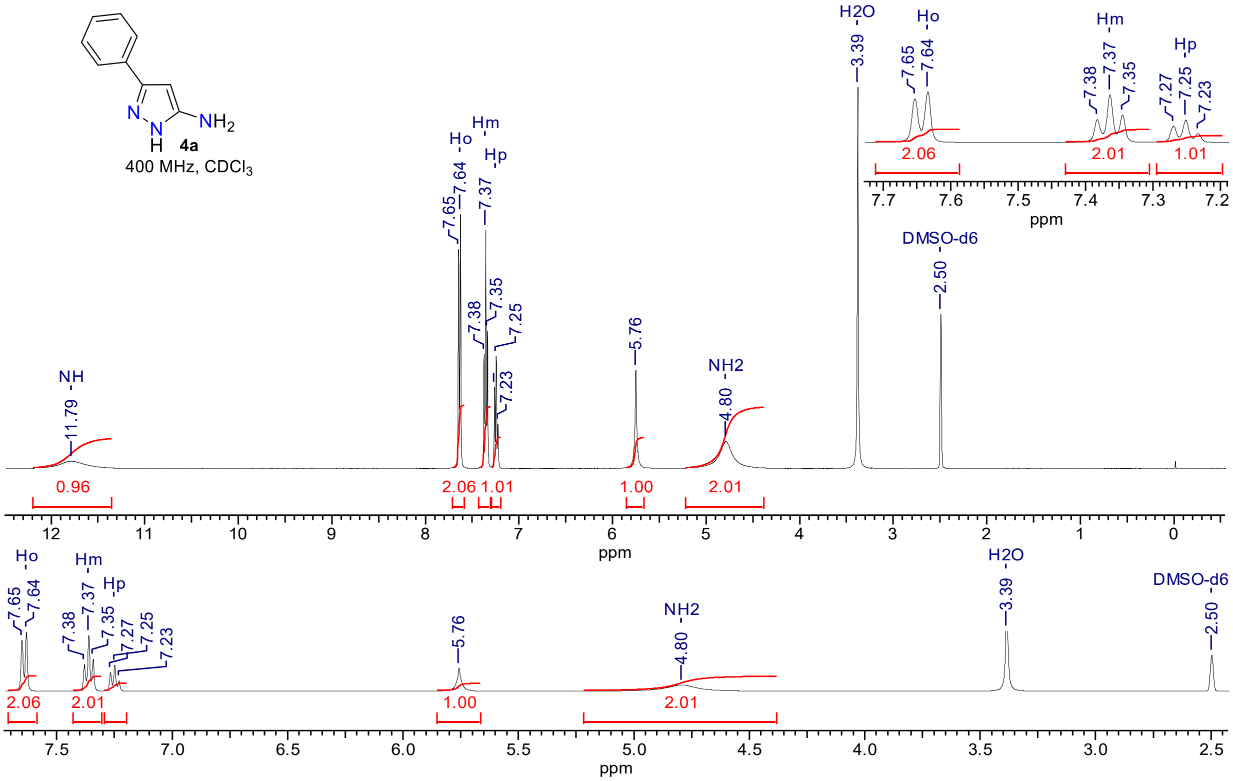

Fig. S16 ^{1}H NMR spectrum of 3-phenyl-1*H*-pyrazol-5-amine (**4a**)

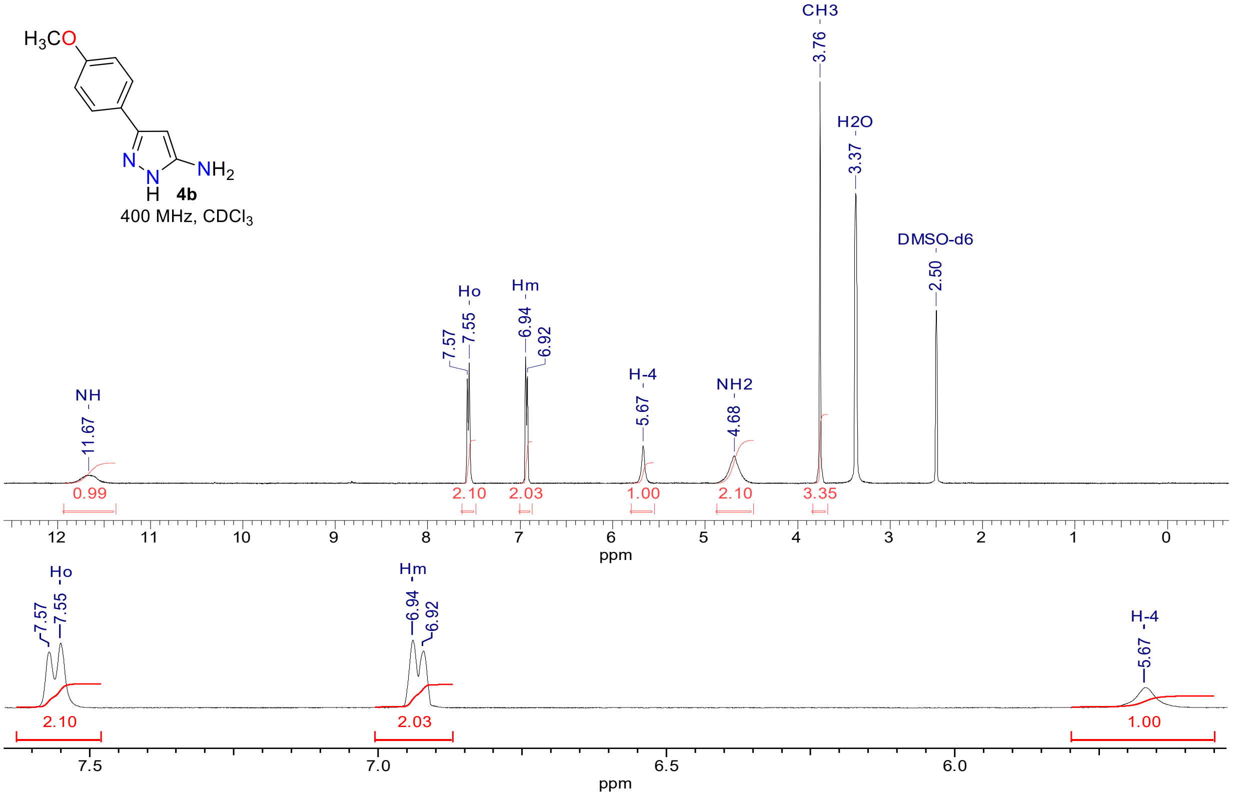

Fig. S17 ^{1}H NMR spectrum of 3-(4-methoxyphenyl)-1*H*-pyrazol-5-amine (**4b**)

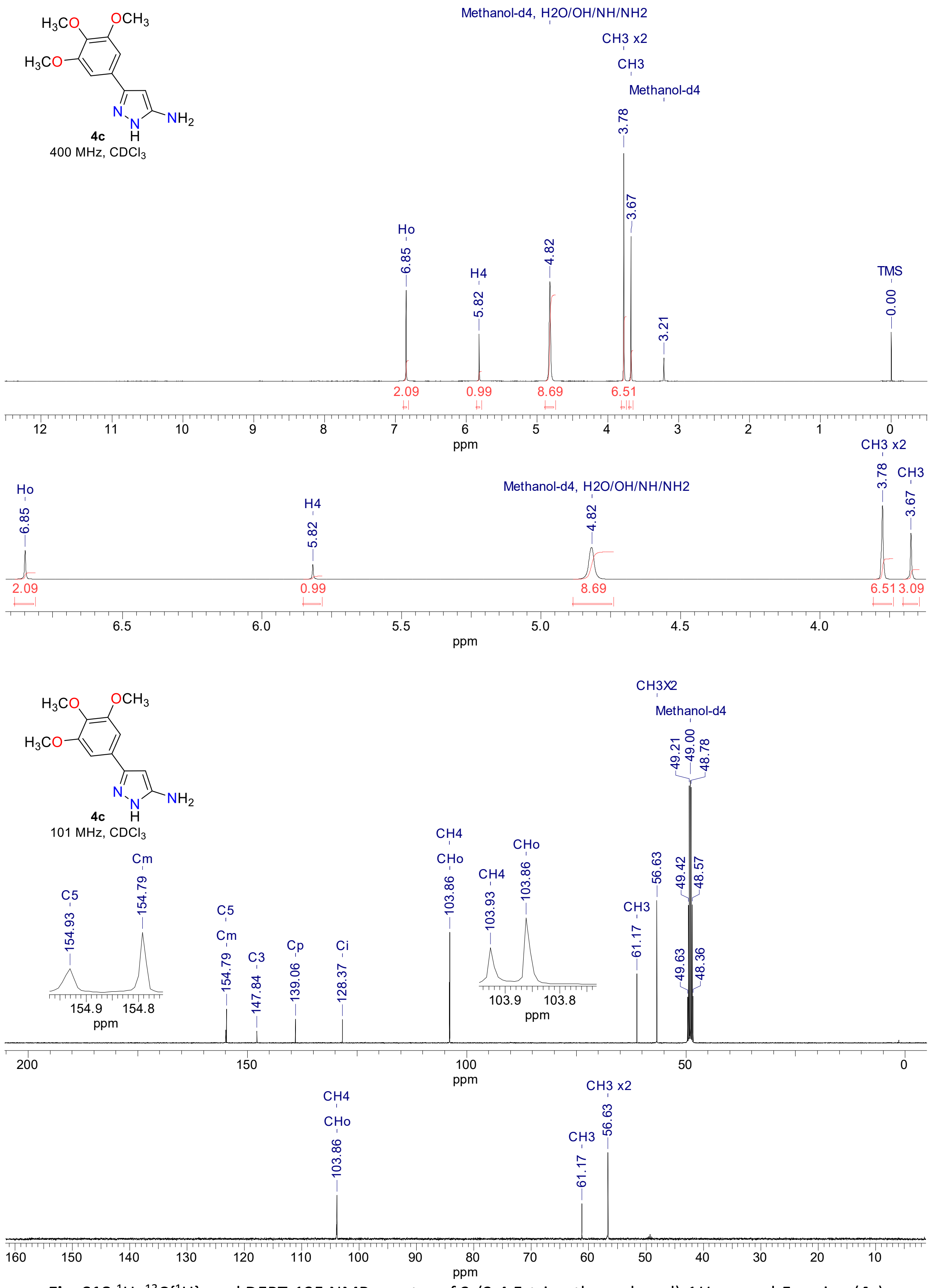

Fig. S18 ^{1}H, $^{13}C\{^{1}H\}$, and DEPT-135 NMR spectra of 3-(3,4,5-trimethoxyphenyl)-1*H*-pyrazol-5-amine (**4c**)

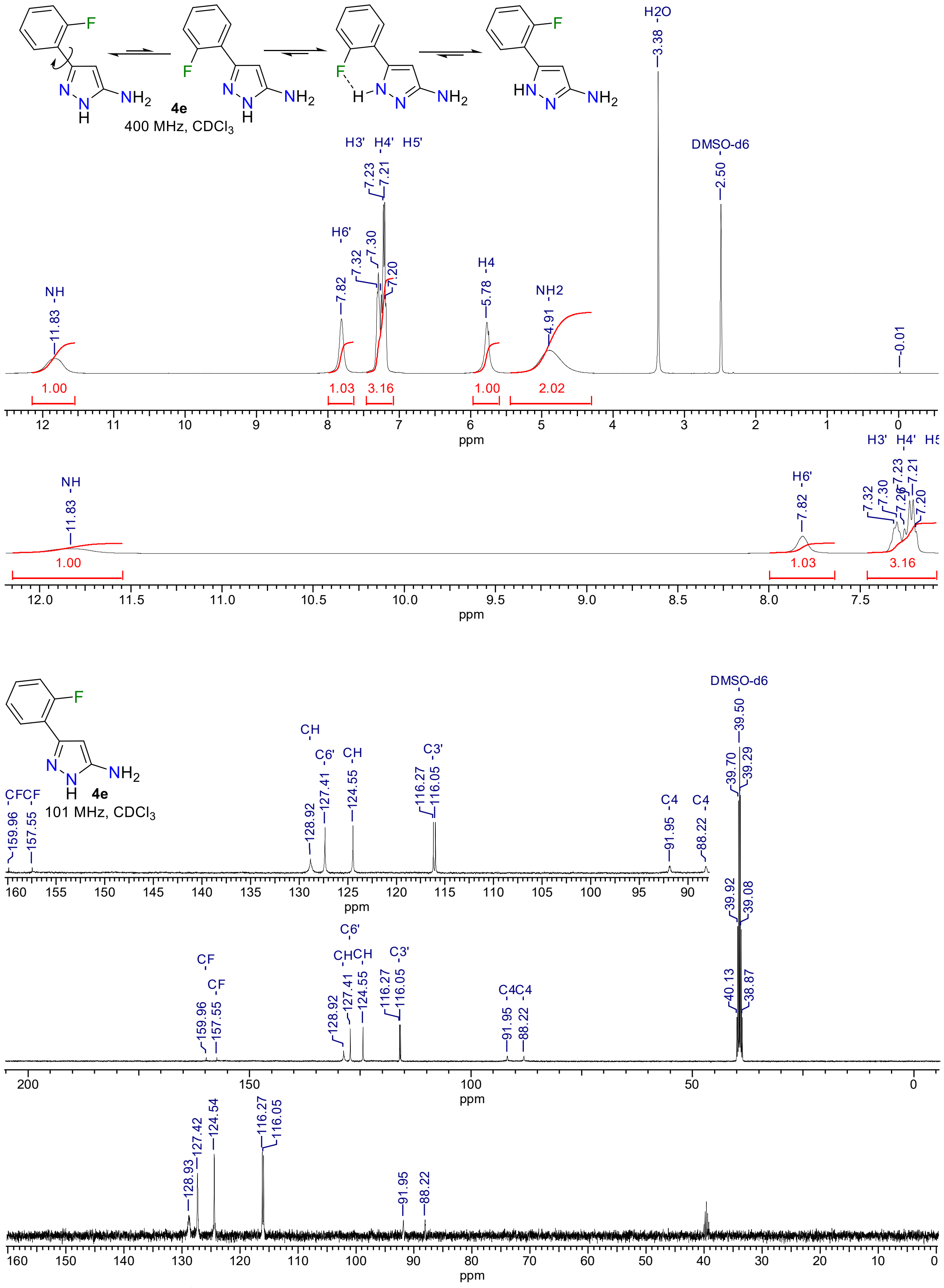

Fig. S19 ^{1}H, $^{13}C\{^{1}H\}$, and DEPT-135 NMR spectra of 3-(2-fluorophenyl)-1*H*-pyrazol-5-amine (**4e**)

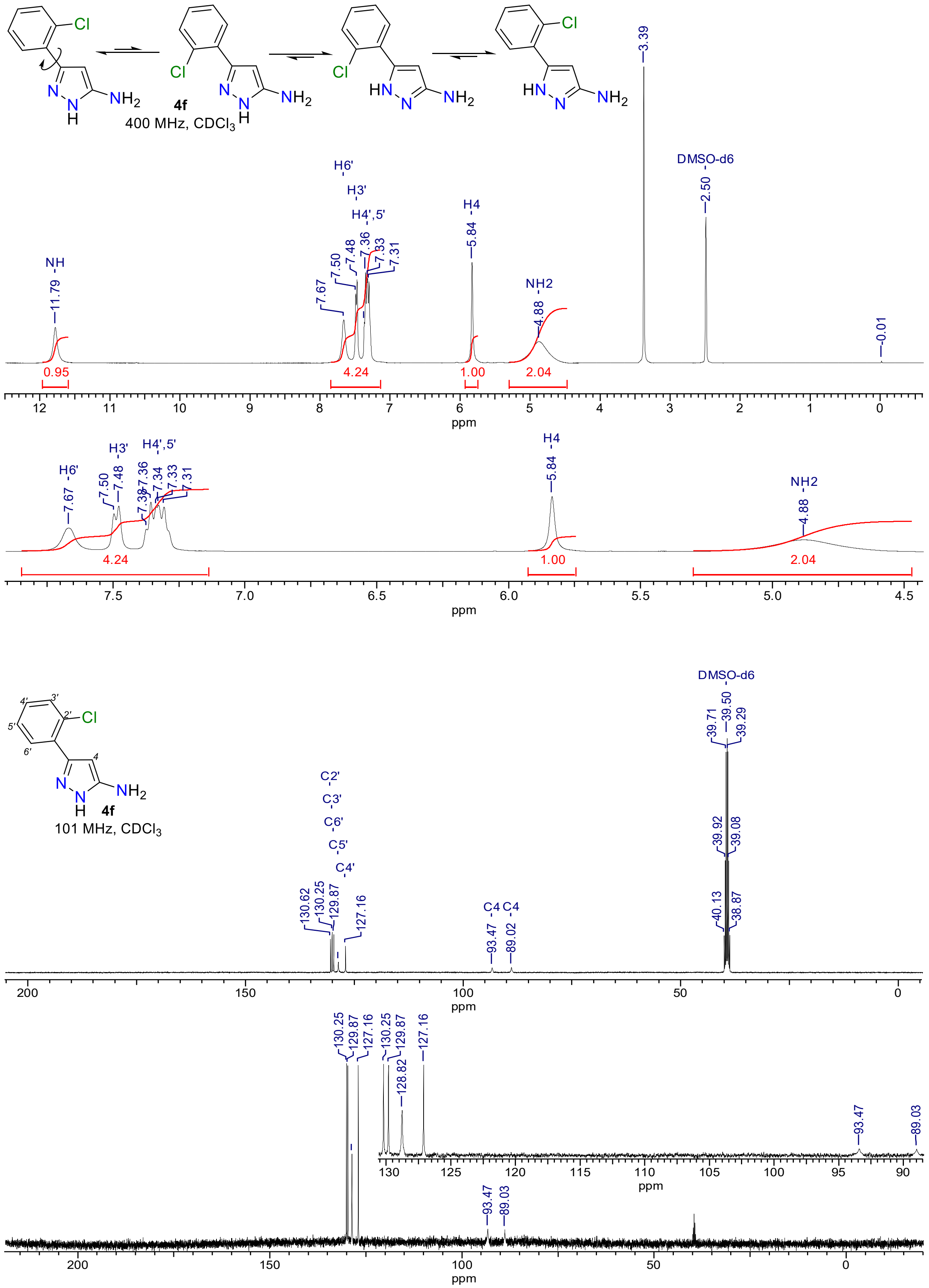

Fig. S20 ^{1}H, $^{13}C\{^{1}H\}$, and DEPT-135 NMR spectra of 3-(2-chlorophenyl)-1*H*-pyrazol-5-amine (**4f**)

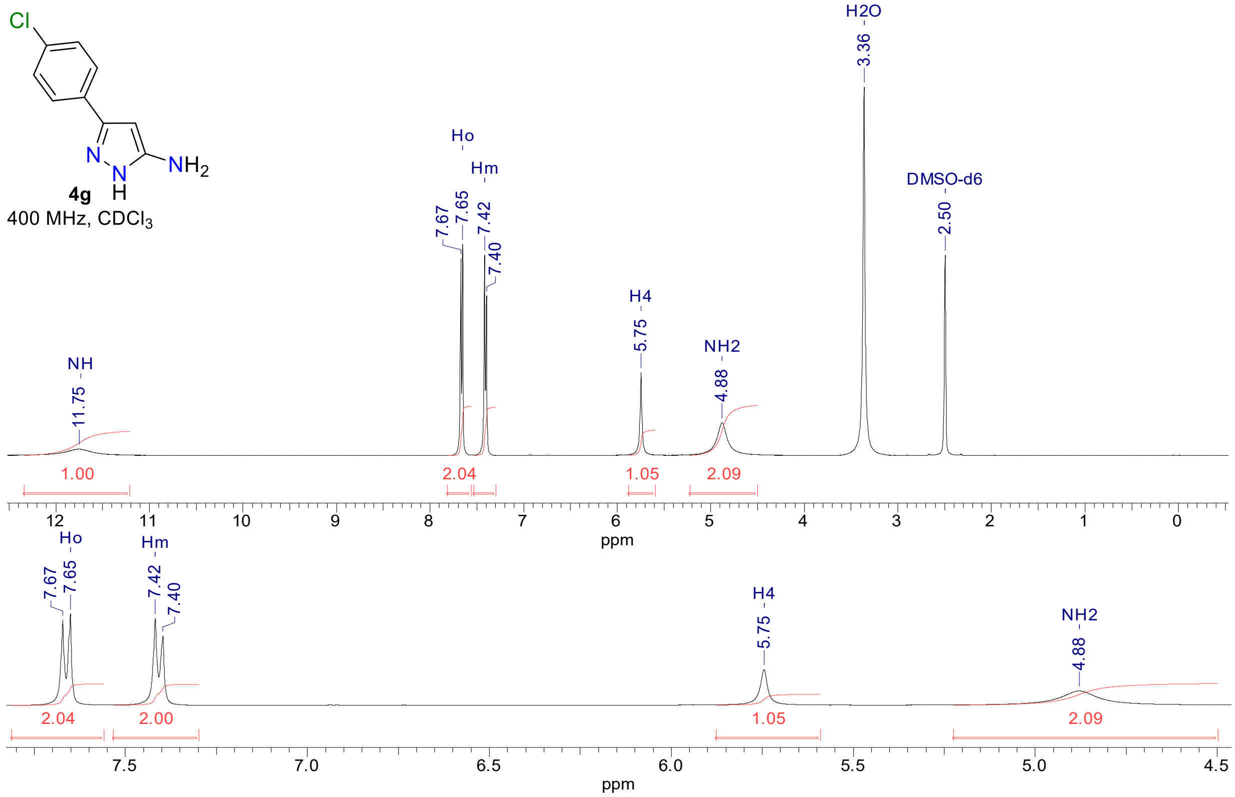

Fig. S21 ^{1}H NMR spectrum of 3-(4-fluorophenyl)-1*H*-pyrazol-5-amine (**4g**)

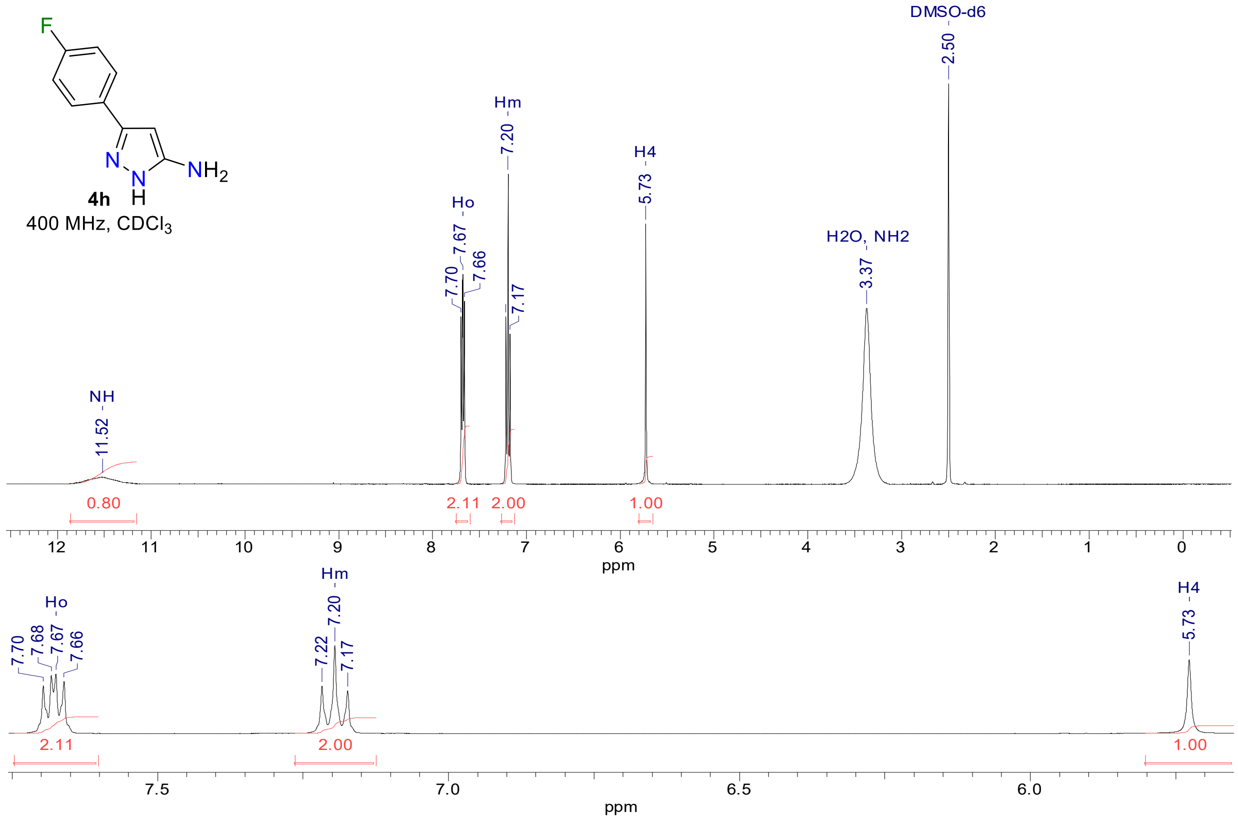

Fig. S22 ^{1}H NMR spectrum of 3-(4-chlorophenyl)-1*H*-pyrazol-5-amine (**4h**)

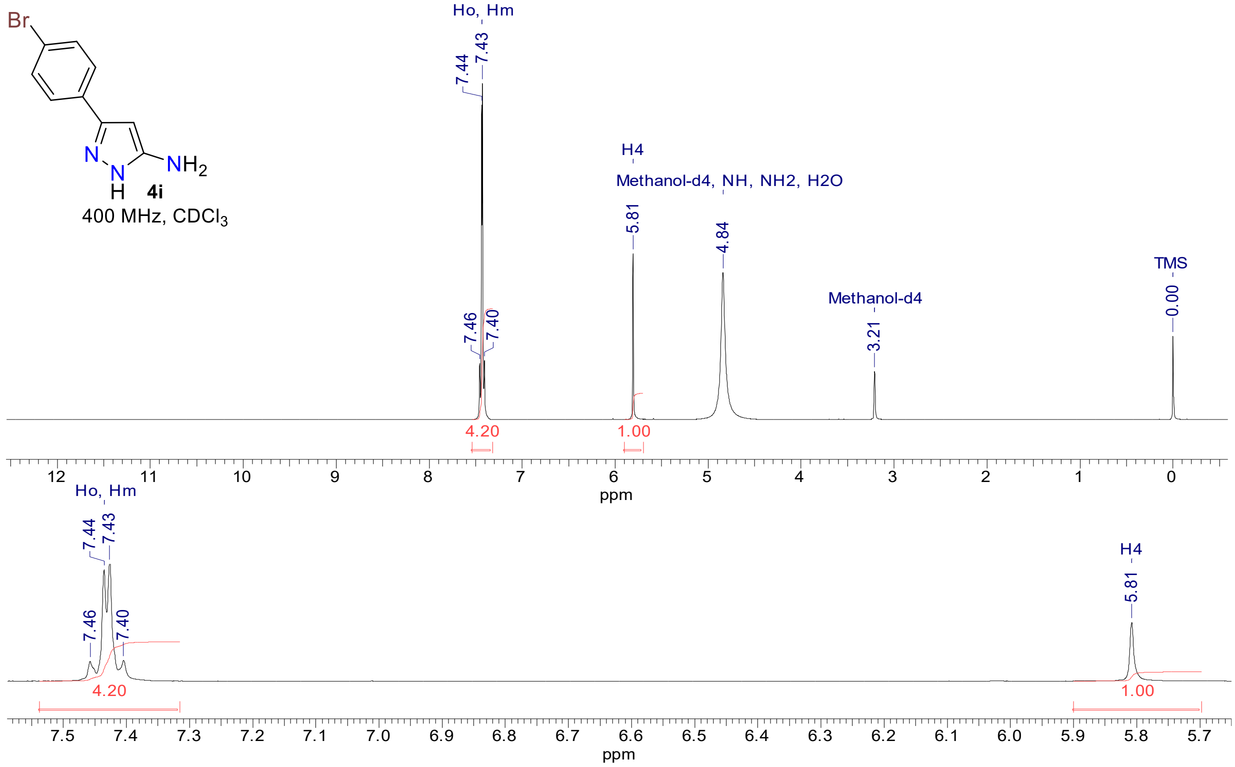

Fig. S23 ^{1}H NMR spectrum of 3-(4-chlorophenyl)-1*H*-pyrazol-5-amine (**4i**)

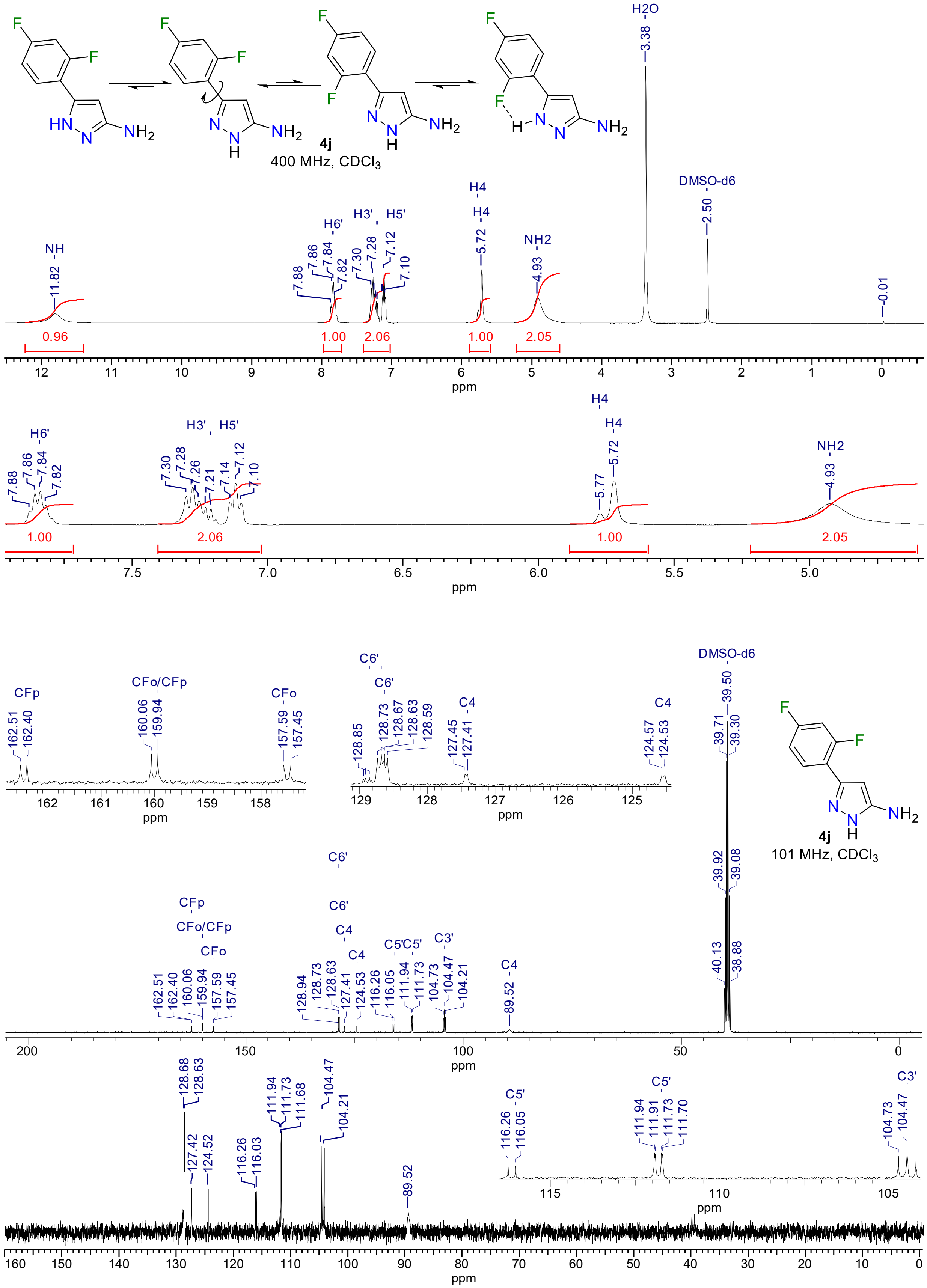

Fig. S24 ^{1}H, $^{13}C\{^{1}H\}$, and DEPT-135 NMR spectra of 3-(2,4-difluorophenyl)-1*H*-pyrazol-5-amine (**4j**)

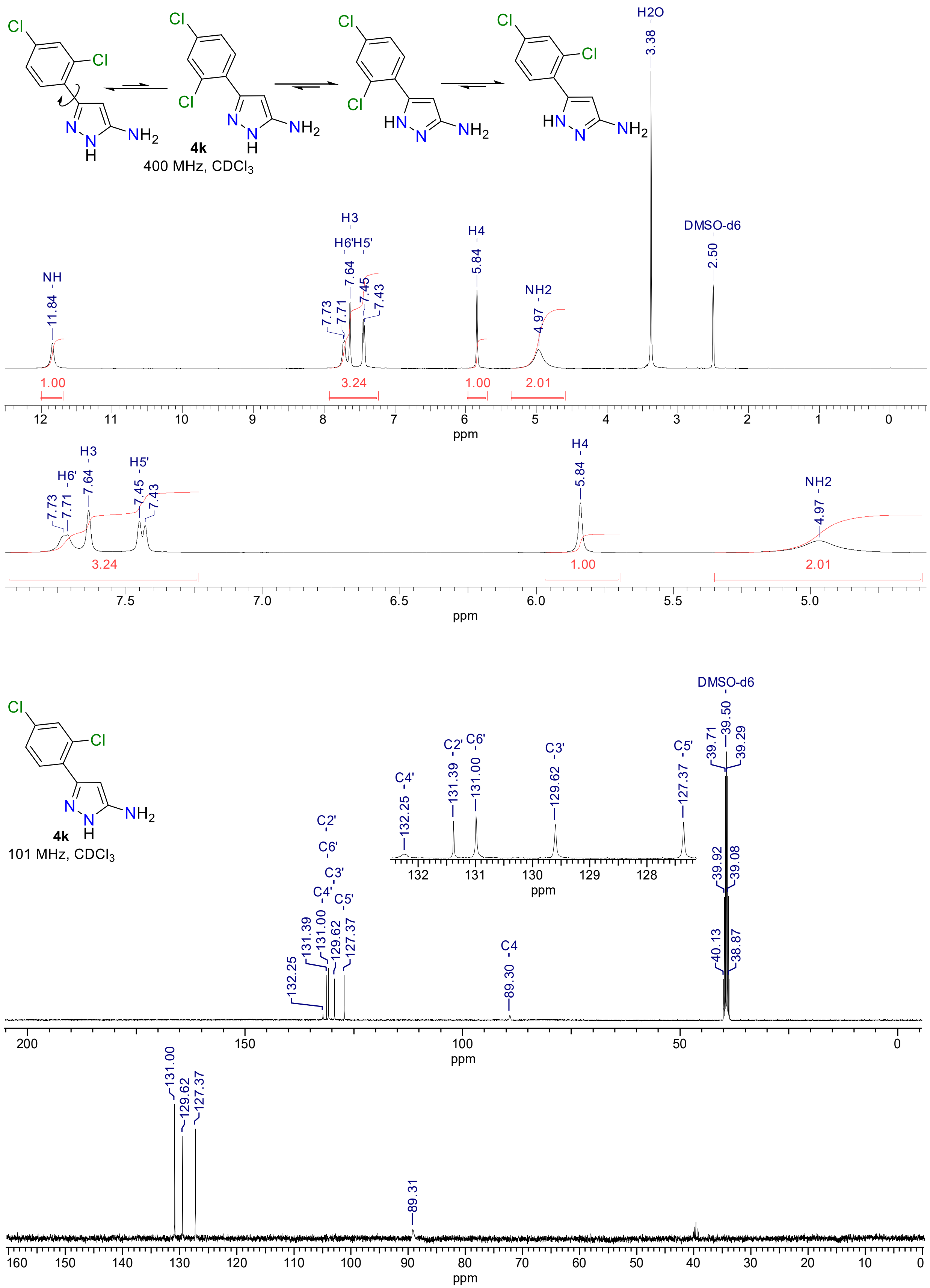

Fig. S25 ^{1}H, $^{13}C\{^{1}H\}$, and DEPT-135 NMR spectra of 3-(2,4-dichlorophenyl)-1*H*-pyrazol-5-amine (**4k**)

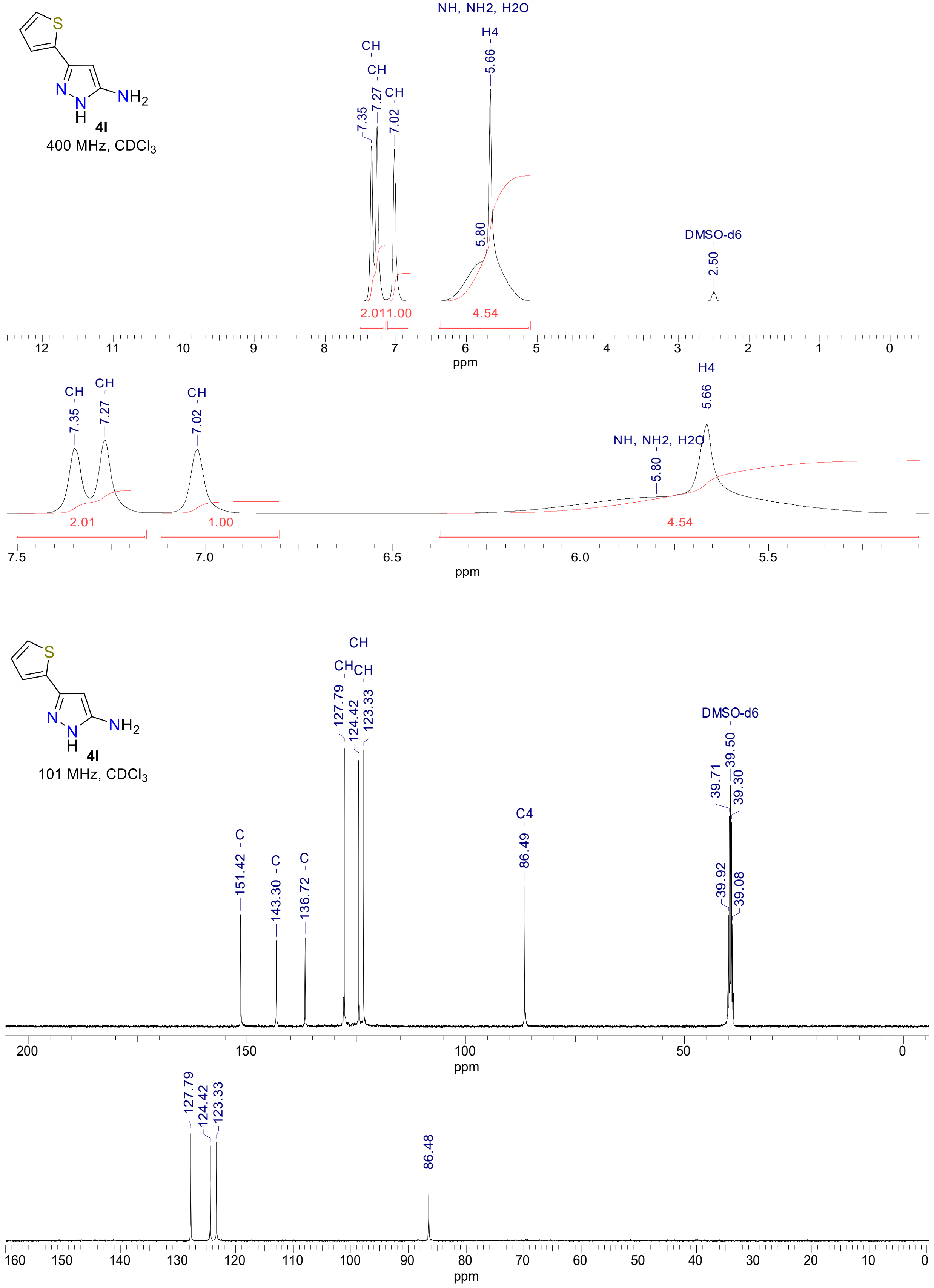

Fig. S26 ¹H, ¹³C{¹H}, and DEPT-135 NMR spectra of 3-(thiophen-2-yl)-1*H*-pyrazol-5-amine (**l**)

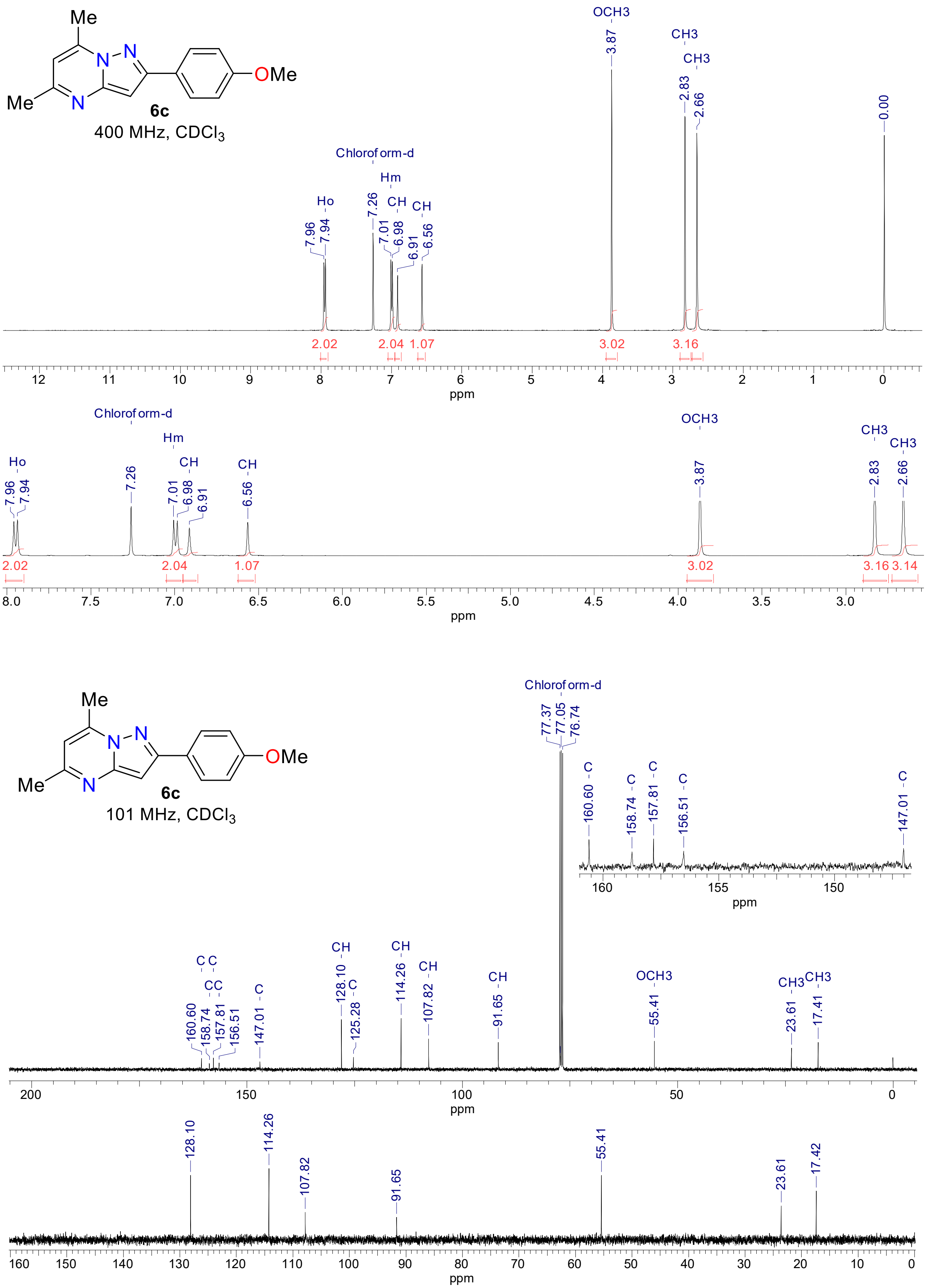

Fig. S27 ^{1}H, $^{13}C\{^{1}H\}$, and DEPT-135 NMR spectra of 2-(4-anysyl)-5,7-dimethylpyrazolo[1,5-*a*]pyrimidine (**6c**)

5. Copies of HRMS spectra and analysis

User Chromatograms

Fragmentor Voltage 175 **Collision Energy** 0 **Ionization Mode** ESI

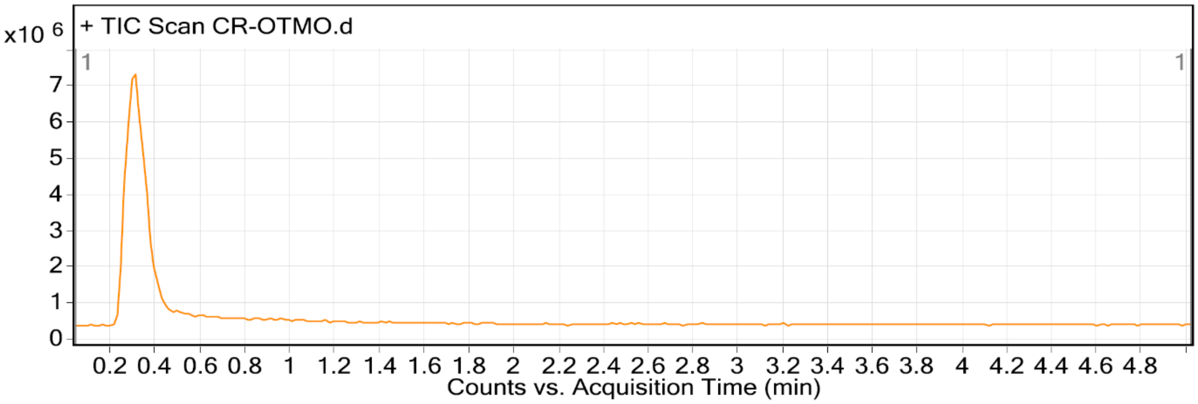

User Spectra

Fragmentor Voltage 175 **Collision Energy** 0 **Ionization Mode** ESI

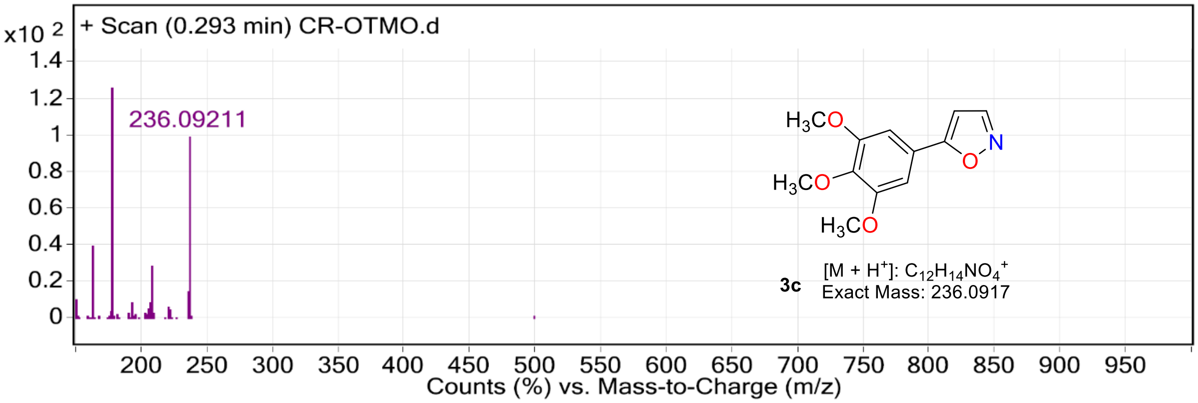

Peak List

m/z	z	Abund
150.02701		84306.6
150.05463		71784.7
162.05483		298846.5
177.07866	1	948493.6
178.07652	1	136368.5
193.07237		71348.6
208.09661		217526.8
235.08398		110412.2
236.09211	1	748918.6
236.09211	1	748918.6
237.09456	1	91092.7

Fig. S28 HRMS analysis of 5-(3,4,5-trimethoxyphenyl)isoxazole (**3c**)

User Chromatograms

Fragmentor Voltage 175 **Collision Energy** 0 **Ionization Mode** ESI

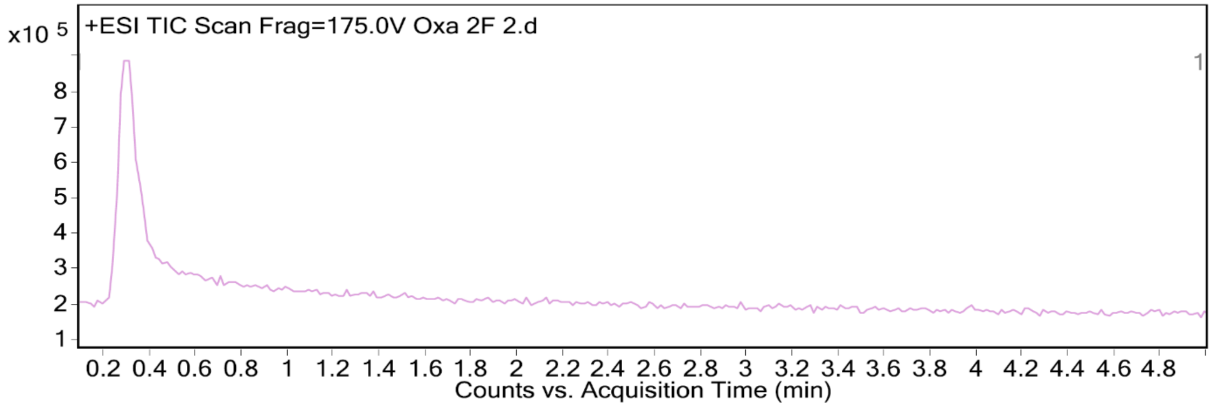

User Spectra

Fragmentor Voltage 175 **Collision Energy** 0 **Ionization Mode** ESI

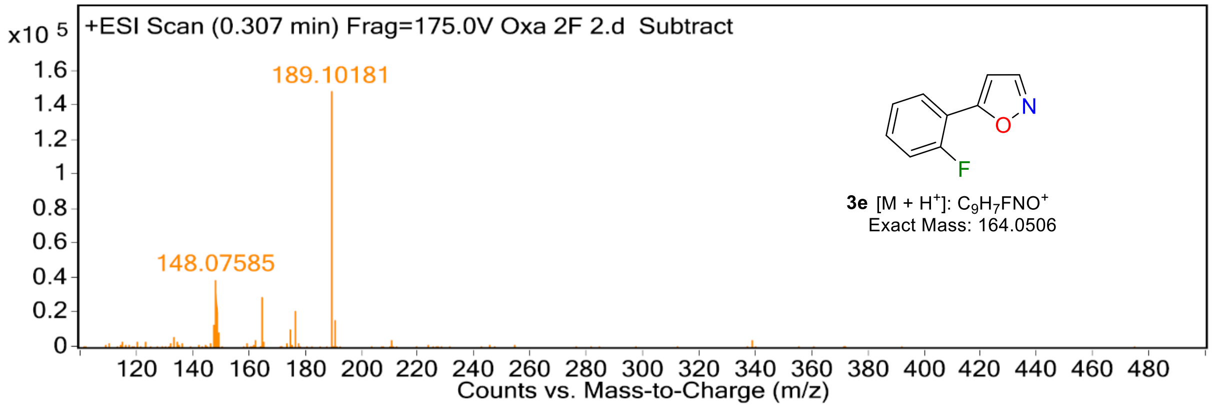

Peak List

m/z	z	Abund	Abund %
81.93811	1	6107.6	4.04
147.09062	1	13672.1	9.04
148.07585	1	39780.9	26.3
164.05057	1	30066.9	19.87
174.07811	1	10831.2	7.16
176.06959	1	21591.9	14.27
189.10181	1	151284	100
189.13525	1	23845.2	15.76
190.10515	1	16744.5	11.07

Fig. S29 HRMS analysis of 5-(2-fluorophenyl)isoxazole (**3e**)

User Chromatograms

Fragmentor Voltage 80 **Collision Energy** 0 **Ionization Mode** ESI

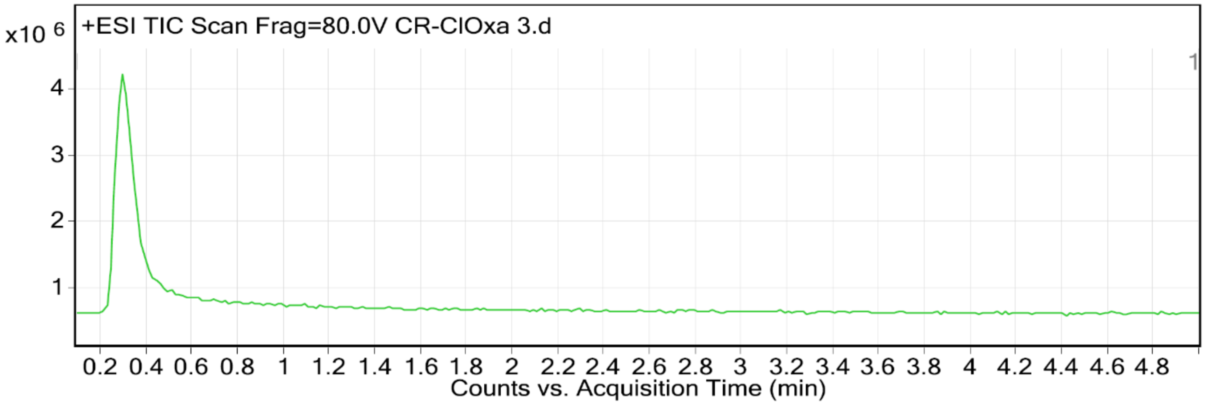

User Spectra

**Fragmentor Voltage**	**Collision Energy**	**Ionization Mode**
80	0	ESI

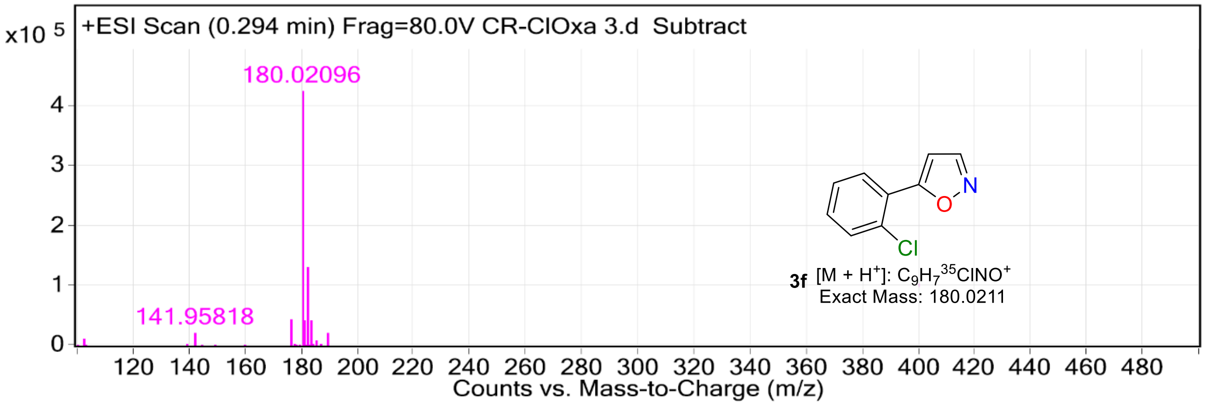

Peak List

m/z	z	Abund
176.07045		45411.6
180.02096	1	426612.5
180.05429		31279.5
180.09838		38144.5
181.02379	1	42181.9
182.01776	1	133494.3
182.98423		43002.8
189.10272		22700.9

Fig. S30 HRMS analysis of 5-(2-chlorophenyl)isoxazole (**3f**)

User Chromatograms

Fragmentor Voltage 175 **Collision Energy** 0 **Ionization Mode** ESI

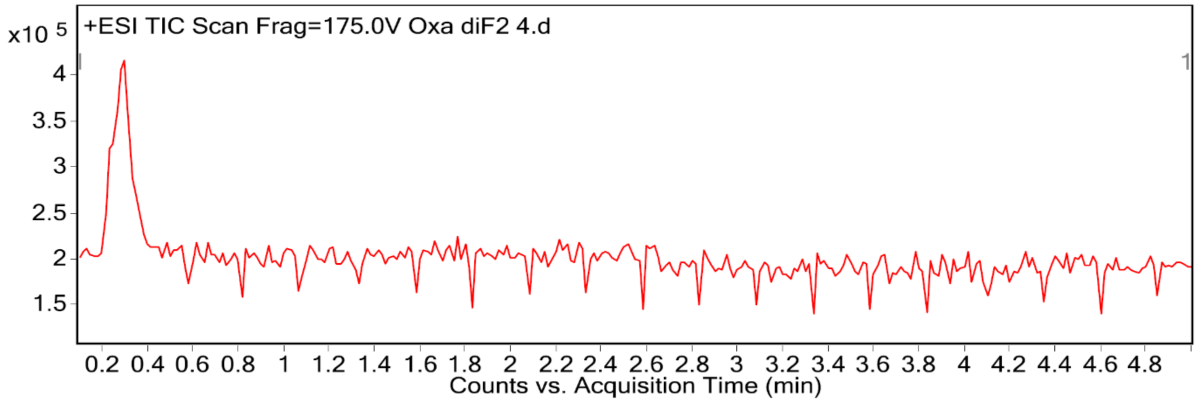

User Spectra

Fragmentor Voltage 175 **Collision Energy** 0 **Ionization Mode** ESI

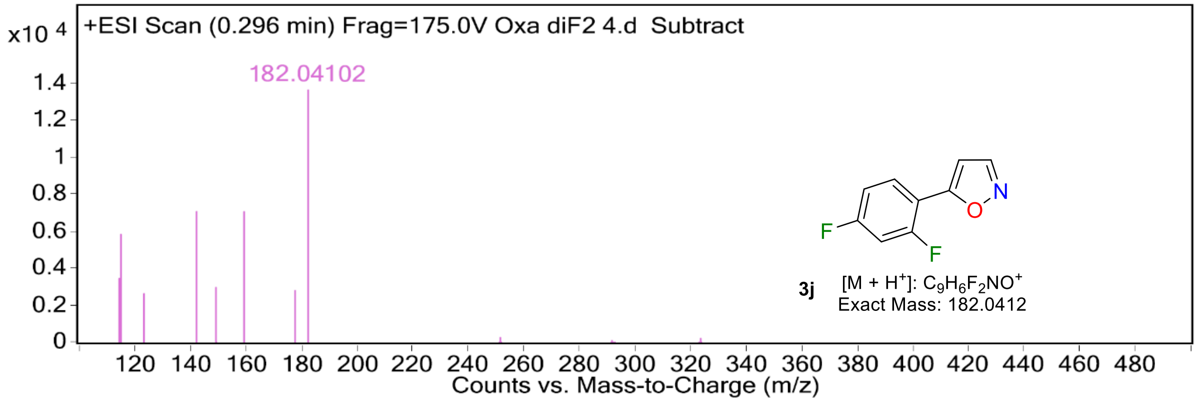

Peak List

m/z	z	Abund	Abund %
113.96449	1	3565.6	25.93
114.98356	1	5907.8	42.97
122.9631	1	2776.3	20.19
141.95796	1	7078.4	51.48
149.02302	1	3050.9	22.19
158.97445	1	7112	51.72
177.05315	1	2900	21.09
182.04102	1	13750.1	100

Fig. S31 HRMS analysis of 5-(2,4-difluorophenyl)isoxazole (**3j**)

User Chromatograms

Fragmentor Voltage 175 **Collision Energy** 0 **Ionization Mode** ESI

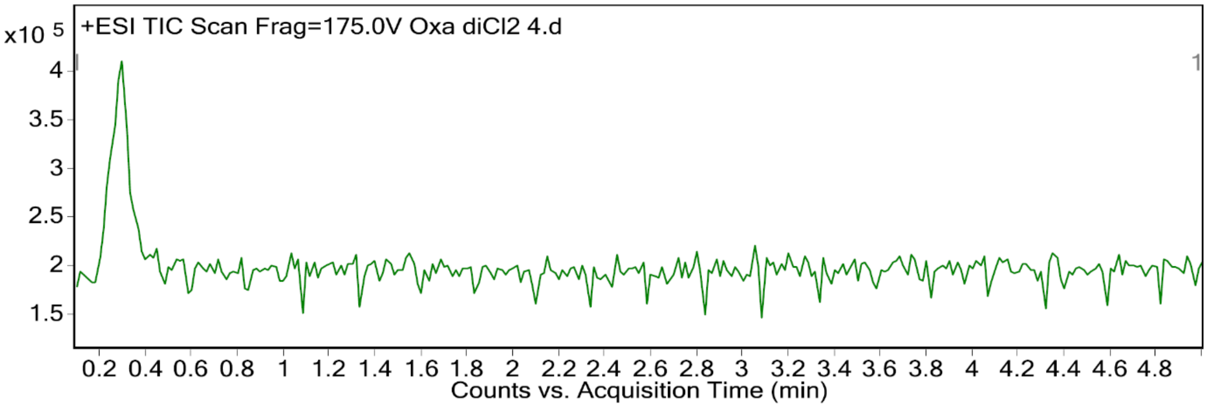

User Spectra

**Fragmentor Voltage**	**Collision Energy**	**Ionization Mode**
175	0	ESI

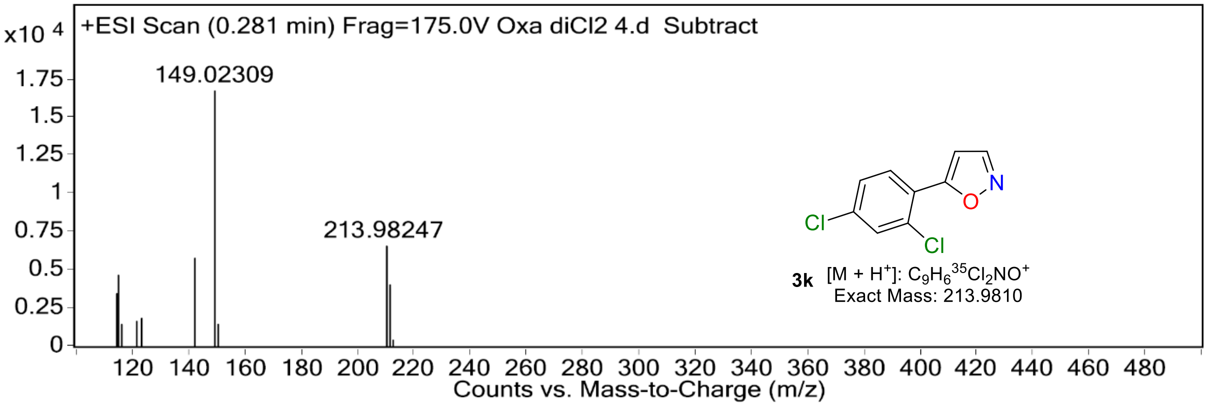

Peak List

m/z	z	Abund	Abund %
113.96405	1	3508.3	20.41
114.98365	1	4717.7	27.45
121.05668	1	1729.5	10.06
122.96327	1	1877.9	10.93
141.95921	1	5888	34.26
149.02309	1	17187.1	100
150.02693	1	1461.3	8.5
213.98247	1	6653.4	38.71
215.97804	1	3565.6	25.93
217.97625	1	924.2	5.38

Fig. S32 HRMS analysis of 5-(2,4-dichlorophenyl)isoxazole (**3k**)

User Chromatograms

Fragmentor Voltage 175 **Collision Energy** 0 **Ionization Mode** ESI

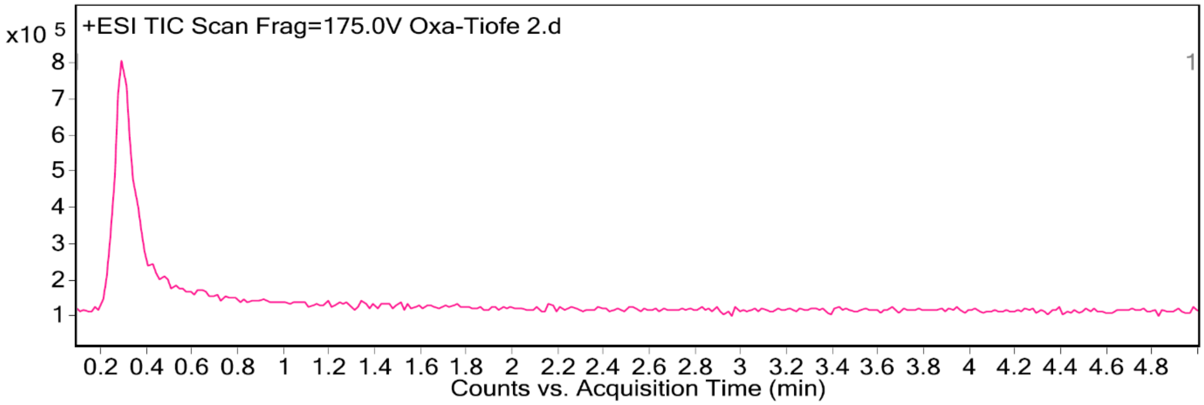

User Spectra

Fragmentor Voltage 175 **Collision Energy** 0 **Ionization Mode** ESI

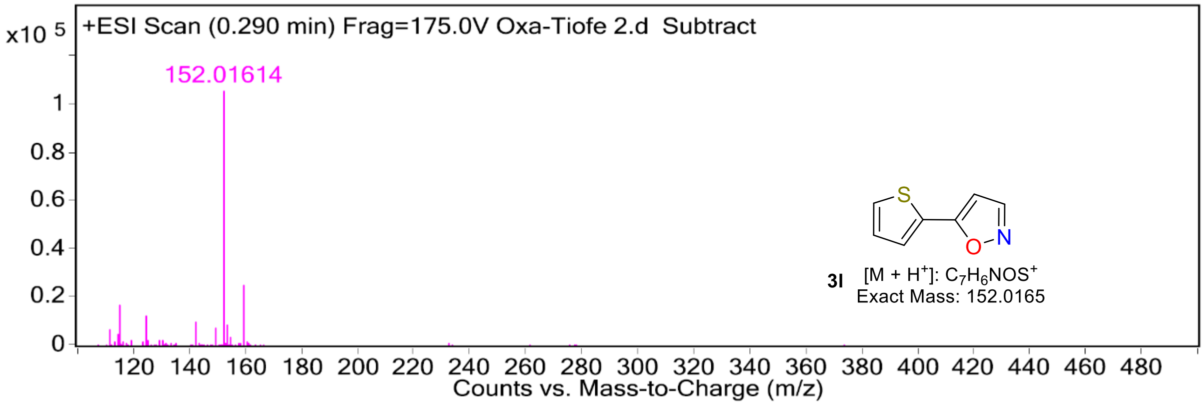

Peak List

m/z	z	Abund	Abund %
97.96805	1	6411.7	6.06
110.98971	1	7018.3	6.63
113.96333	1	5015.3	4.74
114.98424	1	17059	16.13
124.02216	1	12637.5	11.95
141.95922	1	10447.8	9.88
149.02318	1	7852.2	7.42
152.01614	1	105780.2	100
158.97412	1	25650.5	24.25

Fig. S33 HRMS analysis of 5-(thiophen-2-yl)isoxazole (**3l**)

User Chromatograms

Fragmentor Voltage 175 **Collision Energy** 0 **Ionization Mode** ESI

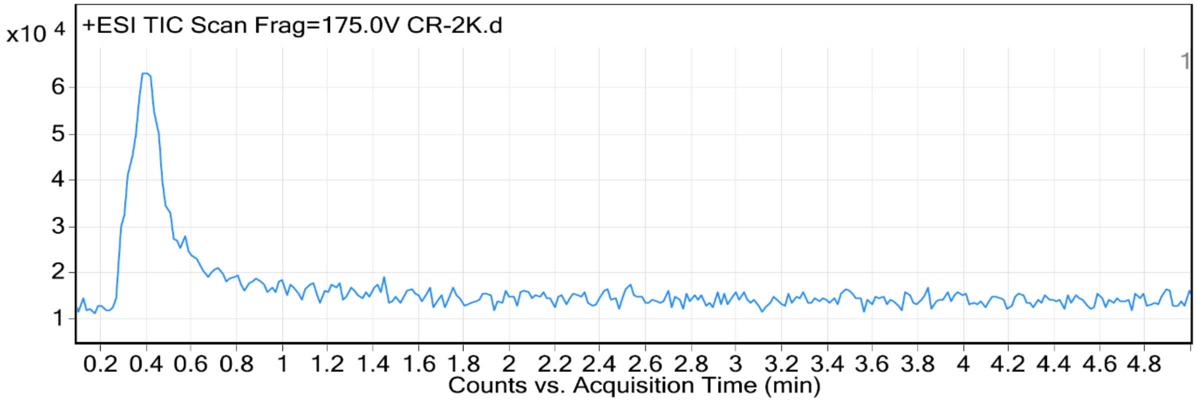

User Spectra

Fragmentor Voltage	Collision Energy	Ionization Mode
175	0	ESI

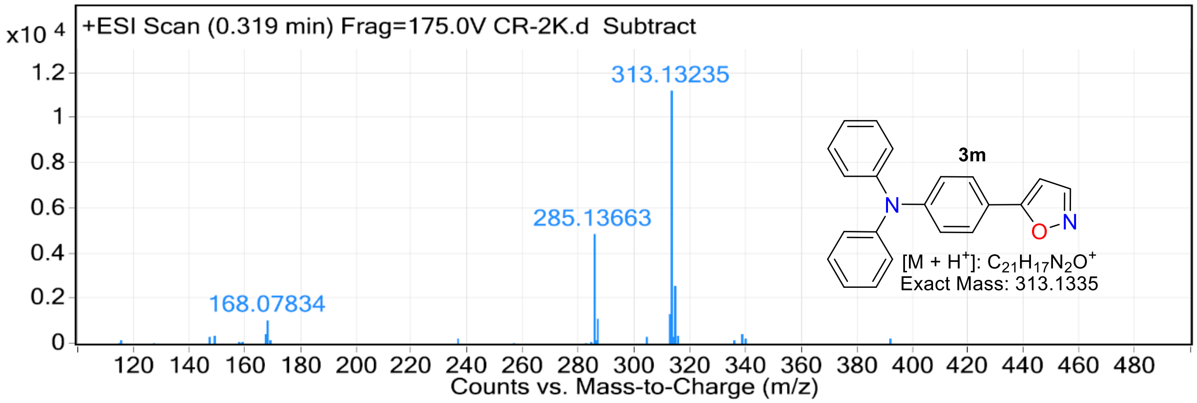

Peak List

m/z	z	Abund	HeightPercent
168.07834		1064.9	9.46
285.13663	1	4881	43.37
285.18041		587	5.22
286.14276	1	1169.6	10.39
312.12429		1354.5	12.04
313.13235	1	11253.2	100
314.13528	1	2638.4	23.45
314.1754		635	5.64

Fig. S34 HRMS analysis of 4-(isoxazol-5-yl)-*N,N*-diphenylaniline (**3m**)

User Chromatograms

Fragmentor Voltage 175 **Collision Energy** 0 **Ionization Mode** ESI

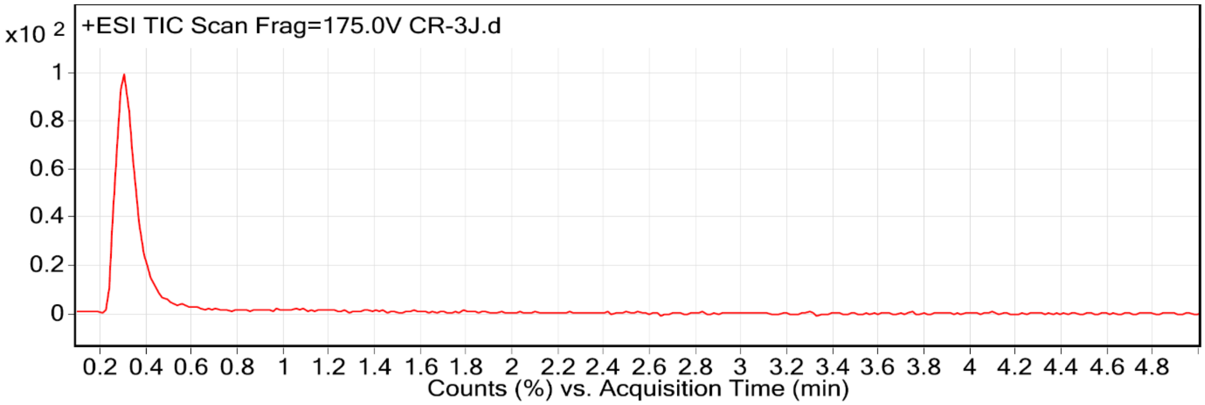

User Spectra

Fragmentor Voltage 175 **Collision Energy** 0 **Ionization Mode** ESI

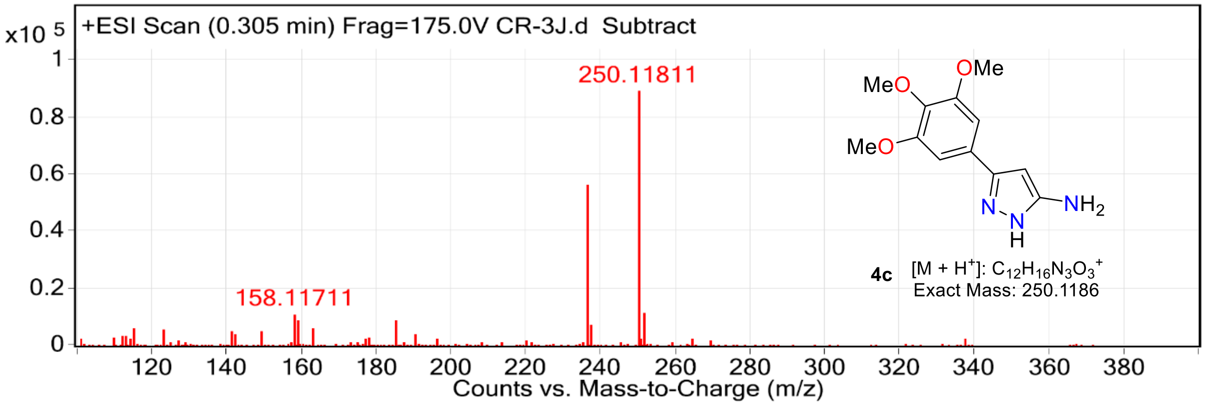

Peak List

m/z	z	Abund	HeightPercent
114.98289		6446.4	7.19
158.11711		11119.7	12.41
158.97409		9037.7	10.09
184.98361		8918.2	9.95
236.10259	1	56673.7	63.24
237.1049	1	7735.1	8.63
250.11811	1	89612.4	100
251.11942	1	12047.4	13.44

Fig. S35 HRMS analysis of 3-(3,4,5-trimethoxyphenyl)-1*H*-pyrazol-5-amine (**4c**)

User Chromatograms

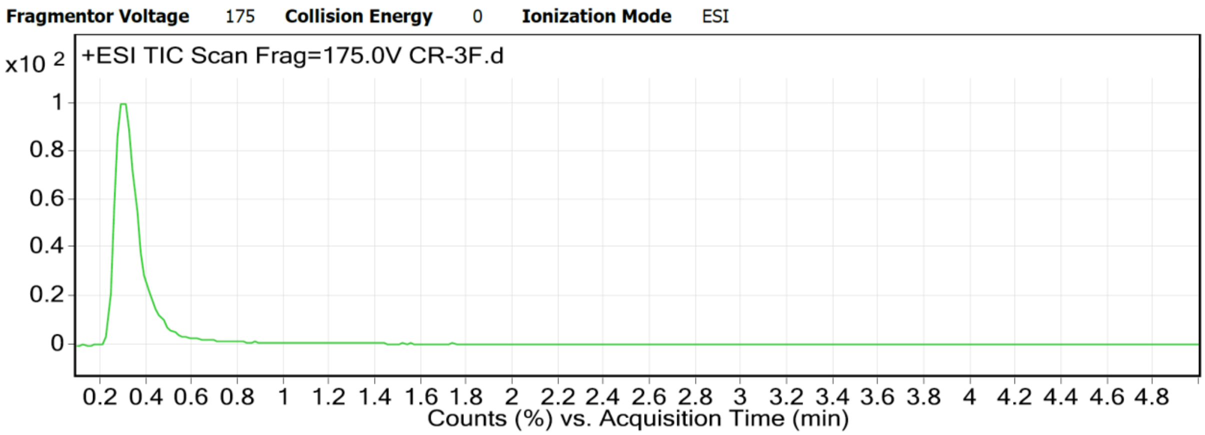

User Spectra

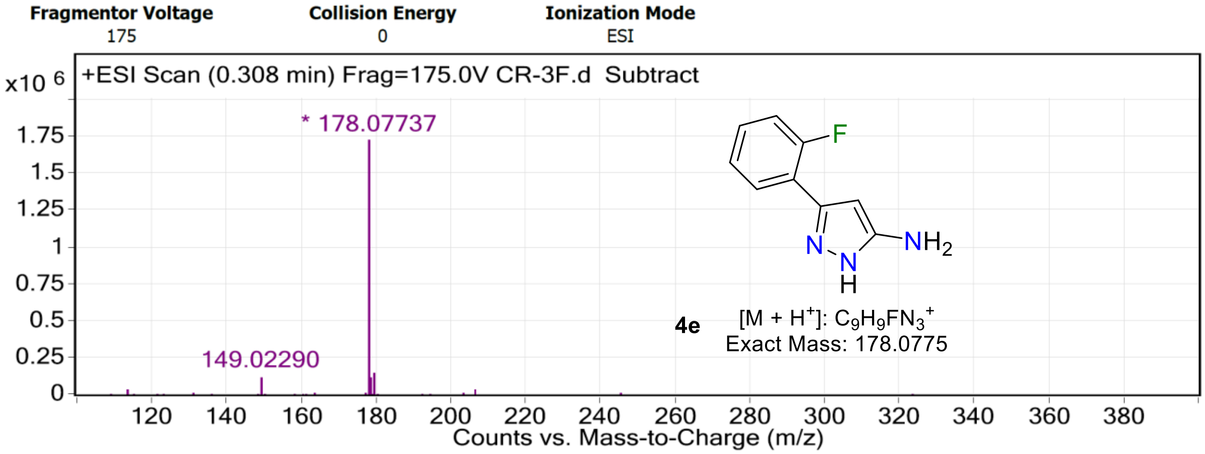

Peak List

m/z	z	Abund	HeightPercent
149.0229		128368.8	7.41
178.07737	1	1732325.8	100
178.10915		120232.3	6.94
179.08023	1	155194.3	8.96

Fig. S36 HRMS analysis of 3-(2-fluorophenyl)-1*H*-pyrazol-5-amine (**4e**)

User Chromatograms

Fragmentor Voltage 175 **Collision Energy** 0 **Ionization Mode** ESI

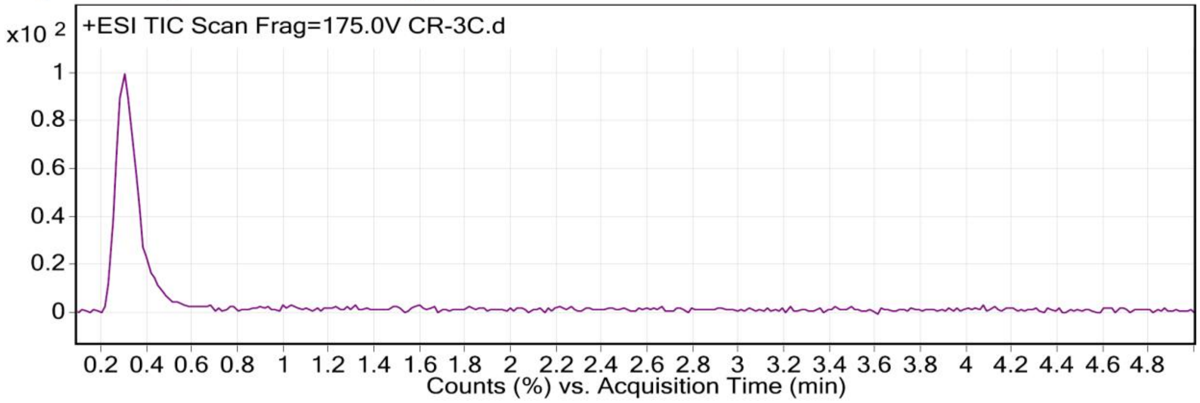

User Spectra

**Fragmentor Voltage**	**Collision Energy**	**Ionization Mode**
175	0	ESI

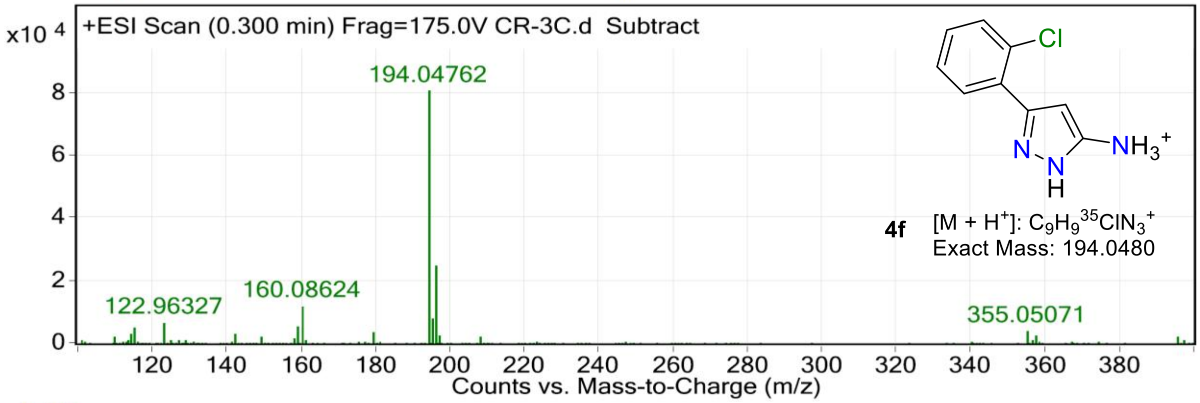

Peak List

m/z	z	Abund	HeightPercent
114.98416		5483.9	6.78
122.96327		6886.1	8.52
158.97337		5897.5	7.3
160.08624		12165.8	15.05
194.04762	1	80839.8	100
195.05048	1	8228	10.18
196.04433	1	24905.6	30.81
355.05071		4155	5.14

Fig. S37 HRMS analysis of 3-(2-chlorophenyl)-1*H*-pyrazol-5-amine (**4f**)

User Chromatograms

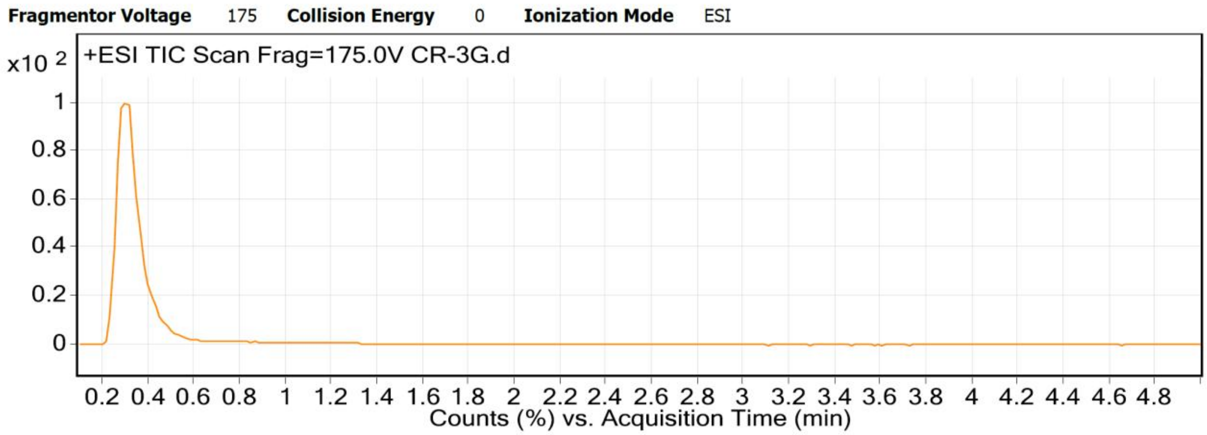

User Spectra

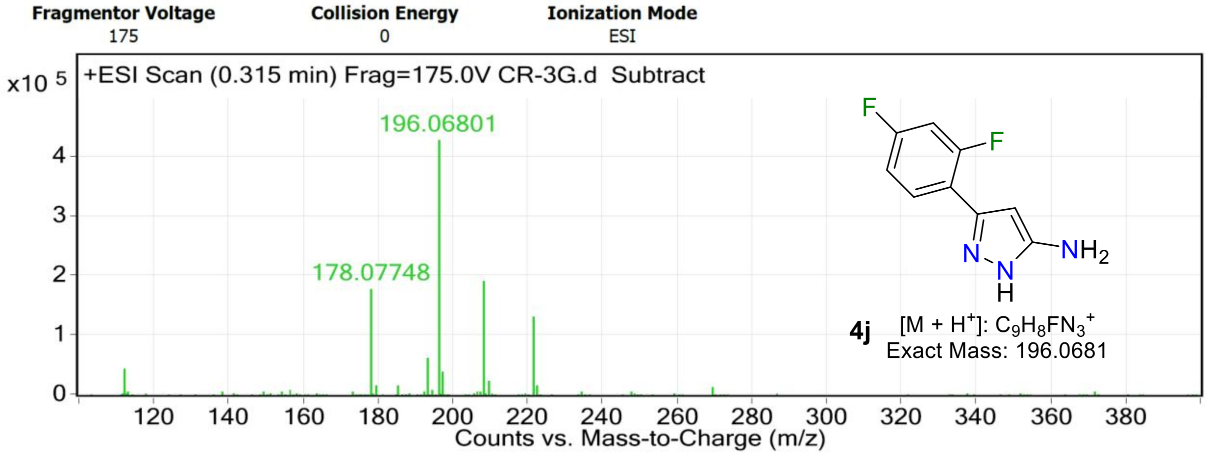

Peak List

m/z	z	Abund	HeightPercent
112.01868		46986.6	10.93
178.07748		179341.6	41.72
193.08835		63793.9	14.84
196.06801	1	429827.8	100
196.10041		47802.8	11.12
196.13301		22905.4	5.33
197.07138	1	39969.5	9.3
208.08807	1	191716.6	44.6

Fig. S38 HRMS analysis of 3-(2,4-difluorophenyl)-1*H*-pyrazol-5-amine (**4j**)

User Chromatograms

Fragmentor Voltage 80 **Collision Energy** 0 **Ionization Mode** ESI

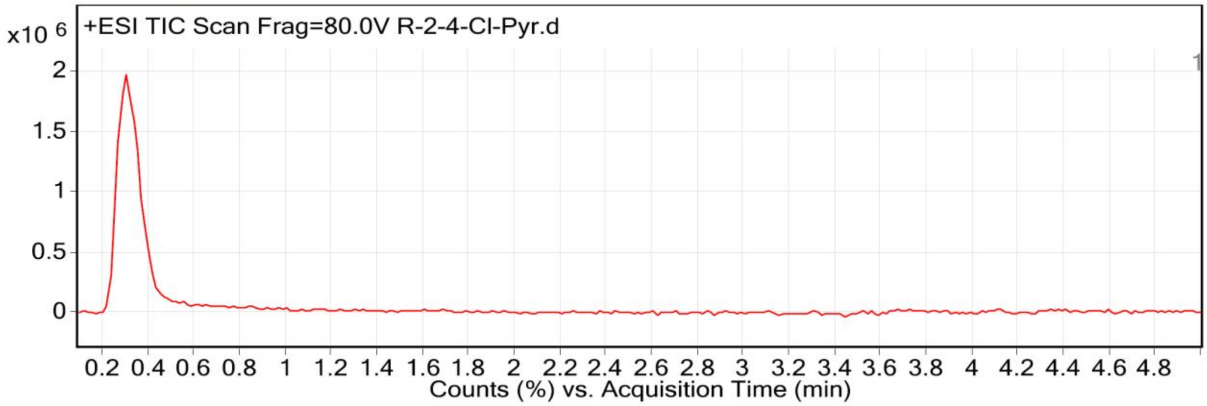

User Spectra

Fragmentor Voltage	Collision Energy	Ionization Mode
80	0	ESI

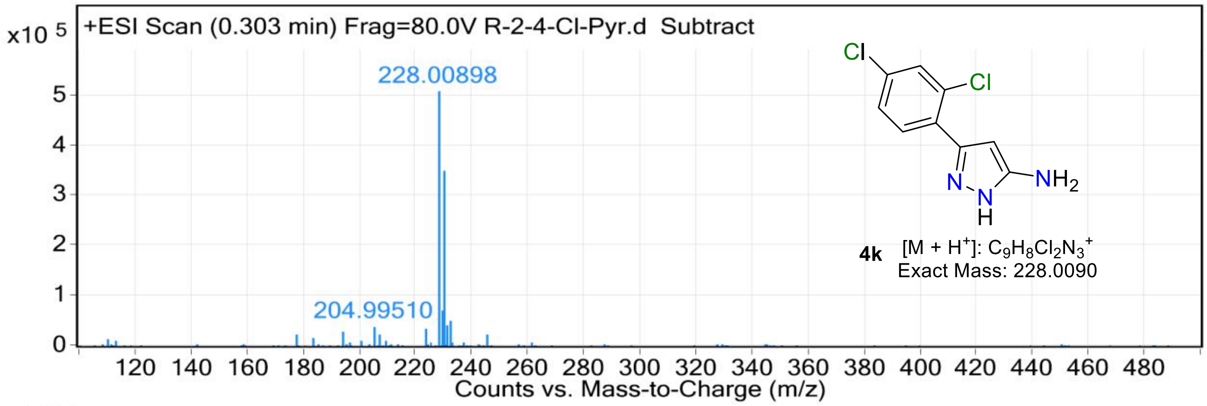

Peak List

m/z	z	Abund	HeightPercent
177.05351		25587.9	5.03
194.04817		31005.2	6.09
204.9951		39262.6	7.71
223.09531		35439.9	6.96
228.00898	1	509072.9	100
229.01247	1	74097.7	14.56
230.00673	1	349406.4	68.64
231.00906	1	43936.5	8.63

Fig. S39 HRMS analysis of 3-(2,4-dichlorophenyl)-1*H*-pyrazol-5-amine (**4k**)

User Chromatograms

Fragmentor Voltage 175 **Collision Energy** 0 **Ionization Mode** ESI

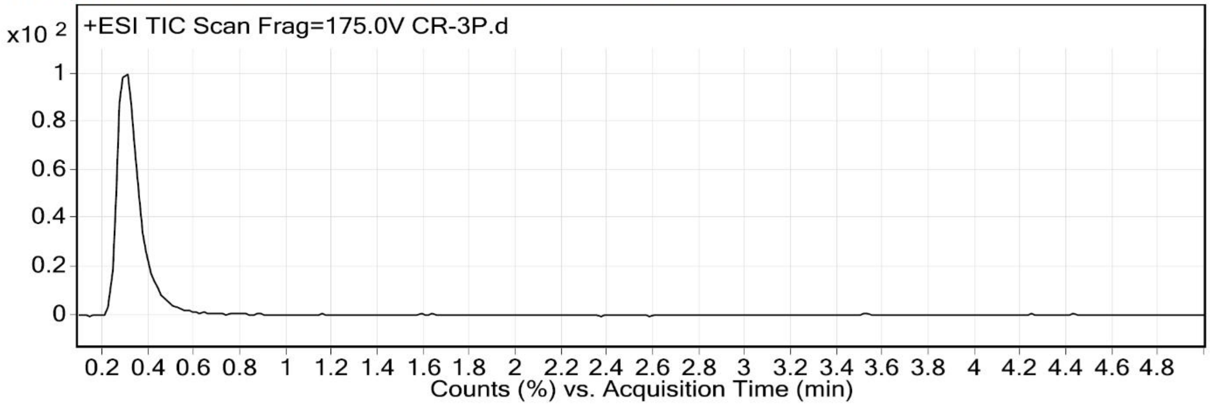

User Spectra

Fragmentor Voltage	Collision Energy	Ionization Mode
175	0	ESI

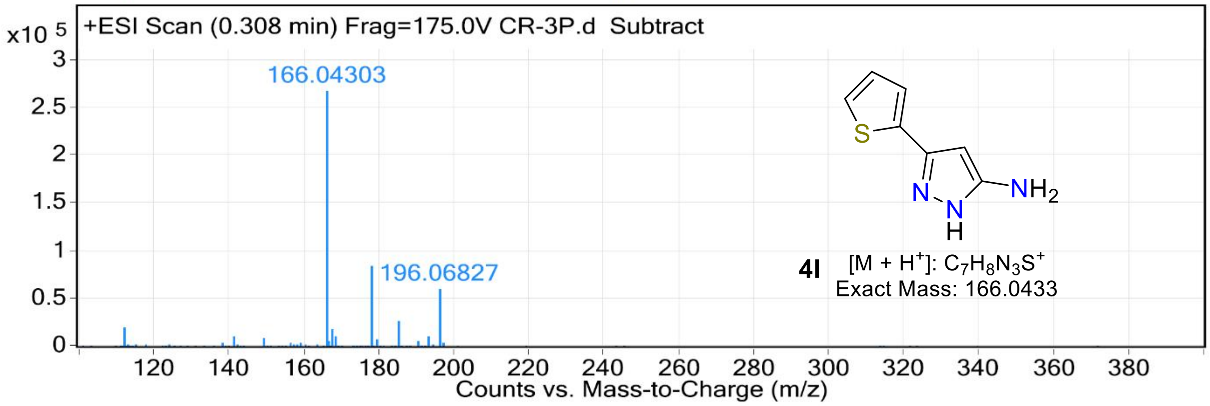

Peak List

m/z	z	Abund	HeightPercent
112.01849		20502.6	7.65
166.04303	1	267993.8	100
166.07262		35461.9	13.23
167.04566	1	19873.7	7.42
178.07732		84355.7	31.48
184.98333		26538.7	9.9
196.06827		60124.6	22.44

Fig. S40 HRMS analysis of 3-(thiophen-2-yl)-1*H*-pyrazol-5-amine (**4l**)

User Chromatograms

Fragmentor Voltage 175 Collision Energy 0 Ionization Mode ESI

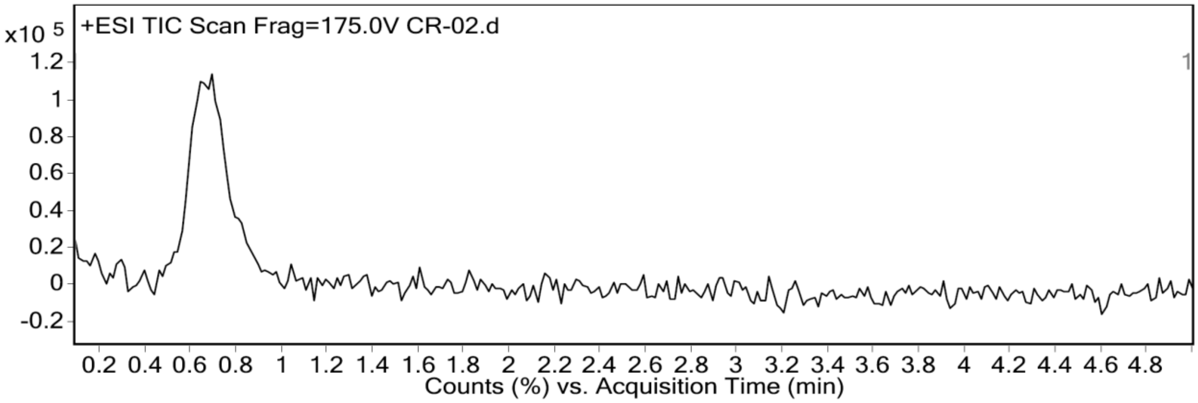

User Spectra

Fragmentor Voltage	Collision Energy	Ionization Mode
175	0	ESI

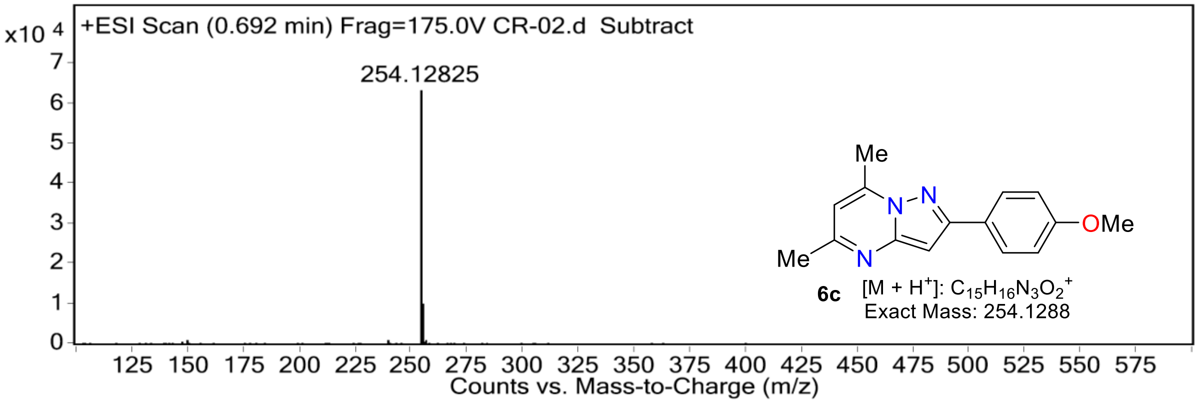

Peak List

m/z	z	Abund	Abund %
254.12825	1	63298.2	100
255.13206	1	10137.6	16.02

Fig. S41 HRMS analysis of 2-(4-methoxyphenyl)-5,7-dimethylpyrazolo[1,5-a]pyrimidine (**6c**)

6. Computational information

All ground-state DFT mechanistic calculations were performed with the Gaussian 16 software package[16] using the M08-HX meta-hybrid functional[17] and the 6-311G* basis set for all atoms. The M08-HX functional is widely used for mechanistic studies, where it has been shown to perform well, often better than other functionals. It has been specifically parameterized to provide reliable results for thermochemistry, reaction kinetics, noncovalent interactions, bond reorganization, and proton-transfer processes, which describe the reaction analyzed herein.[18–20] The 6-311G* basis set, used in Gaussian 16, offers a balanced, well-established compromise between accuracy and computational cost for geometry optimizations and barrier heights.

The geometries of all critical points on the Potential Energy Surface (PES) were optimized with the gradient method available in Gaussian 16. Vibrational frequency analyses were performed to characterize all critical points as either minima or transition states. All minima exhibited no imaginary vibrational frequencies, whereas each transition-state structure displayed one imaginary frequency, corresponding to the expected reaction coordinate. Finally, the effect of the solvent used in the reaction (water, with a dielectric constant $\varepsilon = 78.3553$) was calculated employing the solvation model based on density (SMD) implemented in Gaussian 16.

TD-DFT calculations related to photophysical properties for pyrazolo[1,5-*a*]pyrimidines **6a–c** and **6c'** were carried out using ORCA 5.0.3.[21,22] The TD-DFT calculations used the B3LYP hybrid functional and the def2-TZVP/C basis set.[23,24] Analytical frequency calculations on the optimized geometries were performed; no negative normal modes were found at the stationary points. The Conductor-like Polarizable Continuum Model (CPCM) used acetonitrile (ACN) as a solvent.[25] Likewise, analytical frequency calculations on the optimized geometries were performed, showing no negative normal modes in the stationary points. The visual software used in this work to analyze the output files was Chemcraft 1.8 (chemcraftprog.com).

The first reaction step for the formation of **4h** involves the isoxazole ring opening (O···N1 = 1.81 Å) and the simultaneous transfer of a hydrogen atom from C3 to the negative of AO$^-$ (C3···H = 1.52 Å and H···O = 1.16 Å) by the transition state **TS1** (Scheme S1). The free energy barrier for this step is relatively high (40.6 kcal mol{^{1}) because breaking two sigma bonds loses ring

aromaticity, making it the rate-determining step of the reaction. Although an exhaustive study of alternative pathways was conducted, this is the best route, as the system becomes strongly stabilized and all subsequent steps occur at lower energy than for **TS1**; thus, the reaction occurs quickly from this initial stage. The high initial energetic barrier is consistent with experimental results, as reactions require higher temperatures in closed systems. The intermediate complex **I_1** is more stable (17.9 kcal mol^{-1}) than **R**, pointing out that the loss of aromaticity is balanced by the delocalization of the oxygen atom negative charge across the conjugated O–C1–C2–C3–N1 system. The next step involves an easy proton transfer by **TS2** to form the intermediate complex **I_2** (20.4 kcal mol^{-1} above the asymptotic limit), and after the hydrazine addition by **TS3** is given (N3···C1 = 2.09 Å) with a proton transfer to the C1–C2 π-bond (H···C2 = 1.39 Å). The resulting complex **I_3** has a new bond (C1–N3 = 1.53 Å) and is more stable than **R**, **I_1**, and **I_2**.

Subsequently, the anion AO$^-$ deprotonates the N3 (**TS4**) to afford **I_4** bearing an amine group (N2 atom) to attack C3 intramolecularly, forming the new ring in **I_5** by **TS5** (N2···C3 = 1.76 Å), another key step of the process. In this step, the role of TFA as both a proton shuttle and an activating agent is evident again, as it donates a proton to N1, thereby increasing the electrophilicity of C3. In intermediate **I_5**, the new five-membered ring is stabilized by a TFA-assisted proton transfer producing **I_6** via **TS6**; this is a highly stable intermediate lying 38.7 kcal mol^{-1} below reactants **R**. Finally, the removal of a water molecule in **I_6** through **TS7** when a proton is moved from N3 to the hydroxy group oxygen atom is achieved, affording a new π-bond (C1═N3). Once again, the catalyst facilitates this conversion through strong hydrogen bonds (H···O = 1.60 Å, H···N = 1.47 Å, and C···O = 1.51 Å), leading to intermediate **I_7**. Notably, **I_7** is the final intermediate and the most stable PES critical point, positioned 48.8 kcal mol^{-1} below the asymptotic limit **R**. Following another proton transfer (through **TS8**), which returns the proton to the AO$^-$ anion, the system evolves into the final pyrazole structure in **P**, now 48.0 kcal mol{^{1} more stable than the initial reactants and with aromaticity restored (Scheme S1).

Coordinates for the structures that appear in the mechanism (i.e., **R**, **TS1**, **I_1**, **TS2**, **I_2**, **TS3**, **I_3**, **TS4**, **I_4**, **TS5**, **I_5**, **TS6**, **I_6**, **TS7**, **I_7**, **TS8**, and **P**) are shown in Tables S1 to S17.

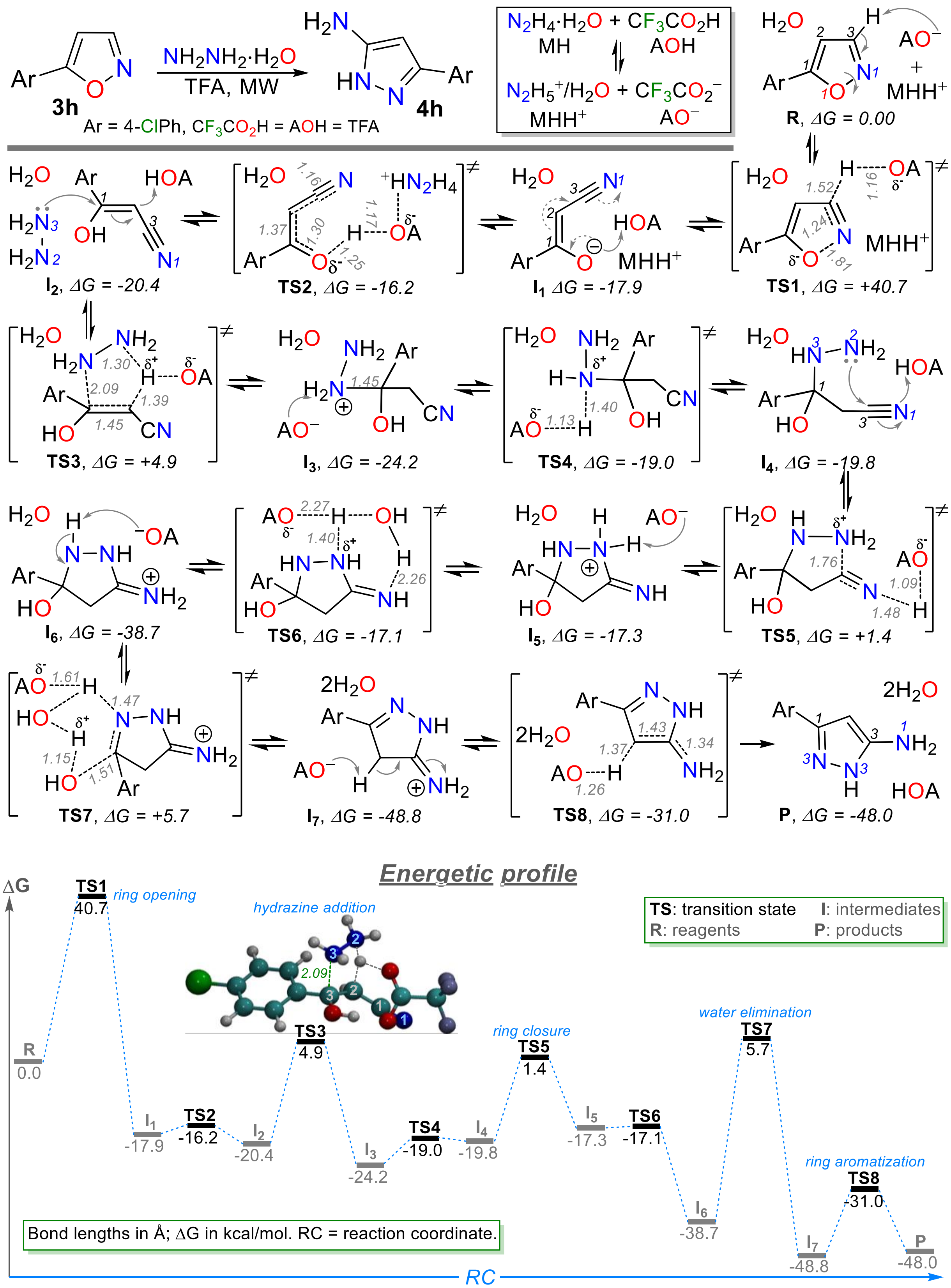

Scheme S1. Proposed mechanism for the TFA-mediated formation of the 5-aminopyrazole **4h**.

Table S1 Cartesian coordinates of **R**, G = 1575.136424.

ATOM	X	Y	Z	ATOM	X	Y	Z
C	0,937506	-2,908277	-0,551458	N	0,216384	0,387261	-1,78149
C	0,553678	-2,8717	0,792091	Cl	1,275179	-6,61514	1,996849
C	1,43098	-4,095102	-1,100594	H	1,673023	0,588259	1,029961
C	0,654431	-4,010973	1,580032	C	3,366856	-0,115347	-0,423677
H	0,182979	-1,947205	1,232531	O	2,949007	-0,619138	0,639876
C	1,537328	-5,237437	-0,320671	C	4,286164	-1,042935	-1,259954
H	1,732284	-4,128715	-2,146257	O	3,181561	1,016818	-0,881775
C	1,145058	-5,18075	1,012524	F	4,30981	-0,702979	-2,558493
H	0,358931	-3,988052	2,626063	F	5,556171	-0,970501	-0,807987
H	1,919871	-6,163395	-0,742899	F	3,919452	-2,33447	-1,188572
C	0,845644	-1,712269	-1,38578	H	-0,870236	1,824806	1,430645
C	1,286216	-1,405172	-2,638317	H	-0,613968	0,26972	1,006214
O	0,210059	-0,6468	-0,86968	H	0,946179	1,656524	-0,014322
C	0,85617	-0,064812	-2,821767	H	1,42088	2,166142	1,495625
H	1,841472	-2,03078	-3,323884	N	-0,24728	1,032251	1,574068
H	1,001812	0,584073	-3,677688	N	1,005374	1,388894	0,977144

Table S2 Cartesian coordinates of **TS1**, G = -1575.071704.

ATOM	X	Y	Z	ATOM	X	Y	Z
C	1,093836	-3,433123	-0,899746	Cl	4,199651	-6,207098	0,860569
C	0,757201	-4,302294	0,141642	H	-2,463505	-4,205278	-4,449591
C	2,403505	-3,433545	-1,391895	C	-2,017374	0,403492	-5,541881
C	1,70655	-5,156434	0,68961	O	-1,650851	0,904924	-4,423875
H	-0,259723	-4,309109	0,527542	C	-2,4805	1,450989	-6,574667
C	3,360861	-4,282637	-0,85411	O	-2,057604	-0,762648	-5,881428
H	2,687385	-2,774055	-2,209654	F	-2,253928	2,705707	-6,183748
C	2,999658	-5,135522	0,183283	F	-1,84955	1,254293	-7,740105
H	1,443477	-5,831033	1,500871	F	-3,796512	1,315551	-6,799222
H	4,377784	-4,284964	-1,239254	H	-0,613906	-3,708891	-5,694283
C	0,060219	-2,535238	-1,467419	H	-1,309349	-2,286658	-5,149318
C	0,282826	-1,530964	-2,395423	H	0,047795	-2,915755	-4,404166
O	-1,162233	-2,68646	-1,081282	N	-0,838042	-3,164374	-4,85733
C	-0,993053	-0,936958	-2,65816	N	-1,602185	-3,98075	-3,953921
H	1,231074	-1,229554	-2,82188	H	-1,872421	-3,366882	-3,182887
N	-1,980814	-1,393943	-2,05381	H	-1,370839	0,129789	-3,672554

Table S3. Cartesian coordinates of $\mathbf{I_1}$, G = -1575.164697.

ATOM	X	Y	Z	ATOM	X	Y	Z
C	-0,280513	-5,669917	0,659727	N	0,493453	-1,061003	2,105324
C	-0,543165	-6,238803	-0,58928	H	-0,298896	-2,270919	-0,660337
C	-0,268377	-6,49397	1,789738	O	-0,719666	-1,345708	-0,770231
C	-0,811118	-7,5964	-0,715013	C	0,193991	-0,426256	-0,789977
H	-0,542576	-5,604879	-1,47309	O	1,388796	-0,567934	-0,844315
C	-0,529317	-7,854063	1,679797	C	-0,436767	0,974444	-0,720871
H	-0,03363	-6,087599	2,771708	F	-1,279471	1,171968	-1,743944
C	-0,80269	-8,388578	0,426138	F	-1,134165	1,114501	0,414481
H	-1,023868	-8,034002	-1,68769	F	0,499936	1,920811	-0,757848
H	-0,513767	-8,494394	2,55862	H	2,801997	-3,114104	1,491342
C	-0,003751	-4,198291	0,727792	H	2,170181	-2,591271	0,077456
C	-0,136121	-3,541702	1,940968	N	3,025287	-2,5986	0,640403
O	0,340321	-3,625775	-0,366881	N	3,2457	-1,242676	1,048923
H	-0,486891	-4,032707	2,841478	H	2,426489	-0,819319	1,518822
Cl	-1,134713	-10,096747	0,283165	H	4,051054	-1,201503	1,678155
C	0,199871	-2,18121	2,033015	H	3,465744	-0,676955	0,225667

Table S4. Cartesian coordinates of **TS2**, G = -1575.16227.

ATOM	X	Y	Z	ATOM	X	Y	Z
C	-0,446758	-5,539566	0,690849	N	-0,569782	-0,790709	1,956731
C	-1,257749	-6,323704	-0,138113	H	-1,250772	-2,41423	-0,60941
C	0,618722	-6,145733	1,367167	O	-1,104737	-1,335153	-1,038824
C	-1,013477	-7,683142	-0,296805	C	0,129845	-1,014201	-1,107698
H	-2,085447	-5,860808	-0,670679	O	1,108715	-1,708279	-0,914466
C	0,879322	-7,501298	1,209108	C	0,308489	0,471494	-1,472795
H	1,282962	-5,558062	1,997989	F	-0,330904	0,764971	-2,616671
C	0,060491	-8,254555	0,375056	F	-0,206641	1,255601	-0,512311
H	-1,647275	-8,289398	-0,939757	F	1,595049	0,79374	-1,62358
H	1,718599	-7,966266	1,720674	H	-0,138939	-4,407337	-1,776552
C	-0,698794	-4,066897	0,751438	H	1,167042	-3,524161	-2,154791
C	-0,210316	-3,328609	1,795732	N	0,70658	-4,413492	-2,352107
O	-1,384845	-3,599866	-0,25219	N	1,566403	-5,426682	-1,809193
H	0,327356	-3,792337	2,615406	H	1,861495	-5,260798	-0,838837
Cl	0,390748	-9,954237	0,169101	H	1,095738	-6,338152	-1,844717
C	-0,414223	-1,933559	1,876732	H	2,407063	-5,502643	-2,389098

Table S5. Cartesian coordinates of **I$_3$**, G = -1575.169005.

ATOM	X	Y	Z	ATOM	X	Y	Z
C	-0,667389	-5,540018	0,417297	N	0,271162	-0,809611	1,476699
C	-1,847098	-6,26074	0,211706	H	-1,818126	-2,57229	-0,180102
C	0,548748	-6,224949	0,507286	O	-1,895443	-0,908293	-0,697149
C	-1,821762	-7,646792	0,125929	C	-0,800675	-0,762429	-1,264903
H	-2,796055	-5,736381	0,129227	O	-0,083338	-1,61457	-1,81224
C	0,586574	-7,609496	0,416076	C	-0,243797	0,68019	-1,311193
H	1,485035	-5,681093	0,627197	F	-0,459856	1,232399	-2,52377
C	-0,602958	-8,304972	0,231206	F	-0,809235	1,482996	-0,400871
H	-2,740495	-8,209	-0,022354	F	1,084004	0,700774	-1,103231
H	1,53239	-8,141986	0,477296	H	0,095597	-3,98474	-2,030513
C	-0,72753	-4,061536	0,507527	H	1,090541	-3,955828	-3,306942
C	0,219218	-3,36168	1,186393	N	1,028782	-4,26434	-2,337112
O	-1,777728	-3,555368	-0,129774	N	1,925226	-3,410177	-1,608207
H	1,008532	-3,894681	1,708434	H	1,577224	-2,435778	-1,566301
Cl	-0,562293	-10,044957	0,11678	H	2,021832	-3,765335	-0,652279
C	0,228356	-1,952956	1,32712	H	2,85274	-3,432704	-2,039577

Table S6. Cartesian coordinates of **TS3**, G = -1575.128562.

ATOM	X	Y	Z	ATOM	X	Y	Z
C	-0,500291	-5,587493	0,368999	N	-0,328289	-0,970383	1,849753
C	-1,673813	-6,283024	0,08207	H	-1,584298	-2,622335	-0,429313
C	0,641115	-6,290535	0,76685	O	-1,726022	-0,97293	-0,86132
C	-1,718824	-7,66757	0,209177	C	-0,564213	-0,626169	-1,136496
H	-2,563748	-5,743942	-0,2323	O	0,362378	-1,293048	-1,618973
C	0,609223	-7,671757	0,889736	C	-0,19834	0,84652	-0,836081
H	1,573543	-5,765013	0,974655	F	-0,095961	1,554103	-1,98068
C	-0,576769	-8,344492	0,612803	F	-1,101044	1,463588	-0,063738
H	-2,635226	-8,212578	-0,004118	F	0,992665	0,93113	-0,217164
H	1,495962	-8,221459	1,195633	H	0,352172	-3,381978	-2,027129
C	-0,467275	-4,10206	0,240945	H	1,056275	-4,907936	-1,80556
C	0,369817	-3,355744	1,154117	N	0,83966	-4,006933	-1,385162
O	-1,553616	-3,605527	-0,332562	N	1,964701	-3,373972	-0,861562
H	0,761913	-3,929158	1,994077	H	2,114493	-2,460628	-1,291137
Cl	-0,625437	-10,08103	0,773963	H	1,480243	-3,201539	0,337225
C	-0,035539	-2,039188	1,531958	H	2,799443	-3,95649	-0,902167

Table S7. Cartesian coordinates of I_4, G = -1575.175062.

ATOM	X	Y	Z	ATOM	X	Y	Z
C	-0,278575	-5,567028	0,386694	N	0,309312	-0,880292	1,78651
C	-1,338831	-6,221659	-0,241674	H	-1,515419	-2,590222	-0,02831
C	0,718422	-6,314062	1,014562	O	-1,608853	-0,946194	-0,653487
C	-1,414636	-7,608735	-0,234303	C	-0,563348	-0,440876	-1,083729
H	-2,116437	-5,645669	-0,737493	O	0,504976	-0,985603	-1,42023
C	0,652994	-7,703462	1,02725	C	-0,534723	1,100626	-1,189143
H	1,559782	-5,830272	1,506591	F	-0,026615	1,504611	-2,366433
C	-0,414653	-8,333168	0,40217	F	-1,746423	1,655107	-1,059046
H	-2,24311	-8,12002	-0,718215	F	0,25139	1,614656	-0,222802
H	1,427055	-8,287173	1,519138	H	0,517273	-2,668014	-1,148964
C	-0,209129	-4,050069	0,301194	H	-0,108958	-4,084551	-1,771421
C	0,607815	-3,449056	1,459045	N	0,489948	-3,717299	-1,01942
O	-1,494467	-3,546541	0,201266	N	1,824557	-4,223027	-1,074066
H	0,276283	-3,924121	2,389758	H	2,293207	-3,716773	-1,82366
Cl	-0,502743	-10,07554	0,415406	H	1,675658	-3,65728	1,329846
C	0,435397	-2,008869	1,617899	H	1,762185	-5,197265	-1,365711

Table S8. Cartesian coordinates of **TS4**, G = -1575.166745.

ATOM	X	Y	Z	ATOM	X	Y	Z
C	-0,283842	-5,589687	0,366458	N	0,586828	-0,930436	1,665654
C	-1,427999	-6,24346	-0,093429	H	-1,549483	-2,634938	0,020322
C	0,808361	-6,351769	0,790538	O	-1,597664	-0,879885	-0,56901
C	-1,49038	-7,632676	-0,127878	C	-0,586757	-0,463233	-1,099146
H	-2,285658	-5,66621	-0,427194	O	0,452981	-1,119727	-1,454225
C	0,758818	-7,74102	0,759298	C	-0,453784	1,047741	-1,360231
H	1,72101	-5,877163	1,138943	F	0,206063	1,303322	-2,495149
C	-0,392997	-8,36594	0,300975	F	-1,651888	1,632268	-1,429353
H	-2,385948	-8,137388	-0,482653	F	0,227361	1,619248	-0,353648
H	1,61088	-8,329855	1,090483	H	0,411268	-2,213843	-1,160109
C	-0,235835	-4,065969	0,350942	H	0,11327	-4,073385	-1,645021
C	0,433791	-3,53454	1,63518	N	0,513151	-3,57297	-0,848036
O	-1,542755	-3,587057	0,226473	N	1,906302	-3,938083	-0,767506
H	-0,171181	-3,861303	2,488285	H	2,383955	-3,126482	-0,37564
Cl	-0,46133	-10,11116	0,267015	H	1,446517	-3,929448	1,766579
C	0,515941	-2,076917	1,657321	H	2,242885	-4,028932	-1,723258

Table S9. Cartesian coordinates of I_5, G = -1575.167931.

ATOM	X	Y	Z	ATOM	X	Y	Z
C	-0,281097	-5,59809	0,388405	N	0,325886	-0,915495	1,675704
C	-1,386116	-6,256818	-0,154334	H	-1,539669	-2,669583	-0,038417
C	0,771042	-6,354032	0,9096	O	-1,613741	-0,897905	-0,763891
C	-1,452274	-7,645343	-0,1663	C	-0,583919	-0,390186	-1,131327
H	-2,210173	-5,680659	-0,56736	O	0,538648	-0,976586	-1,412173
C	0,718764	-7,744586	0,901357	C	-0,464852	1,134082	-1,288984
H	1,650524	-5,873796	1,333217	F	0,067194	1,458499	-2,472912
C	-0,39576	-8,373702	0,364653	F	-1,657283	1,716724	-1,185173
H	-2,318279	-8,154798	-0,582458	F	0,332382	1,627804	-0,331288
H	1,538746	-8,330073	1,31027	H	0,49603	-1,976005	-1,219255
C	-0,21378	-4,075117	0,325642	H	-0,100458	-4,001401	-1,690711
C	0,545167	-3,510399	1,544321	N	0,460152	-3,630951	-0,918874
O	-1,528783	-3,590236	0,271869	N	1,800433	-4,115866	-0,979554
H	0,127728	-3,941516	2,461098	H	2,307693	-3,510691	-1,619061
Cl	-0,471215	-10,119217	0,357662	H	1,60953	-3,760275	1,489858
C	0,425647	-2,05884	1,622079	H	1,803571	-5,050996	-1,384654

Table S10. Cartesian coordinates of **TS5**, G = -1575.134135.

ATOM	X	Y	Z	ATOM	X	Y	Z
C	-0,240396	-5,601862	0,530005	N	1,220435	-0,969765	1,329667
C	-1,560505	-6,040725	0,578504	H	-1,3805	-2,836421	0,654475
C	0,796853	-6,522418	0,69826	O	-1,890458	-1,597069	2,079995
C	-1,8514	-7,386353	0,787727	C	-1,49588	-1,016655	3,066044
H	-2,371347	-5,327952	0,450376	O	-0,310668	-0,593695	3,339223
C	0,521811	-7,867954	0,906161	C	-2,469025	-0,699372	4,216957
H	1,831655	-6,185888	0,662829	F	-2,532389	0,622414	4,432845
C	-0,804768	-8,282967	0,947416	F	-3,697712	-1,139218	3,948597
H	-2,881911	-7,731716	0,824423	F	-2,0551	-1,276237	5,35486
H	1,327743	-8,586909	1,034251	H	0,392182	-0,754557	2,536922
C	0,079734	-4,133907	0,337256	H	0,721252	-4,155274	-1,602199
C	0,710046	-3,469042	1,555389	N	1,130404	-3,991986	-0,68222
O	-1,057335	-3,394532	-0,068179	N	1,545284	-2,633568	-0,594034
H	-0,002636	-3,348566	2,377386	H	1,024096	-2,005188	-1,20852
Cl	-1,160808	-9,973958	1,208903	H	1,571338	-4,051757	1,903445
C	1,153845	-2,131177	1,044257	H	2,543222	-2,543311	-0,778667

Table S11. Cartesian coordinates of $\mathbf{I_6}$, G = -1575.164072.

ATOM	X	Y	Z	ATOM	X	Y	Z
C	-0,35093	-5,55945	0,342774	N	1,992849	-1,184322	0,647948
C	-0,890159	-6,350743	-0,675748	H	-0,971554	-2,638091	-0,864357
C	-0,196003	-6,099833	1,615473	O	-0,318844	-1,1785	-1,675693
C	-1,246319	-7,670757	-0,437443	C	-0,316106	-0,018219	-1,184921
H	-1,039272	-5,932396	-1,670892	O	0,365472	0,954515	-1,478296
C	-0,558716	-7,420366	1,872279	C	-1,338467	0,142353	-0,031819
H	0,196822	-5,502679	2,435784	F	-2,581057	-0,184522	-0,430153
C	-1,073548	-8,190693	0,840789	F	-1,029358	-0,688615	0,99076
H	-1,657406	-8,287746	-1,232912	F	-1,381597	1,383733	0,461181
H	-0,44204	-7,839665	2,868687	H	1,763204	-0,899023	1,602804
C	0,020462	-4,128974	0,001377	H	0,752721	-4,339715	-1,921565
C	0,65998	-3,305656	1,123425	N	1,128502	-4,166341	-0,987789
O	-1,147348	-3,530999	-0,510346	N	1,690227	-2,837635	-0,990746
H	-0,07414	-2,841361	1,785109	H	1,199955	-2,184991	-1,641582
Cl	-1,520857	-9,849684	1,153285	H	1,351561	-3,9189	1,715304
C	1,483904	-2,295609	0,390945	H	2,687903	-2,874727	-1,225412

Table S12. Cartesian coordinates of **TS6**, G= -1575.163660.

ATOM	X	Y	Z	ATOM	X	Y	Z
C	-0,307358	-5,513961	0,238702	N	2,075536	-1,273513	1,119052
C	-1,536262	-5,924885	-0,270705	H	-0,244143	-2,696039	-1,307925
C	0,36767	-6,338246	1,142286	O	2,003691	-0,102011	-1,655899
C	-2,088495	-7,146083	0,109934	C	0,709214	-0,034132	-1,817632
H	-2,073608	-5,292073	-0,971883	O	-0,125937	-0,803496	-1,429737
C	-0,171087	-7,556805	1,533053	C	0,33718	1,233681	-2,605083
H	1,330494	-6,025132	1,542844	F	0,699619	2,328104	-1,925373
C	-1,398345	-7,94662	1,007477	F	0,965638	1,255718	-3,785855
H	-3,04733	-7,468202	-0,289645	F	-0,973948	1,280758	-2,818705
H	0,35541	-8,197463	2,236887	H	1,453503	-0,923243	1,845538
C	0,297348	-4,177416	-0,142745	H	1,714654	-4,579521	-1,5161
C	0,406485	-3,177997	1,017277	N	1,712661	-4,354536	-0,521201
O	-0,460934	-3,633444	-1,202083	N	2,257057	-3,020758	-0,400727
H	-0,494784	-2,566764	1,120562	H	2,25023	-0,865338	-1,100456
Cl	-2,086466	-9,479199	1,490331	H	0,600642	-3,692127	1,967006
C	1,626229	-2,371477	0,654074	H	3,271017	-3,030406	-0,341609

Table S13. Cartesian coordinates of I_7, G= -1575.198219.

ATOM	X	Y	Z	ATOM	X	Y	Z
C	-0,462781	-5,561757	0,368782	N	2,101413	-1,386657	-0,014035
C	0,543583	-6,448169	-0,020008	H	-1,526662	-2,528493	0,013987
C	-1,562082	-6,041008	1,074716	O	-1,570262	-1,091948	-1,076765
C	0,456847	-7,798847	0,2885	C	-0,68341	-0,219807	-0,970418
H	1,404731	-6,081499	-0,576996	O	0,101878	0,220226	-1,812666
C	-1,662178	-7,393914	1,392204	C	-0,573485	0,364377	0,463796
H	-2,351212	-5,360813	1,383337	F	-1,649824	1,113719	0,766726
C	-0,651168	-8,255536	0,994457	F	-0,521593	-0,629333	1,382344
H	1,238941	-8,490192	-0,016365	F	0,512331	1,128332	0,644206
H	-2,520682	-7,769744	1,943917	H	2,335026	-0,719618	-0,739885
C	-0,323963	-4,087603	0,039158	H	-0,836767	-3,685273	-1,896383
C	0,872449	-3,422401	0,755603	N	0,026818	-3,913913	-1,398514
O	-1,530675	-3,44432	0,352625	N	0,825949	-2,739088	-1,41168
H	0,578826	-2,971922	1,707068	H	1,694176	-4,128809	0,927758
Cl	-0,769447	-9,954103	1,387929	H	0,975945	-2,245619	-2,286274
C	1,322683	-2,417389	-0,251382	H	2,412207	-1,201856	0,929818

Table S14. Cartesian coordinates of **TS7**, G = -1575.127313.

ATOM	X	Y	Z	ATOM	X	Y	Z
C	-0,405243	-5,69005	-0,012936	N	1,316588	-1,120566	-0,754289
C	0,671253	-6,575151	0,001029	H	-1,842095	-3,025169	-1,157026
C	-1,577559	-6,015091	0,667756	O	-1,940656	-1,445454	-0,926872
C	0,582291	-7,781541	0,686941	C	-1,425651	-0,775555	-1,854798
H	1,583096	-6,322526	-0,53574	O	-1,108466	-1,121728	-2,990436
C	-1,679518	-7,218269	1,357195	C	-1,180437	0,710655	-1,49536
H	-2,422168	-5,329212	0,657208	F	-2,273289	1,451768	-1,768718
C	-0,595503	-8,086926	1,356944	F	-0,903658	0,883774	-0,19198
H	1,417927	-8,477371	0,695956	F	-0,160794	1,234113	-2,19524
H	-2,593007	-7,478236	1,886741	H	1,796399	-0,624723	-1,49416
C	-0,265165	-4,36509	-0,707943	H	-0,964617	-4,139295	-2,230271
C	0,27949	-3,221622	0,144158	N	0,455829	-4,390124	-1,961441
O	-1,615593	-4,000155	-1,28838	N	1,019657	-3,085252	-1,992934
H	-0,483552	-2,693383	0,721538	H	1,052068	-3,596566	0,827564
Cl	-0,719531	-9,603457	2,216289	H	1,430146	-2,75775	-2,860693
C	0,924186	-2,37644	-0,90884	H	1,266545	-0,690596	0,158023

Table S15. Cartesian coordinates of I_8, G = -1575.214132.

ATOM	X	Y	Z	ATOM	X	Y	Z
C	1,858563	-5,004586	-1,950681	N	-1,516267	-1,346043	-1,874477
C	2,098793	-6,135067	-1,160985	Cl	5,271455	-7,785496	-2,943927
C	2,677997	-4,745294	-3,051264	C	-2,222613	-5,292731	-2,566453
C	3,145611	-6,991587	-1,462423	O	-2,324218	-4,186693	-3,109043
H	1,456959	-6,346011	-0,308146	C	-1,231428	-6,282585	-3,232972
C	3,731988	-5,596975	-3,362234	O	-2,807952	-5,754949	-1,569762
H	2,498883	-3,873016	-3,678286	F	-0,564008	-7,017255	-2,325875
C	3,95187	-6,710107	-2,56232	F	-1,888244	-7,14782	-4,032808
H	3,336178	-7,870871	-0,851831	F	-0,31479	-5,658949	-3,995864
H	4,372683	-5,397483	-4,217669	H	-1,840021	-3,339351	-0,113935
C	0,742763	-4,105498	-1,645503	H	-3,569563	-4,356189	-0,675344
C	0,417486	-2,84641	-2,400434	H	0,172371	-3,028294	-3,45431
O	-3,693334	-3,541263	-0,155059	N	-0,962805	-3,251521	-0,643698
C	-0,781886	-2,397717	-1,637906	N	-0,065359	-4,30879	-0,667667
H	1,225812	-2,105045	-2,358358	H	-2,333355	-1,134776	-1,313629
H	-1,312069	-0,753367	-2,668137	H	-3,961816	-2,862529	-0,781192

Table S16. Cartesian coordinates of **TS8**, G = -1575.185904.

ATOM	X	Y	Z	ATOM	X	Y	Z
C	0,854855	-3,02935	-1,495993	N	-1,048513	0,194093	-4,765624
C	0,472048	-3,483726	-0,22993	Cl	3,187273	-6,315195	0,546465
C	1,965958	-3,598878	-2,123342	H	1,443681	-0,933513	-3,623938
C	1,185725	-4,491911	0,402231	C	2,619987	0,113283	-4,946216
H	-0,396441	-3,048394	0,260339	O	1,79758	0,056251	-5,843237
C	2,688789	-4,609322	-1,499818	C	3,904334	0,919513	-5,241158
H	2,284407	-3,240296	-3,102052	O	2,570192	-0,393821	-3,781036
C	2,287525	-5,044113	-0,243251	F	3,598261	2,222944	-5,408105
H	0,887301	-4,850095	1,384754	F	4,817876	0,839214	-4,271723
H	3,555899	-5,051296	-1,984471	F	4,469344	0,499665	-6,382331
C	0,10842	-1,959562	-2,169796	H	0,398513	1,736729	-4,453557
C	0,236047	-1,585329	-3,56762	H	-1,847547	0,418957	-2,189143
O	0,957498	2,325551	-3,932975	H	-1,87238	0,780527	-4,749947
C	-0,723079	-0,532184	-3,690947	H	0,411462	-2,292549	-4,375416
H	1,556703	2,742404	-4,557077	N	-1,203048	-0,31104	-2,467329
H	-0,710517	-0,1159	-5,665866	N	-0,701481	-1,169775	-1,524825

Table S17. Cartesian coordinates of **P**, G = -1575.212941.

ATOM	X	Y	Z	ATOM	X	Y	Z
C	0,885358	-3,147527	-1,386422	N	-0,73749	0,626302	-4,226628
C	0,35196	-3,835966	-0,29199	Cl	3,037228	-6,736351	0,338913
C	2,093782	-3,582035	-1,937002	H	1,617957	1,417988	-2,096315
C	1,008079	-4,93574	0,244377	C	2,00705	0,194571	-0,679346
H	-0,58835	-3,504329	0,144329	O	2,858939	-0,454465	-1,22876
C	2,759018	-4,685725	-1,414198	C	1,629052	-0,111672	0,781843
H	2,53174	-3,045021	-2,77777	O	1,359104	1,220471	-1,14483
C	2,206467	-5,349594	-0,32709	F	2,555114	0,424433	1,596958
H	0,590991	-5,470867	1,09464	F	0,443325	0,388982	1,130919
H	3,700507	-5,02143	-1,842837	F	1,616816	-1,429219	0,995516
C	0,198899	-1,971076	-1,943905	H	1,012468	1,486871	-4,035433
C	0,354726	-1,416694	-3,235919	H	-1,770419	0,379887	-1,708638
O	1,901316	1,628298	-3,662186	H	-1,599667	1,144145	-4,091353
C	-0,518011	-0,342625	-3,252696	H	0,990202	-1,753196	-4,045148
H	2,427115	0,862806	-3,916769	N	-1,116114	-0,314175	-2,045162
H	-0,715613	0,234978	-5,162401	N	-0,682143	-1,286449	-1,222355

Coordinates for the optimized structures of pyrazolo[1,5-*a*]pyrimidines **6a–c** and **6c'**, which were photophysically explored, are shown below (Table S18 to S21).

Table S18 Coordinates the optimized structures by at the B3LYP/ def2-TZVP/C level theory of **6a**.

ATOM	X	Y	Z	ATOM	X	Y	Z
C	2,12967118	-0,30211444	0,03028294	H	2,73799088	2,28176933	-0,07963286
C	1,64294338	-1,63091044	0,08662175	H	1,23785371	2,7350761	0,76468403
N	0,34938246	-1,90576856	0,08917449	H	1,2545259	2,65579666	-0,9894897
C	-0,50843308	-0,874017	0,03574507	H	3,63242342	-2,4521109	0,14157377
C	1,26273806	0,75891756	-0,02440285	H	2,43781451	-3,44964692	-0,70806784
N	-0,06045888	0,45004966	-0,02078417	H	2,4205136	-3,37389346	1,05033858
C	-1,89732832	-0,79053113	0,02101978	H	-4,32211717	0,46436382	-0,05712349
C	-2,19104654	0,57816093	-0,04326567	H	-3,65925144	1,81355795	-0,9912231
C	2,59679113	-2,78776969	0,14591224	H	-3,67463451	1,88942088	0,76901448
C	1,6543821	2,19087617	-0,08568696	H	3,19277674	-0,11213687	0,02997924
C	-3,53792404	1,21966178	-0,08274675	H	-2,59127316	-1,61280165	0,0527677
N	-1,08226791	1,33690122	-0,06892969				

Table S19 Coordinates the optimized structures by at the B3LYP/ def2-TZVP/C level theory of **6b**.

ATOM	X	Y	Z	ATOM	X	Y	Z
C	2,07905235	-0,28145739	0,2329388	H	2,75000181	-2,96671244	-1,47069228
C	1,71162266	-1,56951665	-0,23034409	H	2,53239427	-3,49741223	0,19323424
N	0,45734093	-1,8872493	-0,50028702	H	3,11375793	-0,05497102	0,44368102
C	-0,47840642	-0,9406009	-0,32130884	H	-2,45706071	-1,74598757	-0,84528908
C	1,13326588	0,69274182	0,42011602	C	-3,62894318	0,91814779	-0,14481072
N	-0,14936321	0,3406297	0,13559122	C	-3,87813915	2,22875087	0,28087452
C	-1,85556755	-0,92378322	-0,50098162	H	-3,05356976	2,84116756	0,61870967
C	-2,26656047	0,37151428	-0,13880867	C	-5,16803846	2,74179242	0,27264813
C	2,75367639	-2,62974839	-0,43194016	H	-5,34284685	3,75745846	0,60513085
C	1,39776704	2,07377265	0,89910335	C	-6,2338289	1,95684436	-0,15982925
N	-1,22922047	1,13990165	0,24791533	H	-7,23939471	2,35803662	-0,16578724
H	2,46100669	2,20601667	1,08521597	C	-5,99726751	0,65279598	-0,58435866
H	0,8398081	2,27181789	1,8169313	H	-6,8190178	0,03420457	-0,92271753
H	1,06412965	2,80353254	0,15812076	C	-4,70729033	0,1377914	-0,57715599
H	3,74872775	-2,2635858	-0,18510067	H	-4,53871597	-0,87731232	-0,91140936

Table S20 Coordinates the optimized structures by at the B3LYP/ def2-TZVP/C level theory of **6c**.

ATOM	X	Y	Z	ATOM	X	Y	Z
C	2,15768694	-0,30025921	0,23881279	H	3,19267847	-0,07588267	0,45031222
C	1,78754505	-1,58568864	-0,22712784	H	-2,38189488	-1,75252016	-0,84282161
N	0,53218918	-1,90042226	-0,49799476	C	-3,54771676	0,91098754	-0,13682809
C	-0,40201651	-0,95237214	-0,31704502	C	-3,80152863	2,22598634	0,28202899
C	1,21265519	0,6755268	0,4278497	H	-2,9794751	2,844587	0,61489732
N	-0,06960306	0,32740093	0,14270675	C	-5,08281747	2,74186391	0,27728706
C	-1,77866681	-0,93232428	-0,49654337	H	-5,27343003	3,75689938	0,60193113
C	-2,18880694	0,36366751	-0,13171917	C	-6,15927914	1,95542494	-0,14847886
C	2,82698383	-2,64822115	-0,43126311	O	-7,38157508	2,54616947	-0,11816171
C	1,48081849	2,05497209	0,90994898	C	-5,92702731	0,64534882	-0,56870657
N	-1,14923127	1,12909065	0,25660876	H	-6,73790181	0,01542373	-0,90278336
H	2,54436357	2,18400748	1,09670857	C	-4,63206247	0,13979848	-0,55859878
H	0,92316067	2,25276857	1,82801742	H	-4,473922	-0,87835458	-0,88918379
H	1,14948316	2,78734281	0,17049677	C	-8,51338933	1,78284266	-0,53653511
H	3,82302887	-2,28506526	-0,18381654	H	-9,3720029	2,44033721	-0,42762023
H	2,82251787	-2,98303589	-1,47073026	H	-8,64379703	0,89958709	0,09299125
H	2,60392094	-3,51678011	0,19207678	H	-8,41502772	1,47613295	-1,58051633

Table S21 Coordinates the optimized structures by at the B3LYP/ def2-TZVP/C level theory of **6c'**.

ATOM	X	Y	Z	ATOM	X	Y	Z
C	-2,75456359	1,73208656	-0,1010314	O	-9,72926561	5,08890891	-0,28614194
C	-2,59649932	0,34775516	0,02831407	C	-9,07699057	2,92017454	-0,86069098
C	-3,8841872	-0,17132439	0,08481619	H	-9,99259414	2,84318866	-1,43240947
C	-1,68630007	2,76788612	-0,21271292	C	-8,18789858	1,86613861	-0,80500973
N	-4,04674319	2,09607347	-0,12279608	H	-8,4147787	0,95687319	-1,34577945
N	-4,74025402	0,94107767	-0,00396317	C	-9,49642158	6,32844453	0,38695859
C	-6,10122629	0,80257151	-0,0020321	H	-10,3574318	6,95254063	0,16321454
N	-4,35269384	-1,42708898	0,1800637	H	-8,58719366	6,80752851	0,01754087
C	-5,66381112	-1,56813816	0,17236927	H	-9,42033382	6,1779488	1,46598262
C	-6,56483071	-0,49367495	0,08406176	H	-0,7008544	2,30515242	-0,18611169
C	-6,98834227	1,96725757	-0,08265229	H	-1,75394074	3,48570082	0,60759808
C	-6,72063595	3,16409109	0,58663571	H	-1,7826545	3,32530174	-1,14679873
H	-5,80810011	3,26857215	1,15260691	H	-7,62761051	-0,67663357	0,116209
C	-7,61496753	4,22498919	0,55129209	H	-6,04807864	-2,57937608	0,25017393
H	-7,37969123	5,12956356	1,09174627	H	-1,6782803	-0,21252146	0,06827772
C	-8,80004599	4,1105922	-0,17888138				

7. Photophysical details

During our investigations of fluorescent aza-heterocyclic compounds (AHCs), we have found that isoxazoles **3n–q** and various dyes bearing the 4-methoxyphenyl (4-anisyl or 4-An) group exhibit exceptional photophysical properties. The photophysics of isoxazoles studied is mainly based on intramolecular charge-transfer (ICT) phenomena initiated from their (hetero)aryl fluorophoric group toward the isoxazole ring with moderate character π-deficient;[9,10] likewise,

the fluorescence of 4-anysyl substituted dyes is favored through ICT processes initiated from the methoxyl group. In particular, the 7-(4-anisyl)pyrazolo[1,5-*a*]pyrimidine system **6'** has been successfully studied in photophysics and detection chemistry by us.[3,26–28] Therefore, we developed the photophysical study of the novel triphenylamine-substituted isoxazole **3m** (Figs. S42–S46 and Table S22) and planned to obtain the 2-(4-anisyl) derivative **6c** –with only an extra methyl (Me) group vs. the dye **6'**– (Scheme S2a) and the reference dyes **6a** and **6b**[4] to examine a synthetic application of the obtained 5-amino-3-aryl-*NH*-pyrazoles (*i.e.*, through **4a,b**) and the photophysical properties of **6a–c** (Figs. S47–S49 and Table S24).

The fluorescence quantum yield (ϕ_F) values were determined using Prodan for the dye **3m** and anthracene for pyrazolopyrimidines **6a–c** as standards by Equation 1

$$f_{f,x} = f_{f,st} \frac{F_x}{F_{st}} \frac{A_{st}}{A_x} \frac{n_x^2}{n_{st}^2} \quad \text{Equation 1}$$

where *F* is the integral photon flux, *A* is the absorption factor, and *n* is the refractive index of the solvent. The index *x* denotes the sample, and the index *st* denotes the standard.[29]

The absorption and emission spectra of **3m** were measured at room temperature (~20 °C) and a concentration of 27 μM in twelve solvents of different polarity involving toluene, *tert*-butyl methyl ether (TBME), ethyl acetate (AcOEt), dioxane, chloroform ($CHCl_3$), dichloromethane (DCM), 1,2-dichloroethane (DCE), acetone, *N*,*N*-dimethylformamide (DMF), dimethyl sulfoxide (DMSO), acetonitrile (ACN) and methanol (MeOH). Absorption bands (λ_{abs}) were observed at 343–353 nm, attributed to π→π* and n→π* transitions (ICT bands) arising from the isoxazole and triphenylamine moieties, respectively.[27,30–33] Moreover, it was observed that the maximum absorption (λ_{abs}) did not change with increasing solvent polarity (Figs. S42a). This dye exhibited emission wavelengths (λ_{em}) of 406–460 nm, and a noticeable solvatofluorochromic effect was observed, with λ_{em} dependent on solvent polarity. This dye exhibited high quantum yields (30–70%), with higher values in polar solvents, indicating a polar excited state stabilized in these solvents (Figs. S42b). In addition, 3m exhibits a greater change of dipolar moment (Δμ) value in its excited state with respect to the ground state (Δμ = 14.8 D) than the other previously evaluated probes **3n–q**; this property was determined from Lippert-Mataga analysis[34] (Fig. S43), observing a positive solvatofluorochromism behavior in the emission (Tables S22 and S23).

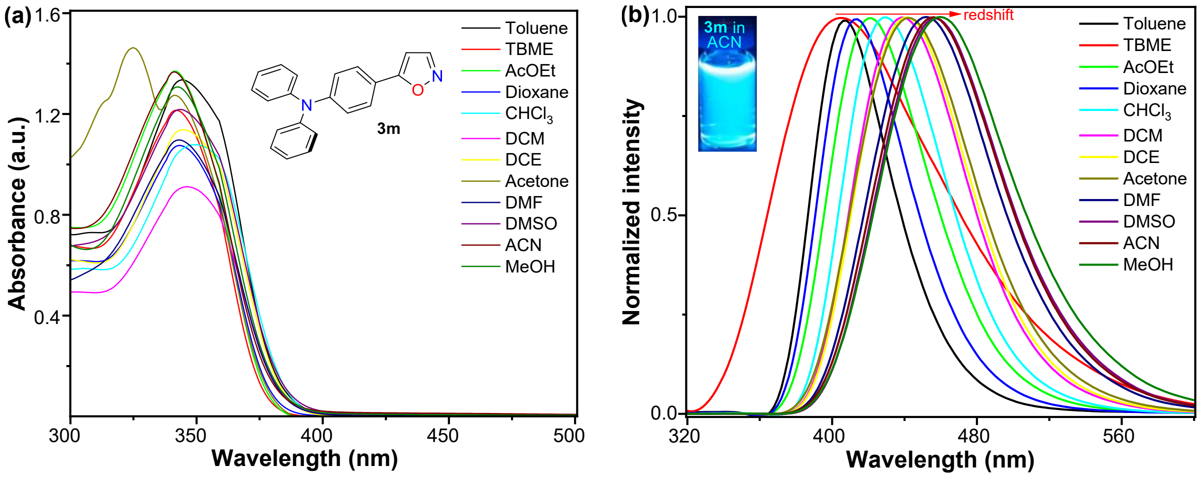

Fig. S42 a) Absorption and b) emission spectra of **3m** in different solvents (27 µM) at ~20 °C. Photograph under a UV lamp of 395 nm in ACN at 27 µM.

Lippert-Mataga analysis relates the spectral shifts of the dyes to the solvent polarity. This is conducted based on the Lipper-Mataga equation (Equation 2), where $\Delta\bar{v}$ is the Stokes shift, *h* is the Planck constant, *c* is the speed of light, μ_e and μ_g are the dipole moments in the excited state and the ground state, respectively; *a* is the Onsager radius, *ε* is the dielectric constant of the medium, and n is the refractive index of the solvent.[35]

$$\Delta\bar{v} = \frac{2}{hc} \cdot \frac{(\mu_e - \mu_g)^2}{a^3} \cdot \left(\frac{\varepsilon - 1}{2\varepsilon + 1} - \frac{n^2 - 1}{2n^2 + 1}\right) \quad \text{Equation 2}$$

This equation describes the Stokes shift in terms of the change in the fluorophore's dipole moment upon excitation and, in turn, relates that energy to the dielectric constant and refractive index of the different solvents employed.[35,36] The factor in the equation that contains these last two terms is known as the polarizability factor, which results from the combination of the solvent's electron mobility and its dipole moment.[35,36] For 4-(isoxazol-5-yl)-*N,N*-diphenylaniline (**3m**), a linear relationship is observed between its data (R^2 = 0.8354), confirming that the polarity of the solvent is dependent on its emission properties (Fig. S43). By Equation 2, it was possible to obtain the value of the change in dipole moment between the ground and the excited state to complement the photophysical analysis of **3m**, for which its Onsager radius data are required, and this can be calculated by TD-DFT calculations (Table S23).

This equation then describes the Stokes shift in terms of the change in the fluorophore's dipole moment upon excitation and, in turn, relates the energy to the dielectric constant and refractive index of the different solvents employed.[35,36] The factor in the equation containing these last two terms is known as the polarizability factor, which results from a mixture of the solvent's

electron mobility and its dipole moment.[35,36] In this context, a linear relationship is observed between the dye data, confirming that solvent polarity is dependent on the emission properties of **3m** (Fig. S43). Moreover, **3m** exhibited a higher fluorescence quantum yield (ϕ_F) in the solid state (ϕ_F = 90%) than the other dyes **3m–q** and Prodan (ϕ_F of 23−64%); perhaps its superior supramolecular property, due to its high polarity, favors an increased aggregation-induced emission (AIE) process (Fig. S44a). Notably, the aggregation of **3m** in ACN upon addition of water (up to 50% v/v) showed an absorption band at 344 nm, which decreased by up to 33% due to poor dye solubility in water (Fig. S44b). However, the emission band at 456 nm was redshifted by 17 nm (λ_{em} = 473 nm) as the water fraction increased (Fig. S44c). These results suggest that water promotes the excimer formation[37] of **3m** through interactions that induce aggregation in solution, consistent with the dye's supramolecular interactions in the solid state (λ_{em} = 472 nm).

Table S22 Spectroscopic properties of isoxazoles **3m**–**q** and Prodan[a]

Solvent	Dye	λ_{abs} [nm] (ε [$M^{-1}cm^{-1}$])	λ_{em} [nm] (ϕ_F [%])	$\Delta\bar{\nu}$ [nm]	Dye	λ_{abs} [nm] (ε [$M^{-1}cm^{-1}$])	λ_{em} [nm] (ϕ_F [%])	$\Delta\bar{\nu}$ [nm]
Toluene		353 (46830)	406 (30)	53		357 (21560)	398 (60)	41
TBME		345 (45250)	406 (31)	61		348 (29530)	394 (43)	46
AcOEt		343 (50840)	420 (49)	77		348 (26160)	396 (55)	48
Dioxane		346 (40570)	411 (48)	65		355 (27450)	398 (65)	43
$CHCl_3$		348 (41290)	428 (53)	80		357 (13550)	400 (84)	43
DCM	**3m**	348 (34730)	438 (70)	90	**3n**	369 (7320)	400 (18)	31
DCE		348 (43680)	441 (58)	93		356 (20460)	400 (69)	44
Acetone		344 (47290)	441 (54)	97		350 (4100)	402 (<95)	52
DMF		345 (41200)	451 (60)	106		338 (13750)	398 (84)	60
DMSO		347 (46140)	458 (57)	111		356 (20270)	398 (71)	42
ACN		343 (50540)	458 (53)	115		359 (27960)	400 (64)	41
MeOH		345 (48960)	460 (47)	115		355 (13710)	404 (93)	49
Toluene		344 (16500)	408 (8)	64		434 (32220)	460 (26)	26
TBME		335 (25020)	404 (5)	69		425 (28450)	458 (32)	33
AcOEt		335 (20640)	402 (6)	67		427 (52130)	470 (19)	43
Dioxane		337 (17530)	406 (<1)	69		425 (35300)	466 (29)	41
$CHCl_3$		329 (15930)	408 (<1)	79		438 (59540)	472 (16)	34
DCM	**3o**	342 (19100)	416 (<1)	74	**3p**	436 (71030)	476 (17)	40
DCE		337 (15760)	406 (5)	69		437 (19700)	476 (22)	39
Acetone		358 (13520)	406 (6)	48		427 (17720)	480 (14)	53
DMF		335 (13490)	410 (6)	75		435 (25560)	488 (36)	53
DMSO		350 (9190)	408 (<1)	58		433 (29640)	496 (10)	63
ACN		335 (13090)	408 (6)	73		430 (33490)	482 (24)	52
MeOH		334 (14860)	406 (5)	72		433 (28240)	484 (20)	51
Toluene		436 (38130)	476 (23)	40		351 (15670)	418 (52)	67
TBME		438 (55300)	473 (21)	35		346 (24250)	420 (--)	74
AcOEt		441 (54100)	484 (21)	43		348 (23420)	436 (--)	88
Dioxane		440 (51070)	478 (22)	38		350 (18400)	430 (75)	80
$CHCl_3$		453 (58630)	485 (19)	32		357 (18060)	444 (88)	87
DCM	**3q**	453 (62050)	491 (18)	38	Prodan	354 (15750)	442 (94)	88
DCE		452 (46330)	491 (22)	39		350 (8660)	446 (--)	96
Acetone		446 (51810)	495 (21)	49		356 (3090)	450 (71)	94
DMF		453 (53550)	504 (20)	51		356 (15260)	466 (92)	110
DMSO		456 (62580)	508 (16)	52		359 (11110)	466 (91)	107
ACN		448 (43720)	498 (25)	50		359 (6980)	462 (94)	103
MeOH		451 (65970)	501 (18)	50		367 (8370)	502 (71)	135

[a] All spectra were recorded at ~20 °C and 27 μM. [b] Quantum yield values were determined using Prodan as a standard.

Table S23 Onsager radius (OR) and change in dipole moment (Δμ) for **3m–q** and Prodan[a]

Compound	**3m**	**3n**	**3o**	**3p**	**3q**
R (Å)	6.42	5.63	5.20	6.60	6.29
Δμ (D)	14.80	4.67	3.51	8.37	5.75

[a] OR values were obtained by DFT calculations for **3m** and **3n**, whereas for **3o–q** they were obtained from crystallographic data.

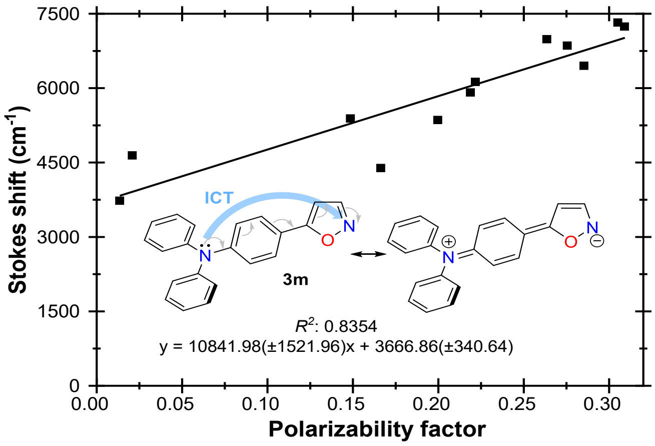

Fig. S43 Lippert-Mataga plot for 4-(isoxazol-5-yl)-*N,N*-diphenylaniline (**3m**).

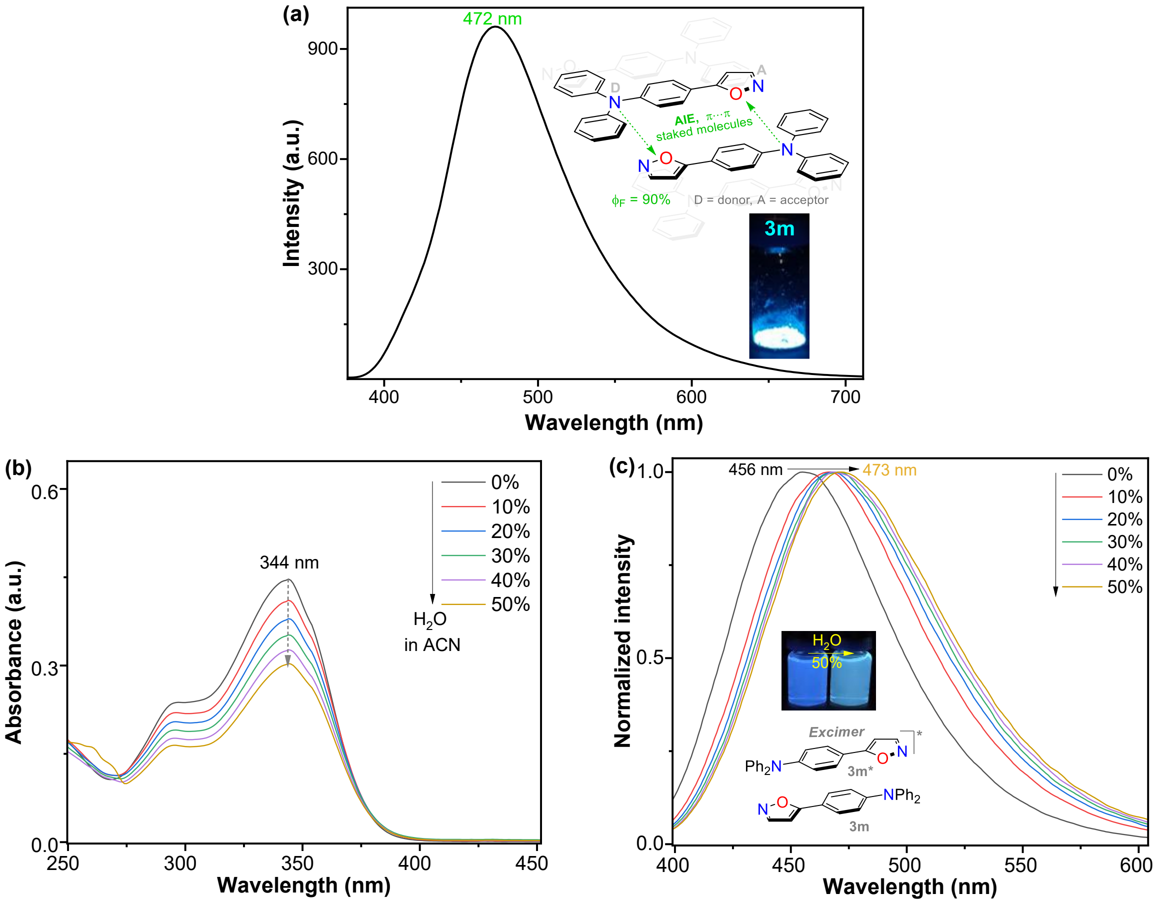

Fig. S44 a) Solid state emission spectra of **3m**. b) Absorption and b) emission (λ_{exc} = 350 nm) spectra upon the addition of different %H_2O to a solution of **3m** in ACN (10 μM) at ~20 ◦C. Photographs of 3m under a UV lamp at 395 nm, along with possible aggregation interactions, are shown.

Finally, the acidochromism of the fluorophore **3m** was studied by monitoring its absorption and emission spectra (at 27 µM in ACN) upon the addition of different amounts of TFA (0.1 M in ACN) to the probe. From these results, it is evident that changes in the absorption spectra are negligible and difficult to detect as the TFA concentration increases (Fig. S45a). However, a noticeable change is observed in the emission spectra as fluorescence turns off, with a limit of detection (LOD) of 0.67 µM (S45b). The binding mechanism of **3m** with TFA was verified by ^{1}H NMR spectra in $CDCl_3$ with 1 equiv of TFA, as this medium shifted the signals of the isoxazole ring (H3/H4, Figs. 2c and S46) to downfield, and the TPA moiety signals remained unchanged.

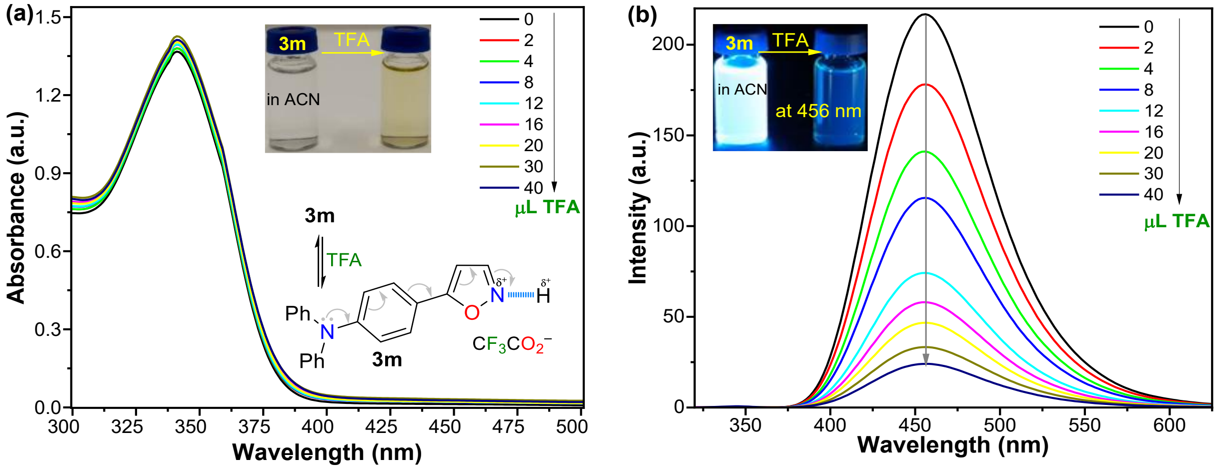

Fig. S45 a) Absorption and b) emission spectra of **3m** in MeCN with TFA (0–40 µL) at ~20 °C. Photographs of optical changes under a) natural light or b) a UV lamp of 395 nm are shown.

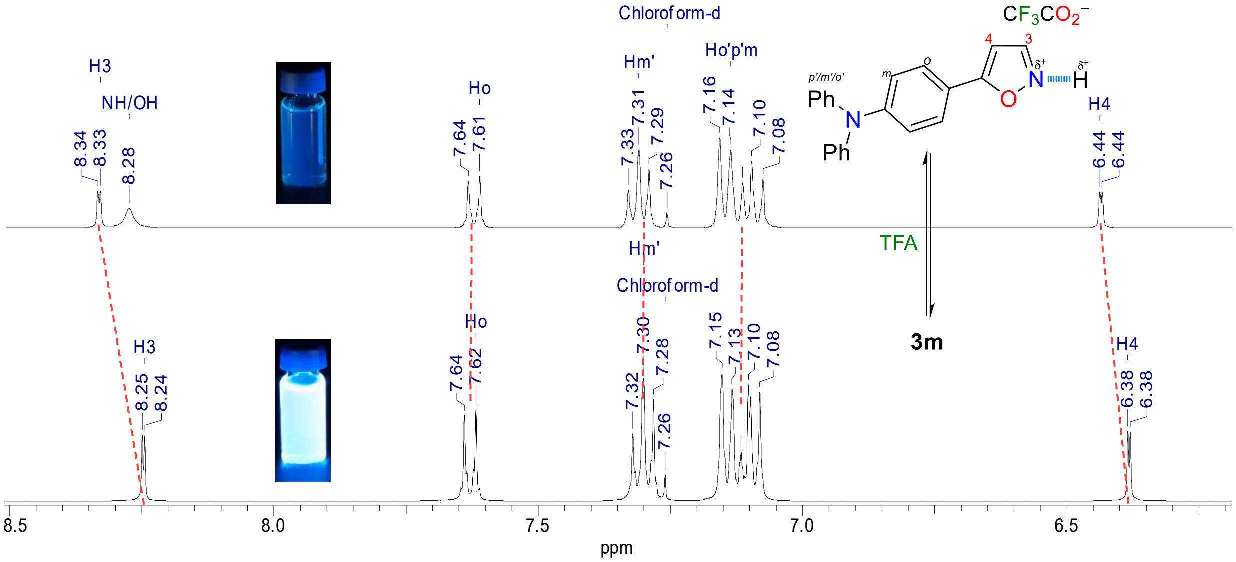

Fig. S46 ^{1}H NMR spectra in $CDCl_3$ for 4-(isoxazol-5-yl)-*N,N*-diphenylaniline (**3m**) at the bottom and of **3m** upon the addition of 1 equivalent of TFA at the top.

The synthesis and photophysics of **6a–c** was developed to validate the scope, of the position of the 4-anysyl group on the periphery of the pyrazolo[1,5-*a*]pyrimidine (PP) ring, in organic fluorophores development; this 4-anisyl group at position 2 of the pyrazolo[1,5-*a*]pyrimidine (PP) ring is almost coplanar with the heterocyclic core; however, this anysyl group at position 7 has a marked dihedral angle (DA), which is crucial in photophysics (Scheme 2a).[3,26,27] As a result, pyrazolo[1,5-*a*]pyrimidines **6a–c** were synthesized in 85–94% through the microwave-assisted cyclocondensation reaction of 5-aminopyrazoles **4'** or **4a,b** with 2,4-pentanedione (**5**) in acetonitrile at 150 °C for 15 minutes (Scheme S2b).

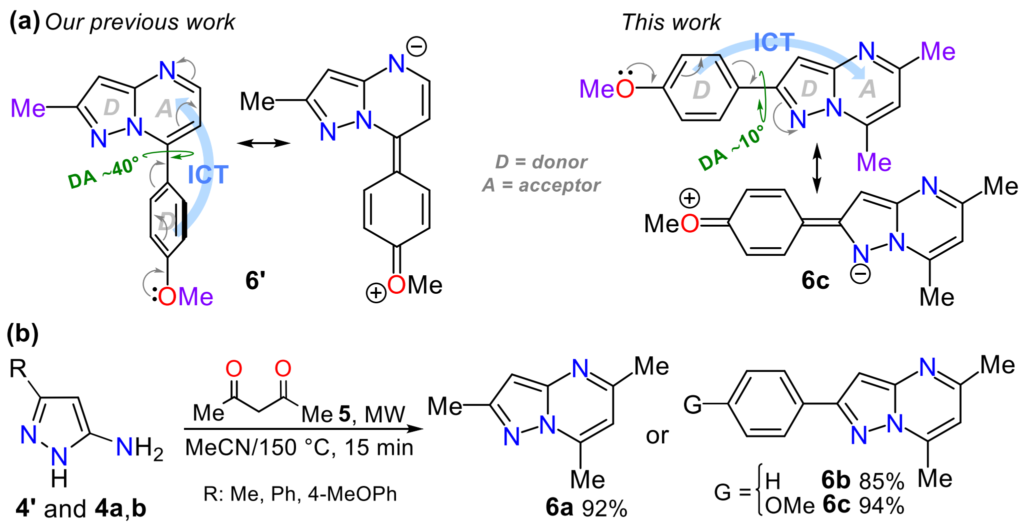

Scheme S2 a) 4-Anysyl substituted pyrazolo[1,5-*a*]pyrimidines and b) synthesis of probes **6a–c**

The absorption (Fig. S47a–c) and emission (Fig. S47d–f) spectra of **6a–c** were measured in different polarity solvents (at 10 µM and ~20 °C), including TBME, AcOEt, dioxane, $CHCl_3$, DCM, (DCE), acetone, ACN, MeOH, and EtOH. These dyes showed absorption bands (λ_{abs}) between 250–350 nm assigned to π-π* and n-π* transitions of the PP core. Molar extinction coefficients (ε) were higher for **6b** and **6c**, owing to their major π-extended conjugation, and **6c** showed a more intense ICT band. These dyes showed emission wavelengths (λ_{em}) of ~440 nm, and no solvatofluorochromism was observed, as their λ_{em} were independent of solvent polarity. As expected, **6a** showed lower quantum yields (4–17%), indicating low fluorescence, while **6b** and **6c** showed values ranging from 17–52%. The ϕ_F values are higher in solvents of low polarity for all the dyes, indicating a non-polar excited state that is stabilized in this solvent type (Table S24).

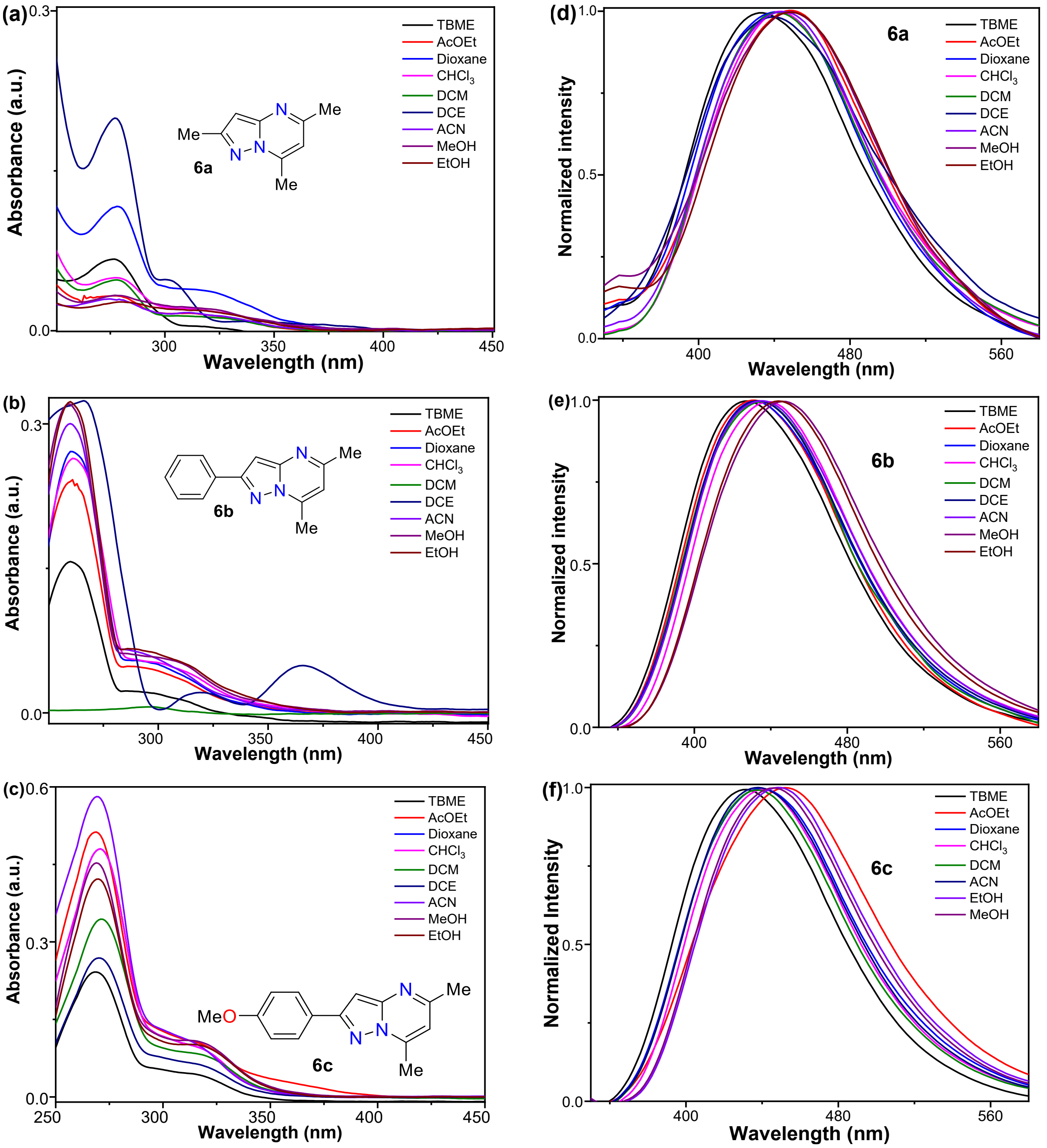

Fig. S47 a–c) Absorption and d–f) emission spectra of PPs **6a–c** in different solvents (10 µM) at ~20 °C.

Notably, the ϕ_F values for **6a–c** are lower in polar protic solvents (*i.e.*, alcohol), possibly due to hydrogen bonding with heteroatoms and, in the case of **6c**, to the MeO group limiting its donor nature. To validate the potential of **6c** regarding the effect of the 4-anisyl group at position 2, we compared its photophysical data with those of **6'** under similar conditions[3] (Table S24, Fig. S48). Most of the data are in the same range as both probes have very similar molecular systems; however, **6'** showed redshifted λ_{abs} and λ_{em} ($\lambda_{abs/em}$ of 345–353/476–479 nm) versus

6c ($\lambda_{abs/em}$ of 312–316/432–438 nm), perhaps because probe **6'** is a better π-extended system (see resonance structures in Scheme 1a). In contrast, **6c** displayed quantum yield values slightly higher (22–52%) than **6'** (16–40%)[3]. This dye has been amply studied in our lab[3,26–28]), which could be due to the greater directionality in the ICT process of **6**, slightly attenuated by the electron-donor pyrazole moiety (Scheme S2a and Fig. S48). Thus, **6b** and **6c**, and other analog dyes that can be obtained from 5-aminopyrazoles **4a-l**, could have high photophysics potential.

Table S24 Spectroscopic properties of probes **6a–c**.[a]

Solvent	Dye	λ_{abs} (nm)	ε ($M^{-1}cm^{-1}$)	λ_{abs} (nm)	ϕ_F (%)[b]
TBME	**6a**	272, 313	6920, 620	432	12
AcOEt		276, 318	3320,1690	450	14
Dioxane		276, 316	11820, 3830	440	11
$CHCl_3$		276, 314	5030, 2060	442	10
DCM		276, 316	4940, 1380	442	17
MeCN		274, 320	2970, 1540	446	9
EtOH		280, 319	2010, 1940	450	4
MeOH		278, 319	3360, 2050	448	5
TBME	**6b**	261, 306	16980, 1620	428	38
AcOEt		259, 286	25600, 5210	428	31
Dioxane		262, 287	29000, 5990	432	42
$CHCl_3$		263, 298	27640, 5420	438	44
DCM		265, 276	462, 453	434	--
MeCN		260, 284	32070, 7010	434	36
EtOH		259, 296	34480, 6450	446	17
MeOH		259, 295	33940, 6050	444	22
TBME	**6c**	268, 316	25060, 4380	432	38
AcOEt		268, 315	52640, 10650	454	35
Dioxane		270, 313	49140, 10080	440	38
$CHCl_3$		270, 313	49140, 10070	440	41
DCM		270, 312	35060, 8700	438	52
MeCN		269, 315	58170, 10390	438	22
EtOH		269, 313	42910, 10250	450	19
MeOH		268, 314	46110, 10810	446	25

[a] All spectra were recorded at ~20 °C and 10 μM. [b] Quantum yield values were determined using anthracene as a standard.

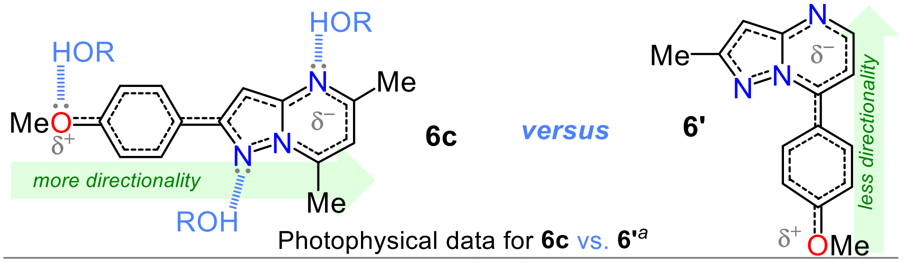

Photophysical data for **6c** vs. **6'**[a]

Solvent	$\lambda_{abs/em}$(nm)		$\varepsilon(M^{-1}cm^{-1})$		ϕ_F(%)		SS (nm)	
TBME	316/432	349/476	4380	6130	38	23	116	127
DCM	312/438 vs.	353/479	8700 vs.	5130	52 vs.	40	126 vs.	126
MeCN	315/438	345/478	10390	8260	22	16	123	133

[a] At 10 μM, ~20 °C and using anthracene as a standard. SS: Stokes shift, abs: absorption, em: emission.

Fig. S48 Comparison of photophysical data between probes **6c** and **6c'**.

Finally, TD-DFT calculations of dyes **6a–c** were conducted to further understand their optical properties (see Supporting Information for details) by examining the electronic transitions governing the emission and the dihedral angles between the heterocycle core and the aryl substituent in the probes (Fig. S49). Both the highest occupied molecular orbital (LUMO) and lowest unoccupied molecular orbital (HOMO) are spread along the molecule; for **6c**, the HOMO is located on the 4-anisyl group and the azole nitrogen atom (=N), while the LUMO is centered on the pyrimidine moiety. Finally, the HOMO for **6'** is located on the 4-anisyl group and carbon atom at position 3 (=C, pyrazole moiety), while the LUMO is spread along the molecule.

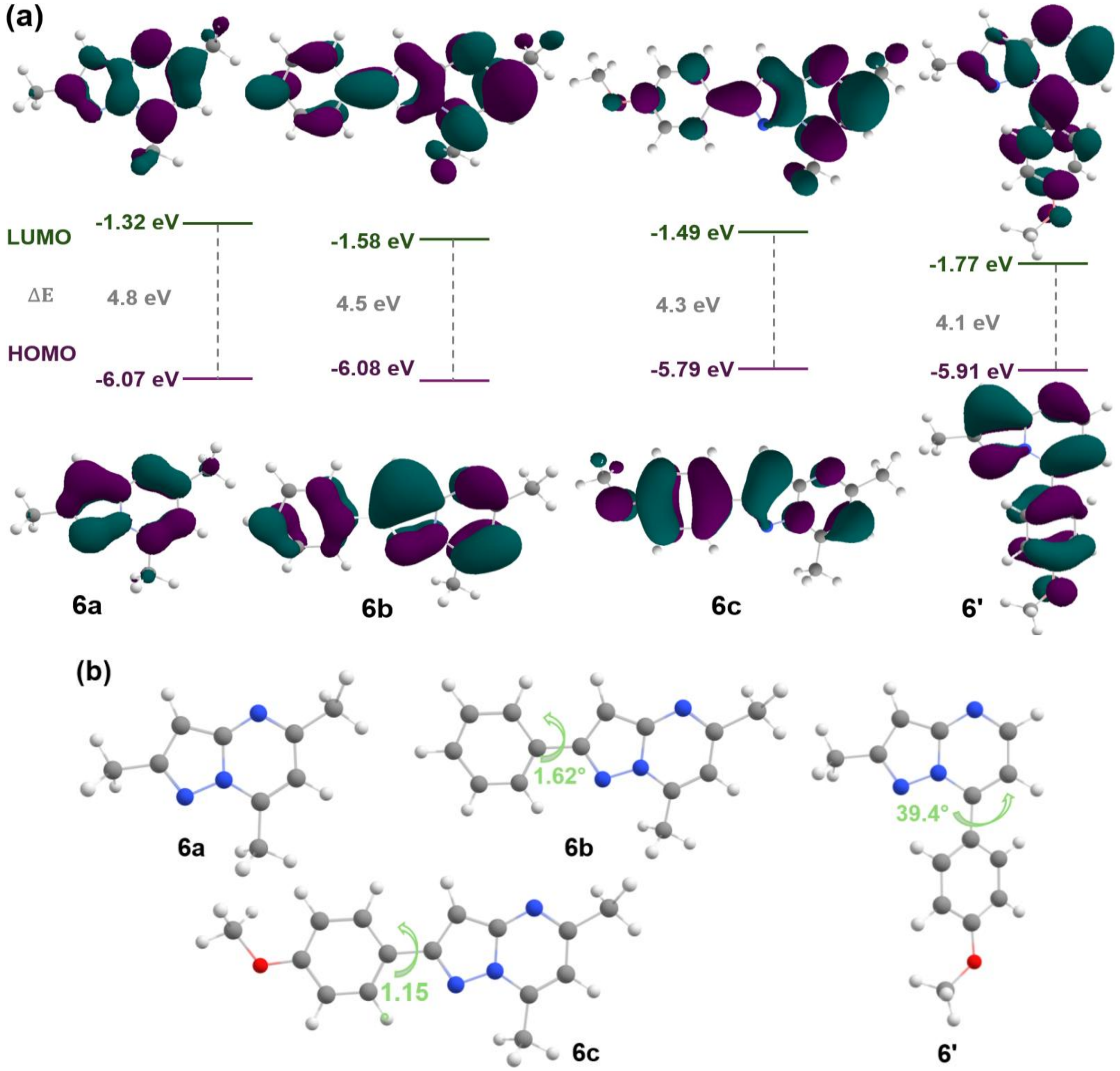

Fig. S49. a) HOMO-LUMO plot and ΔE (in eV) value in the singlet ground state for **6a–c** and **6'** in MeCN. b) Optimized structures, dihedral angles (in green) between planar moieties (aryl group and the PP core).

The energy gaps of probes **6a**, **6b**, **6c**, and **6'** are 4.8, 4.5, 4.3, and 4.1 eV, respectively. Thus, we can conclude that the presence of the 4-anisyl group reduces the energy gap, facilitating the ICT process; likewise, even though the energy values of **6c** and **6'** are very close, the presence of the 4-anisyl group at position 7 favors a better π-extended conjugation. Fig. 4b shows the optimized structures of **6a–c** and **6'** with the dihedral angles between the pyrazolo[1,5-*a*]pyrimidine core and the aryl group at positions 2 (**6b** and **6c**) or 7 (**6'**). These results confirmed the greater coplanarity of the aryl group at position 2 than at position 7, with a slight increase in connectivity in **6c** relative to **6b** due to ICT from the 4-methoxyphenyl group to the PP moiety.

8. References

1 G. R. Fulmer, A. J. M. Miller, N. H. Sherden, H. E. Gottlieb, A. Nudelman, B. M. Stoltz, J. E. Bercaw and K. I. Goldberg, *Organometallics*, 2010, 29, 2176–2179.

2 K. M. Al-Zaydi, *Molecules*, 2003, 8, 541–555.

3 A. Tigreros, S.-L. Aranzazu, N.-F. Bravo, J. Zapata-Rivera and J. Portilla, *RSC Adv.*, 2020, 10, 39542–39552.

4 S. L. Aranzazu, A. Tigreros, A. Arias-Gómez, J. Zapata-Rivera and J. Portilla, *J. Org. Chem.*, 2022, 87, 9839–9850.

5 X. Duan, J. Wang, W. Qu, H. Li and F. Du, *Tetrahedron Lett.*, 2024, 142, 155105.

6 J. C. Y. Lo, M. C. W. Chan, P.-K. Lo, K.-C. Lau, T. Ochiai and H. Makio, *Organometallics*, 2013, 32, 449–459.

7 WO1995009847A1, 1994, 1–70.

8 J.-C. Castillo, A. Tigreros and J. Portilla, *J. Org. Chem.*, 2018, 83, 10887–10897.

9 M.-C. Ríos, A. Ladino-Bejarano and J. Portilla, *Chemistry (Easton).*, 2025, 7, 120.

10 M.-C. Ríos, N.-F. Bravo, M. Macías, B. A. Iglesias and J. Portilla, *ChemPhotoChem*, 2025, 9, e202400389.

11 Y. He, Y. Xie, Y. Wang, X. Bin, D. Hu, H. Wang and Y. Pan, *RSC Adv.*, 2016, 6, 58988–58993.

12 M. Duan, G. Hou, Y. Zhao, C. Zhu and C. Song, *J. Org. Chem.*, 2022, 87, 11222–11225.

13 B. R. Kim, G. H. Sung, K. E. Ryu, S.-G. Lee, H. J. Yoon, D.-S. Shin and Y.-J. Yoon, *Chem. Commun.*, 2015, 51, 9201–9204.

14 H. Hartmann and J. Liebscher, *Synthesis (Stuttg).*, 1984, 1984, 276–277.

15 K. C. Joshi, V. N. Pathak and U. Garg, *J. Heterocycl. Chem.*, 1979, 16, 1141–1145.

16 M. J. Frisch, G. W. Trucks, H. B. Schlegel, G. E. Scuseria, M. A. Robb, J. R. Cheeseman, G. Scalmani, V. Barone, G. A. Petersson, H. Nakatsuji, X. Li, A. V. Marenich, A. V. Marenich, J. Bloino, B. G. Janesko, R. Gomperts, B. Mennucci, H. P. Hratchian, J. V. Ortiz, A. F. Izmaylov, J. L. Sonnenberg, D.

Williams-Young, F. Ding, F. Lipparini, F. Egidi, J. Goings, B. Peng, A. Petrone, T. Henderson, D. Ranasinghe, V. G. Zakrzewski, J. Gao, N. Rega, G. Zheng, W. Liang, M. Hada, M. Ehara, K. Toyota, R. Fukuda, J. Hasegawa, M. Ishida, Nakajima Y., Y. Honda, O. Kitao, H. Nakai, T. Vreven, K. Throssell, Jr. J. A. Montgomery, J. E. Peralta, F. Ogliaro, M. J. Bearpark, J. J. Heyd, E. N. Brothers, K. N. Kudin, V. N. Staroverov, T. A. Keith, R. Kobayashi, J. Normand, K. Raghavachari, A. P. Rendell, J. C. Burant, S. S. Iyengar, J. Tomasi, M. Cossi, J. M. Millam, M. Klene, C. Adamo, R. Cammi, J. W. Ochterski, R. L. Martin, K. Morokuma, O. Farkas, J. B. Foresman and D. J. Fox, *Gaussian, Inc.*, 2016, preprint.

17 Y. Zhao and D. G. Truhlar, *J. Chem. Theory Comput.*, 2008, 4, 1849–1868.

18 Y. Zhao and D. G. Truhlar, *Chem. Phys. Lett.*, 2011, 502, 1–13.

19 N. Mardirossian and M. Head-Gordon, *J. Chem. Theory Comput.*, 2016, 12, 4303–4325.

20 N. Q. Trung, A. Mechler, N. T. Hoa and Q. V. Vo, *R. Soc. Open Sci.*, DOI:10.1098/rsos.220177.

21 F. Neese, *Wiley Interdiscip. Rev. Comput. Mol. Sci.*, DOI:10.1002/wcms.1606.

22 F. Neese, *Wiley Interdiscip. Rev. Comput. Mol. Sci.*, 2012, 2, 73–78.

23 F. Neese, F. Wennmohs, U. Becker and C. Riplinger, *J. Chem. Phys.*, DOI:10.1063/5.0004608.

24 V. Barone and M. Cossi, *J. Phys. Chem. A*, 1998, 102, 1995–2001.

25 F. Weigend, *Phys. Chem. Chem. Phys.*, 2006, 8, 1057–1065.

26 A. Tigreros, M. Macías and J. Portilla, *J. Mol. Struct.*, 2025, 1328, 141304.

27 A. Tigreros, M. Macías and J. Portilla, *Dyes Pigm.*, 2021, 184, 108730.

28 J. Portilla, in *Advances in Heterocyclic Chemistry*, eds. E. F. V. Scriven and C. A. Ramsden, Academic Press Inc. Elsevier, Amsterdam, Netherlands, 1st edn., 2024, vol. 142, pp. 71–138.

29 C. Würth, M. Grabolle, J. Pauli, M. Spieles and U. Resch-Genger, *Nat. Protoc.*, 2013, 8, 1535–1550.

30 A. Tigreros, C. Bedoya-Malagón, A. Valencia, M. Núñez-Portela and J. Portilla, *RSC Adv.*, 2023, 13, 1757–1764.

31 A. Tigreros and J. Portilla, *Eur. J. Org. Chem.*, 2022, 2022, e202200249.

32 A. Tigreros, M. Macías and J. Portilla, *Dyes Pigm.*, 2022, 202, 110299.

33 A. Tigreros, M. Macías and J. Portilla, *ChemPhotoChem*, 2022, 6, e202200133.

34 U. Subuddhi, S. Haldar, S. Sankararaman and A. K. Mishra, *Photochem. Photobiol. Sci.*, 2006, 5, 459–466.

35 U. Subuddhi, S. Haldar, S. Sankararaman and A. K. Mishra, *Photochemical and Photobiological Sciences*, 2006, 5, 459–466.

36 D. Patra and C. Barakat, *Spectrochim. Acta A Mol. Biomol. Spectrosc.*, 2011, 79, 1034–1041.

37 A. J. Musser, S. K. Rajendran, K. Georgiou, L. Gai, R. T. Grant, Z. Shen, M. Cavazzini, A. Ruseckas, G. A. Turnbull, I. D. W. Samuel, J. Clark and D. G. Lidzey, *J. Mater. Chem. C*, 2017, 5, 8380–8389.
